# Large-scale field phenotyping using backpack LiDAR and CropQuant-3D to measure structural variation in wheat

**DOI:** 10.1093/plphys/kiab324

**Published:** 2021-07-16

**Authors:** Yulei Zhu, Gang Sun, Guohui Ding, Jie Zhou, Mingxing Wen, Shichao Jin, Qiang Zhao, Joshua Colmer, Yanfeng Ding, Eric S. Ober, Ji Zhou

**Affiliations:** 1 State Key Laboratory of Crop Genetics and Germplasm Enhancement, College of Engineering, College of Agriculture, Plant Phenomics Research Center, Academy for Advanced Interdisciplinary Studies, Jiangsu Collaborative Innovation Center for Modern Crop Production Co-sponsored by Province and Ministry, Nanjing Agricultural University, Nanjing 210095, China; 2 Zhenjiang Institute of Agricultural Science in Hill Area of Jiangsu Province, Jurong 212400, China; 3 National Center for Gene Research, CAS Center for Excellence in Molecular Plant Sciences, Chinese Academy of Sciences, Shanghai 200233, China; 4 Earlham Institute, Norwich Research Park, Norwich NR4 7UH, UK; 5 Cambridge Crop Research, National Institute of Agricultural Botany (NIAB), Cambridge CB3 0LE, UK

## Abstract

Plant phenomics bridges the gap between traits of agricultural importance and genomic information. Limitations of current field-based phenotyping solutions include mobility, affordability, throughput, accuracy, scalability, and the ability to analyze big data collected. Here, we present a large-scale phenotyping solution that combines a commercial backpack Light Detection and Ranging (LiDAR) device and our analytic software, CropQuant-3D, which have been applied jointly to phenotype wheat (Triticum aestivum) and associated 3D trait analysis. The use of LiDAR can acquire millions of 3D points to represent spatial features of crops, and CropQuant-3D can extract meaningful traits from large, complex point clouds. In a case study examining the response of wheat varieties to three different levels of nitrogen fertilization in field experiments, the combined solution differentiated significant genotype and treatment effects on crop growth and structural variation in the canopy, with strong correlations with manual measurements. Hence, we demonstrate that this system could consistently perform 3D trait analysis at a larger scale and more quickly than heretofore possible and addresses challenges in mobility, throughput, and scalability. To ensure our work could reach non-expert users, we developed an open-source graphical user interface for CropQuant-3D. We, therefore, believe that the combined system is easy-to-use and could be used as a reliable research tool in multi-location phenotyping for both crop research and breeding. Furthermore, together with the fast maturity of LiDAR technologies, the system has the potential for further development in accuracy and affordability, contributing to the resolution of the phenotyping bottleneck and exploiting available genomic resources more effectively.

## Teaser

CropQuant-3D and backpack LiDAR enable large-scale field phenotyping and 3D trait analysis to quantify structural responses to different nitrogen treatments in wheat.

## Introduction

With the rising world population, crop production needs to double by 2050 (UN Food and Agriculture Organization, 2009). To address this growing challenge of global food security, it is important to identify plants with desired traits to improve yield, resource use efficiency, quality, stress resistance and adaptation, and with a smaller environmental footprint (Powlson et al., 2014; Zhang et al., 2018; Swarbreck et al., 2019). Furthermore, the stability of the selected traits must be verified in the field over multiple seasons and locations (Sadras and Slafer, 2012; Griffiths et al., 2015; Reynolds and Langridge, 2016). For example, quantitative measurements of yield-related traits such as plant height, growth rate, canopy coverage, and spikes per unit area can be used to indicate and explain variations in yield stability in different environments (Sadras and Richards, 2014; Valluru et al., 2017; Furbank et al., 2019). In recent years, the cost of genotyping has decreased dramatically, allowing genetic analysis of large populations (Cobb et al., 2013; Crain et al., 2016). However, field phenotyping on a large-scale under realistic field conditions remains the bottleneck in genotype–phenotype association studies for crop improvement (Furbank and Tester, 2011; Yang et al., 2020). Both large-scale data acquisition and analysis of multiple traits at different time points and trial locations are still challenging, but often it is the meaningful phenotypic information most needed by breeders and crop researchers (Fiorani and Schurr, 2013; Tardieu et al., 2017; Furbank et al., 2019).

To relieve this bottleneck and address challenges in field phenotyping, much attention has been placed upon the applications of remote sensing, Internet of things, robotics, computer vision, and machine learning, resulting in a rapid technical progress in recent years (Pieruschka and Schurr, 2019; Zhao et al., 2019; Yang et al., 2020). A range of solutions have been developed, including the use of unmanned aerial vehicles (UAVs) and manned light aircraft for studying performance-related traits across fields (Bauer et al., 2019; Holman et al., 2019; Harkel et al., 2020); stationary gantry systems for deep phenotyping in fixed areas (Vadez et al., 2015; Kirchgessner et al., 2017; Virlet et al., 2017; Burnette et al., 2018); ground-based vehicles equipped with integrated sensor arrays to study canopy-related traits (Deery et al., 2014; Barker et al., 2016; Jimenez-Berni et al., 2018); hand-held or distributed sensing devices to measure various phenotypes during key growth stages (Hirafuji and Yoichi, 2011; Crain et al., 2016; Zhou et al., 2017b; Reynolds et al., 2019a). These methods possess diverse advantages and disadvantages concerning throughput, accuracy, mobility, affordability, scalability, and, more importantly, biological relevance (Fritsche-Neto and Borém, 2015; Furbank et al., 2019; Pieruschka and Schurr, 2019; Reynolds et al., 2019b; Roitsch et al., 2019). The selection of a phenotyping approach is naturally depending on the nature of the research question; but despite the rapid methodological progress, gaps in large-scale field solutions remain.

Among recent field-based solutions, Light Detection and Ranging (LiDAR) has attracted much attention as it provides information on plant morphological and structural features that are difficult or costly to quantify through traditional approaches (Lin, 2015; Stovall et al., 2017). As an active remote sensing technique, LiDAR computes the distance from laser scanners to a given target using pulsed laser beams, through which three-dimensional (3D) geometric features of the targeted object can be recorded in point cloud datasets (Arnó et al., 2013). LiDAR-based tools have been successful in overcoming issues related to natural illumination and occlusion, which have been problematic for many field-based methods (Sun et al., 2018; Jin et al., 2019). Although point clouds produced by LiDAR can be subject to noise and imbalanced densities (Bucksch et al., 2009), recently developed open-source analysis libraries such as WhiteboxTools (Lindsay, 2016) and Open3D (Zhou et al., 2018) can be utilized to conduct point clouds processing. However, these libraries were developed for generic 3D analysis, which requires experienced developers with a computer vision background to develop tailored solutions to analyze specific LiDAR data, limiting their use by plant researchers.

LiDAR devices can be roughly classified into three types: airborne, fixed terrestrial, and mobile (Hosoi and Omasa, 2009; Lin, 2015). Plant characters that have been estimated include crop height, biomass, and canopy structure (Omasa et al., 2007; Naito et al., 2017; Harkel et al., 2020); leaf number, shape, and the plant capacity to intercept solar radiation (Sun et al., 2018; Jin et al., 2019); and grain yield (Jimenez-Berni et al., 2018; Li et al., 2020b). LiDAR-generated point clouds have also been used to improve parameterization of crop models, enabling *in silico* testing to optimize trait combinations in breeding and crop growth simulation (Reynolds and Langridge, 2016; Wang et al., 2017; Walter et al., 2019). In comparison with alternative approaches that can also record 3D plant traits such as Structure from Motion (SfM) (DuAn et al., 2016), time-of-flight (Paulus, 2019), micro-computed tomography (Wu et al., 2019), and photogrammetry techniques (An et al., 2016; HolmAn et al., 2016), LiDAR provides a more reliable solution in scalability and accuracy for high-throughput field studies.

Despite these advantages, there are several problems associated with current LiDAR techniques in field phenotyping. Airborne LiDAR (Li et al., 2015; Harkel et al., 2020) typically requires larger multi-rotor UAVs with sufficient payload capacity (normally >5 kg), which requires a special trained pilot and local aviation authority’s clearance, adding to hardware and operating costs. Also, big drones generate strong downdraft that disrupts canopies when flying them at low altitudes to acquire high-resolution imagery. Fixed terrestrial LiDAR (Omasa et al., 2007; Stovall et al., 2017; Guo et al., 2018), on the other hand, is placed closer to plants and can generate high-resolution models. Nevertheless, this type of system requires more time to set up, limiting its applications in large-scale phenotyping. Mobile LiDAR (Arnó et al., 2013; Araus and Cairns, 2014; Deery et al., 2014; Jimenez-Berni et al., 2018; Deery et al., 2020) includes handheld, backpack, and devices mounted on specializ ed vehicles (e.g. Phenomobile), which can cover large trial areas. The main drawbacks of vehicle-mounted LiDAR are the costs of purchasing hardware, operating and maintenance, as well as the ability to access agricultural fields with difficult conditions or rugged terrain. Handheld LiDAR devices are lightweight and easy-to-use, but usually are equipped with low-cost laser sensors, limiting their capability to carry out high-quality and large-scale 3D mapping (Hyyppä et al., 2020; Jin et al., 2021).

The backpack LiDAR (Masiero et al., 2018; Hyyppä et al., 2020; Su et al., 2020) has been applied successfully to forestry studies and land surveillance in recent years, showing promise for field-based crop research. Compare with other LiDAR systems, it has good mobility, is relatively lightweight (normally around 10 kg), and is highly integrated with hardware, which means that it is easy to operate and maintain. Because the laser scanner can be used in close proximity to plants (<3 m), it can generate high-quality 3D models with up to 10 mm precision with high-end laser sensors. Depending on the laser scanner equipped, the backpack LiDAR system could have an effective scan range of over 200 m, useful for phenotyping in forestry or orchard plantations, as well as large experimental areas for plants. Backpack LiDAR also provides an accurate spatial positioning system (i.e. a global navigation satellite system), customized for field mapping at walking speed to enable an accurate 3D reconstruction (Masiero et al., 2018). As LiDAR technology has been maturing rapidly in recent years, it is expected that costs will decrease and this type of equipment could become more accessible for the research community (Guo et al., 2018; Panjvani et al., 2019; Jin et al., 2021). Still, the analytic software for LiDAR-based technologies is as important as the hardware. One limitation of many LiDAR-based mapping systems is the lack of widely available, open analytical software solutions that can extract biologically relevant information from the large point cloud data (Lin, 2015; Zhao et al., 2019; Yang et al., 2020), preventing nonexpert users from taking advantage of this technology for rapidly modeling crop structural features and mining phenotypic information to study spatial and temporal changes (Ubbens et al., 2018; Panjvani et al., 2019; Ward et al., 2019).

Here, we introduce an integrated solution that combines a backpack LiDAR device with open-source analytic software called CropQuant-3D for processing large-scale field phenotyping and 3D trait analysis. The software employs 2D/3D image analysis algorithms and Discrete Fourier Transform (DFT) to derive plot-based measurements of key performance-related traits such as crop height and structural variation in the canopy. We developed a range of technical applications to integrate the backpack LiDAR and CropQuant-3D into field-based phenotyping, including a large-scale mapping protocol for cereal crops, the quick quality assessment of collected datasets at different sites, and a comprehensive analysis pipeline. In a case study of wheat (*Triticum aestivum*), we describe the integrated solution to quantify varietal responses to three levels of nitrogen (N) fertilization of eleven Chinese winter wheat varieties selected from the “Zhenmai” and “Ningmai” populations. By combining 3D trait analysis and manual key yield components, we also produced a performance matrix to rank and evaluate genotypic differences in N responses for the examined varieties, resulting in the classification of four N response types. To ensure that our work could reach the broader research community, we have developed a graphical user interface (GUI) for CropQuant-3D so that nonexpert users could use the software easily. Furthermore, we expanded the software package to analyze point clouds generated from other sources such as gantry-mounted LiDAR and UAV-SfM photogrammetry. We uploaded the CropQuant-3D software (in EXE format), executable analysis source code (in Jupyter notebooks), and testing datasets to our GitHub repository, which are openly available for plant research community. Hence, we believe that the integrated solution presented here is capable of addressing challenges in mobility, throughput, scalability, and enabling us to analyze big LiDAR-collected 3D point cloud data, which is likely to help plant researchers bridge the gap between traits of agricultural importance and available genetic resources for crop improvement.

## Results

### In-field mapping protocol using the backpack LiDAR

**Figure 1:**
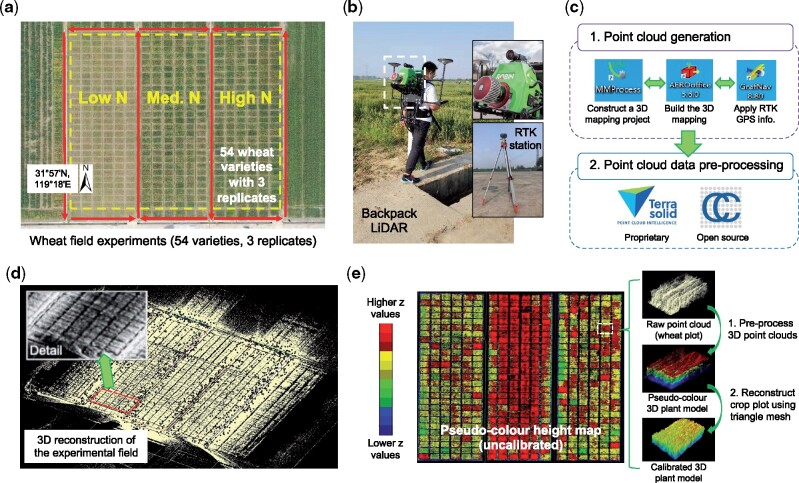
The data acquisition procedure using a backpack LiDAR device together with raw point cloud data generated through pre-processing a LiDAR-acquired 3D point cloud file. A, An overhead orthomosaic image of the field trial area showing 486 6-meter winter wheat varieties with three levels of N fertilization treatments (i.e. 0, 180, and 270 kg N ha^−1^). Red arrows represent the grid-style mapping method carried out by a LiDAR operator outside the plots. B, The backpack LiDAR device (ROBIN Precision) and a RTK base station are used for 3D field phenotyping. C, A high-level workflow of the pre-processing software used to generate RTK-tagged point cloud data collected by the backpack LiDAR. D, The raw point clouds generated for the trial area. E, Initial height-based analysis with uncalibrated 3D points, which were colored according to *z*-values, and example plot-level images using raw 3D points, height values, and triangle mesh.

Because limited research has been conducted on the use of backpack LiDAR in-field phenotyping, we, therefore, developed a range of technical applications to utilize the device in the field, including the optimal distance to map cereal crops, the design of mapping routes and angles, the quick assessment of the data quality, and the calibration method at different sites. For example, a grid-style mapping approach was designed to routinely map the large field trial in this study (red arrows in Figure 1A). We first recorded the 3D geo-coordinates of the trial area using a real-time kinematic (RTK) base station, which logged satellite-based positions with a ±5-mm error range in 3D (Figure 1B). Then, a LiDAR operator walked around the perimeter of each N treatment block in the field to map the entire experiment from different angles. Due to the scan range of the LiDAR device, we did not need to walk around each individual plot, saving significant time in operation. On average, it took the LiDAR operator 20–25 min to map an experiment field of 0.5-ha, equivalent to a mapping speed of around 1.2 ha/h. To study canopy structural responses to different N, we focused on the growth stages between heading (GS51–59) and grain filling (GS71–89) when the canopy was largely established (Zadocks et al., 1974).

### Data pre-processing to generate 3D point clouds

According to standard practice in processing 3D points (Kachamba et al., 2016; Duan et al., 2017; Sun et al., 2018), we used the bundled pre-processing software to generate GPS-tagged 3D point clouds collected by the LiDAR (Figure 1C). The bundled software we used are MMProcess to build up a 3D mapping project, AERO-office to define the mapping path, and GrafNav to associate RTK GPS signals with the path. To select, visualise, and export point clouds, we chose to use the open-source CloudCompare software (Girardeau-Montaut, 2015). The same tasks can also be accomplished by using proprietary software such as TerraSolid (Korzeniowska and Łącka, 2011).

Because the backpack LiDAR device we used has an effective scan range of around 200 m (over 180 million points were collected in a single field), the mapped area (over 1.5 ha; Figure 1D) was much larger than the experiment region (i.e. the combined area of the 486 wheat plots, 0.5 ha). Hence, we used RTK-recorded geo-coordinates to delineate regions of interest (ROI) and facilitate our routine processing. After defining the ROI (over 45 million points retained for the experimental region, around 90,000 points per plot), all 3D points were visualized and colored according to their *z* values (Figure 1E). A preview of uncalibrated 3D mapping data before terrain adjustment enabled us to (1) associate pseudo-color to raw 3D points for quick growth assessment, (2) perform initial comparisons of experiments at multiple sites, and (3) define ROI to facilitate field- and plot-level 3D points sampling.

### A comprehensive pipeline for traits analysis

**Figure 2:**
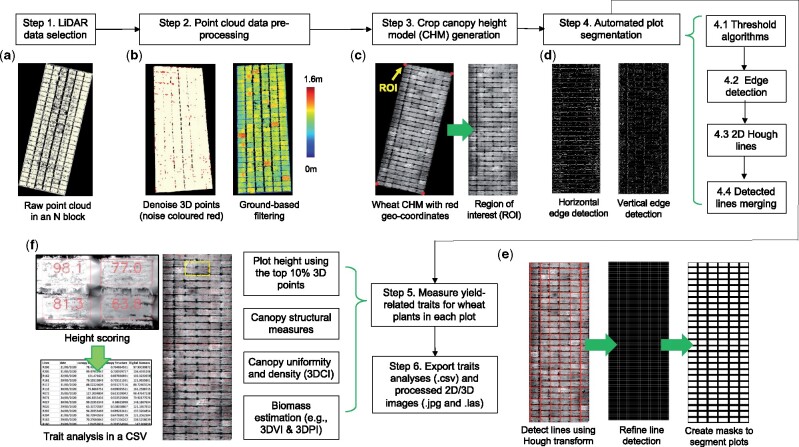
A high-level analysis pipeline established for processing LiDAR-acquired point clouds and measuring yield-related traits in 3D. A, Select a pre-processed point cloud file (in LAS format). B, Remove outliers (colored red) in the point cloud, followed by filtering methods to differentiate ground-based terrain (e.g. soil level below the crop) and above-ground (crops) 3D points. C, Generate a 2D CHM and define the ROI (denoted by the four red markers) using geo-coordinates collected by the ground-based RTK station. D and E, Detect horizontal and vertical edges using the Sobel operator, followed by the application of 2D Hough transform to produce a binary mask to segment plots in the field experiments. F, Measure and export 3D trait analysis results for each plot, including measured traits (CSV), processed images (JPG), and processed point cloud (LAS).

To carry out routine 3D points processing and trait analysis using LiDAR-collected point clouds, we developed a comprehensive analysis pipeline. Figure 2 shows a high-level workflow of the pipeline, which consisted of six steps: data selection, normalization, the generation of crop canopy height model (CHM), plot segmentation, 3D trait analysis, and export of the analysis results:

1. *Step 1*: a pre-processed point cloud file (in LAS format) was selected (Figure 2A). Because LiDAR-collected point clouds are likely to be noisy and uncalibrated (with slopes and terrain features of the field), we developed a process to normalize the 3D points (*Steps 2* *and* *3*). To remove noise, we followed a published method (Su et al., 2019), which calculates the average distance between a given 3D point and its neighboring points (*avg.*). If the distance (*k*) between the point and its neighboring points (defaulted to 50) is greater than *avg.* + *k* × *std.* (where *std.* is one standard deviation of the mean of all the distances), the point will be classified as an outlier. In our case, all identified outliers were colored red and removed from the following analysis (Figure 2B).
2. *Step 2*: after denoising, a filtering method was applied to separate ground-level and above-ground 3D points by applying the LidarGroundPointFilter function in WhiteboxTools (Lindsay, 2016), including (1) ground-based slope normalization, (2) a subsequent k-nearest neighbors (Lowe, 2004) to identify neighboring points within a defined *radius* (defaulted to 2) to examine height differences, and (3) a classification method to classify ground-level and above-ground points. The use of the function resulted in a flattened ground plane, enabling precise measurements of above-ground 3D points. The output of *Step 2* is saved in a new LAS file with all the ground-level points assigned with *zero z*-values (dark blue) and above-ground points assigned with height values in centimeter.
3. *Step 3*: a key step in the pipeline used to generate a CHM for 3D trait analysis. First, because the density of LiDAR-collected point clouds is likely to be unbalanced (e.g. denser 3D points for objects close to the laser scanner, Figure 1D), we improved a progressive triangulated irregular network (TIN) algorithm (Zhao et al., 2016) to interpolate the unbalanced point clouds. Then, we utilized all the filtered above-ground points to generate a digital surface model (DSM), followed by the conversion of geo-coordinates on the *x* and *y* axes into pixel coordinates (Ritter and Ruth, 1997) to define four ROI markers in the DSM (Figure 2C). When processing a series of point cloud files collected from the same field, these four markers could be used repeatedly. To reduce computational complexity, we associated *z*-values of each 3D point with a grayscale value (i.e. 0 cm is taken to be black and 160 cm is taken to be white; the taller the point, the higher the grayscale value), followed by a projection method to cast all 3D points onto the flattened ground plane. This process produced a 2D CHM image from an overhead perspective (Figure 2C). Finally, we performed a 2D perspective transform (Mezirow, 1978) using the getPerspectiveTransform function in OpenCV (Howse, 2013) to extract the region within the four markers and then align the CHM for automated trait analysis. The 2D CHM image contains spatial information of all the plots in the experimental field.
4. *Step 4*: to segment plots using the 2D CHM, we employed the 2D Hough transform (Duda and Hart, 1972) to detect plot boundaries. Because the gap between plots could be unclear during the season (e.g. lodging could cover the gap), missing pixels between plots or noise could affect the result of the Hough transform. Hence, we designed an improved method to detect horizontal and vertical lines separately (Figure 2D), including: (*Step 4.1*) combining both global (Sauvola and Pietikäinen, 2000) and local thresholding (Firdousi and Parveen, 2014) methods to establish an initial plot mask for the CHM, even if the background is not uniform; (*Step 4.2*) using the *Sobel* operator (Kroon, 2009) to detect the horizontal and vertical edges (angles were set at 360 and 30 as all the CHMs were aligned); (*Step 4.3*) drawing straight lines based on the detected edges (with right angles, *x*- and *y*-intercept as input parameters) using the hough_line and line_aa functions in Scikit-Image (van der Walt et al., 2014); (*Step 4.4*) merging multiple detected lines if they were close to each other, so that only a single line could represent the gap between plots (Figure 2E). Finally, assembling the lines and producing a final plot-level mask to present all of the plots in the field (e.g. 162 plots in Figure 2E). To remove edge effects, gaps within plots due to plant sampling, and crop variation that is not directly linked to the varieties or treatments (e.g. N loss), we calculated the weighted centroid of each plot using grayscale-based entropy features (Susan and Hanmandlu, 2013) within a given plot. Through this approach, the width and length of a plot mask could be adjusted adaptively to rectify the plot-level sampling areas.
5. *Steps 5* *and* *6*: the last two steps of the pipeline measured and exported key performance- and yield-related traits for each plot. A range of traits have been measured, including crop height, 3D canopy uniformity, 3D canopy surface, canopy coverage, and biomass estimation (i.e. 3D voxel index [3DVI] and 3D profile index [3DPI]). A table (in CSV format) was generated and populated with these scores, with each row corresponding to a plot (i.e. a variety) and each column corresponding to a trait, arranged according to the plot location (i.e. row and column IDs) in the field (Figure 2F).

### The GUI of CropQuant-3D

**Figure 3:**
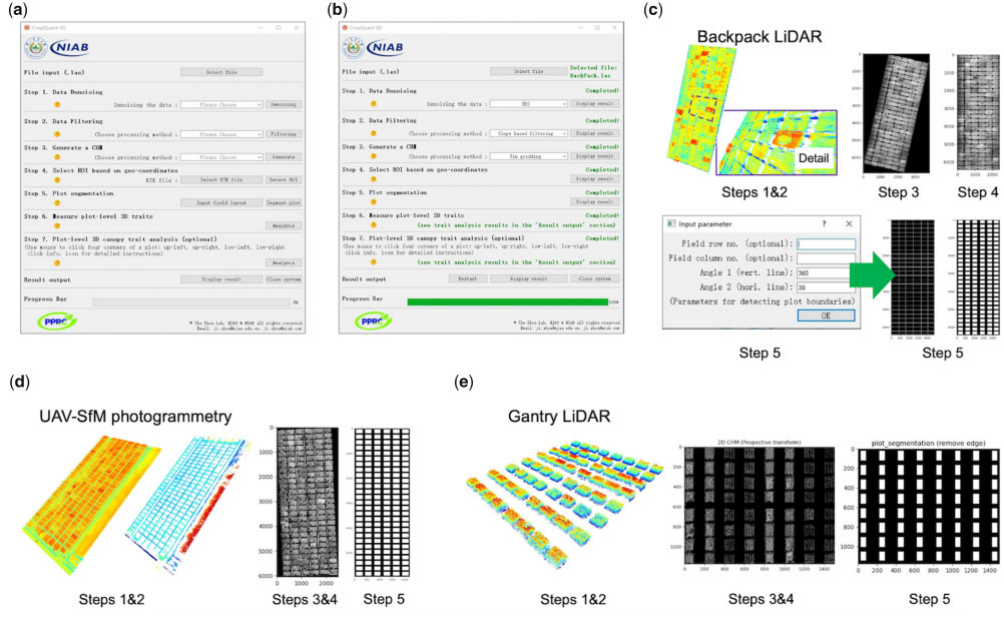
The GUI for CropQuant-3D was designed for processing 3D point cloud files using 2D/3D image analysis algorithms and mathematic transformation for analyzing canopy structural traits in 3D. A, The initial GUI window of CropQuant-3D. B, The GUI window after accomplishing all required analysis steps, with the progress bar showing 100%. C, The intermediate results that can be displayed for each processing step integrated with the analysis procedure for processing point cloud files generated by the backpack LiDAR, including optional input parameters such as the number of rows and columns of the experimental field that users could enter to assist the algorithm for segmenting plots. D, The intermediate results that can be displayed for processing point cloud files collected by UAV aerial imaging. E, The intermediate results that can be displayed for processing point cloud files generated by a gantry-mounted LiDAR system, FieldScan.

To facilitate nonexpert users to process 3D point clouds (in LAS format), we developed the GUI of CropQuant-3D, which integrated the above analysis pipeline into a single dialog panel, from which all the above algorithmic steps could be performed. The GUI was implemented using PyQt5, a comprehensive set of Python bindings for the Qt v5 library (Summerfield, 2015), allowing the GUI to be executable on varied operating systems (see Availability and requirements). Following a similar systems design described previously (Zhou et al., 2017a), CropQuant-3D uses a stepwise approach to process point clouds and analyze 3D traits. The initial window (Figure 3A) shows several sections with default input parameters pre-populated. In the input section, a user needs to select a LiDAR file (test LAS files provided on the GitHub). Then, the user needs to pre-process the selected point cloud file, including denoising and ground-based filtering (*Steps 1* and *2* in the GUI). After pre-processing, the user can generate a 2D CHM (*Step 3*) by defining the exchange rate between a pixel and a metric unit (i.e. cm), followed by defining geo-coordinates of the experimental field (i.e. ROI markers; *Step 4*). *Step 5* is to segment plots using the 2D CHM, so that traits such as plot-based height and canopy coverage can be measured (*Step 6*). Finally, if the user needs to export point clouds for specific plots, the user can click four corners of one or multiple plots in the CHM following the order: upper-left, upper-right, lower-left, and lower-right (*Step 7*, optional). To enable a fast selection of plot-level 3D points, we used the EVENT_LBUTTONDOWN function in OpenCV to create a mouse response event. The analysis results can be downloaded after all the mandatory steps are accomplished (Figure 3B).

When a step is finished, a green-colored message will be displayed in the section together with a Display button to show intermediate results (Figure 3C). In particular, if the plot boundaries are unclear and the plot segmentation algorithm fails to segment all the plots, the user can define the field layout (i.e. the number of rows and columns) through an optional input box, which will generate baselines to assist the plot segmentation. Furthermore, to enable the GUI software to process point clouds produced from other sources such as UAV-SfM photogrammetry and LiDAR mounted on gantry systems, we expanded the input function to accept these types of point cloud files (in LAS format). For example, the CropQuant-3D GUI can process point clouds generated by both UAVs (Figure 3D) and FieldScan^TM^ (Phenospex, Netherlands; Figure 3E) through unified analysis steps in the software to perform plot-based 3D trait analysis. A detailed step-by-step user guide (Supplemental Methods S1) and an instructional video (Supplemental Movie S1) for the GUI-based software can be seen in the Supplemental Data. The software implementation can be seen in the “Materials and Methods”.

### Height measurement using CropQuant-3D

**Figure 4:**
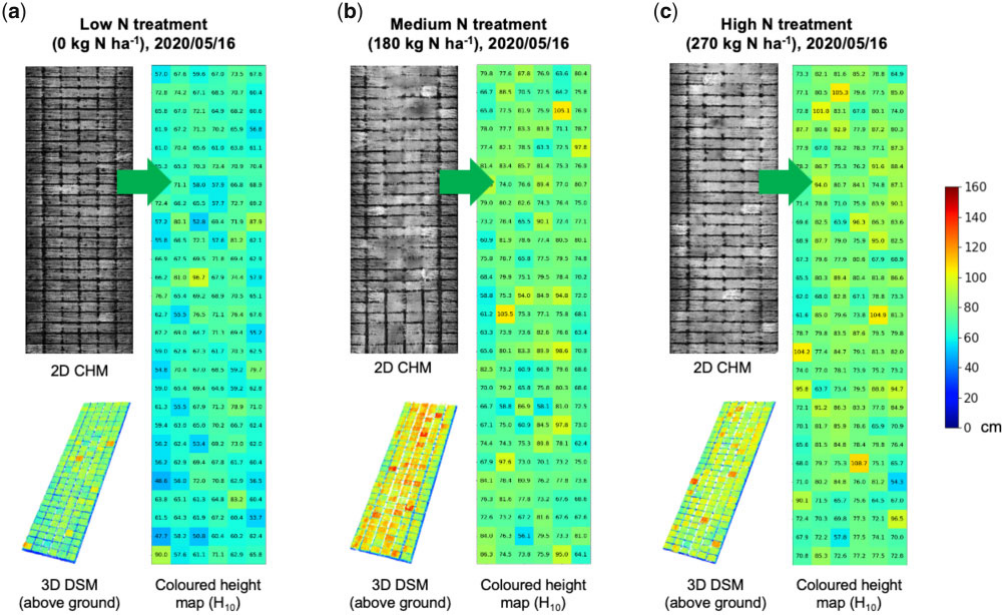
The pseudo-colored uncalibrated height maps, 3D visualization, and pseudo-colored calibrated height maps of NUE wheat experiments under three different levels of N treatments. A, The 2D CHM image (to the left) and 3D digital surface model (DSM) image, created using the RTK tagged altitude height values, and the calibrated height maps (to the right), showing the average height value of the highest 10% 3D points (H_10_) for the low-N treatment; (B, C) the 2D CHM, 3D DSM (left) and the calibrated height (right) images for the medium-N and high-N treatments. The unified height scale bar for the three sub-figures is shown.

Plant height and the rate of height increase (i.e. growth rate) are important performance- and yield-related traits (HolmAn et al., 2016; Nguyen and Kant, 2018; Momen et al., 2019). For field-based phenotyping, we found that, although terrain adjustment (e.g. slope removing) is a standard process for height estimates from elevation models in large-scale land surveillance and forestry research, there are no standardized approaches designed for such adjustment in relatively small-scale crop fields. Hence, we have implemented a customized solution to normalize slopes and terrain features before height mapping. To measure crop height in a given plot, our algorithm was partially based on a mobile laser scanning approach described previously (Friedli et al., 2016), but performed on a flattened ground plane (*Steps 3 *and *4* in the pipeline) with the highest 10% 3D points (H_10_) sampled in the plot to reduce height variances at the canopy level. The average height value of the H_10_ set was computed as the plot-level crop height. We produced three sets of height maps for all the six-meter 486 plots under three N treatments at the heading stage, with a unified height scale bar (Figure 4). The 3D DSM and 2D CHM images (Figure 4, A–C, left) show the 3D reconstruction and height distribution of the three N blocks, from 60-degree and overhead perspectives; whereas the colored height maps (Figure 4, A–C, right) demonstrate how the height of wheat plants responded to different levels of N treatments (Supplemental Table S1).

### 3D Canopy surface and canopy coverage measures

The rates of carbon gain through photosynthesis and water loss through transpiration of the canopy can be affected by changes in canopy structure, which can be used to explain crop performance and plants’ responses to the environment (Green et al., 1985; Shearman et al., 2005). However, it is challenging to measure canopy structural characters due to its complexity and dynamic spatial variability caused by genetic, agronomic management, and environmental effects (Omasa et al., 2007; Hosoi and Omasa, 2009; DuAn et al., 2016). Although LiDAR devices have been used to visualize 3D canopy structure, how to quantify structural changes using point clouds was still a challenge that needed to be addressed.

**Figure 5:**
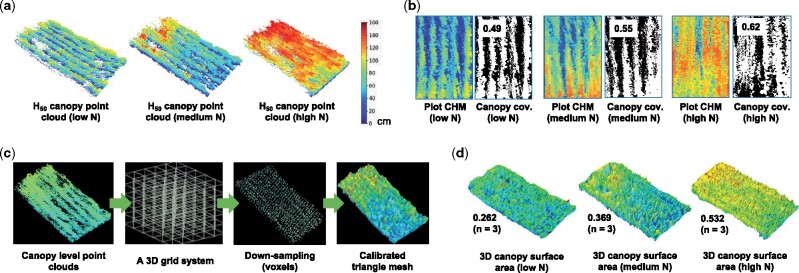
The analysis process of measuring 3D canopy surface area and canopy coverage at the plot level using voxels and triangular mesh for wheat varieties. A, 3D points for the canopy region using the highest 50% points (H_50_) in a given plot. B, H_50_ points projected onto the ground plane, generating pixels representing crop canopy regions, which were processed by an adaptive approach to calculate the normalized canopy coverage trait (0-1, where 1 stands for 100%). C, A brief analysis process of computing the 3D surface area trait using triangle mesh. D, The normalized 3D surface results (0-1, where 1 stands for maximum 3D surface area in a given plot) of a wheat variety under three N treatments.

We approached the matter by measuring a range of traits at the canopy level, including 3D canopy surface area and canopy coverage. To measure canopy coverage index, we developed the following steps: (1) retaining the highest 50% 3D points (H_50_) in a given plot (Figure 5A); (2) then, projecting H_50_ points onto a flattened plane to generate a 2D canopy image from an overhead perspective; (3) after that, applying the threshold_local function in Scikit-Image (Singh et al., 2012) to select pixels in the canopy image using the calculated local threshold, resulting in a binarized canopy mask to represent the canopy coverage in a plot. We applied the trait to measure the canopy coverage differences of a wheat variety (e.g. NMzi-1019) under three N treatments. The canopy coverage index (0-1, where 1 is 100% coverage) showed an increase of 10%–15% when the N fertilization increased (Figure 5B).

While the canopy coverage is important as it relates to the interception of direct solar radiation, it does not account for the total leaf area of the canopy, which is a more precise measure of interception of diffuse radiation and reflected light within the canopy (Cabrera-Bosquet et al., 2016). As the 3D surface area of the canopy would be closely related to the total transpirational leaf area and would correspond with the summed photosynthetic activity of all leaves (Omasa et al., 2007), we, therefore, included the measurement of 3D canopy surface area in the CropQuant-3D (Figure 5C). The algorithmic steps were designed based on the triangle mesh method (Edelsbrunner et al., 1983), including: (1) applying the voxelization method (Truong‐Hong et al., 2013) to generate a 3D grid system to package all the above-ground 3D points into voxels; (2) using the voxel_down_sample function from Open3D to down-sample the number of voxels, so that gaps between plants in a given plot could be covered; (3) using the create_from_point_cloud_alpha_shape function (Edelsbrunner et al., 1983) to reconstruct 3D surfaces of the canopy, followed by the get_surface_area function to calculate the 3D surface area. For example, the 3D surface area indices of wheat variety NMzi-1019 showed an increase of over 20% with the increase in N application levels (Figure 5D). In addition to the above two traits, we also integrated traits such as 3DVI and 3DPI into CropQuant-3D to estimate biomass, which has been described previously (Jimenez-Berni et al., 2018; Deery et al., 2020). All the above trait analysis results are listed in Supplemental Table S2.

### An original canopy structural measure—3D canopy index

**Figure 6:**
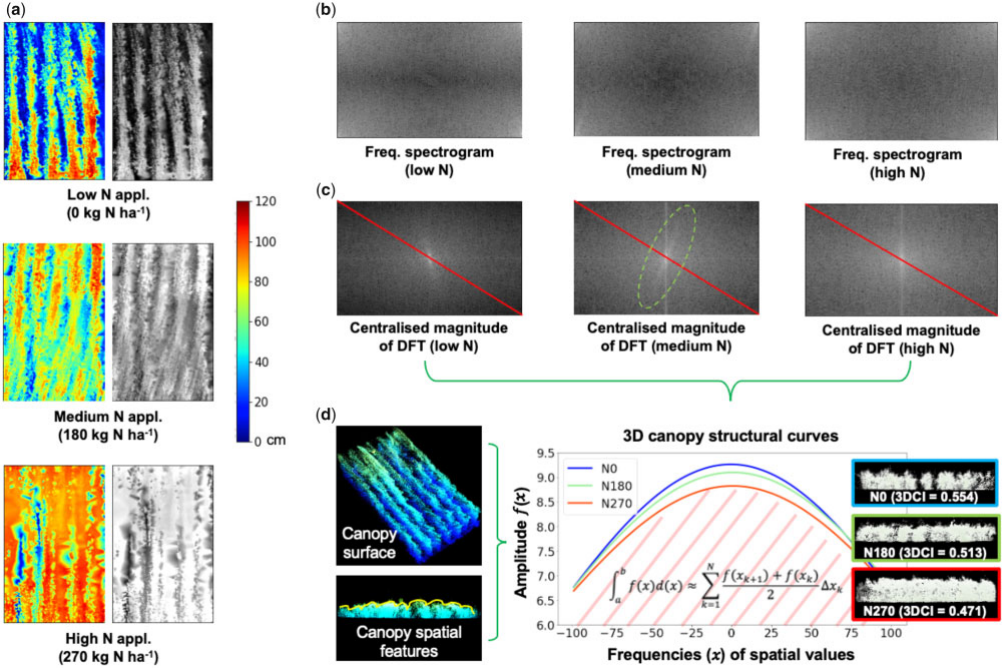
The analysis procedure of measuring 3D canopy structure at the plot level using 2D CHM images and a 2D DFT, resulting in 3D canopy structural curves for separating variety responses to different N treatments. A, The pseudo-colored height images and their associated grayscale height images (intensity values correspond to height values) in a plot, under three N treatments. B, Frequency spectrograms generated using 2D DFT of the grayscale height images, containing all frequencies of height values and their magnitude in the plot. C, Centralized magnitude of DFT produced to enable frequency and amplitude sampling through red-colored lines on the diagonal of the image; regular patterns observable in the images with medium- and high-N treatments. D, Three canopy structural curves plotted to present structural differences together with cross-sections of 3D points at the canopy level, showing the wheat variety’s different responses to three N treatments as well as the procedure of computing 3DCI (0-1, where 1 stands for maximum accumulated spatial variation in a given plot) based on the curves and areas beneath the curves.

While the above indices are useful measures to describe some canopy structural features, they do not convey information about canopy-level changes in spatial characteristics (e.g. height variation) across the plot, which are likely to be affected by many factors in the field experiments, including (1) plant architecture such as individual tillers (e.g. main stem is taller than secondary tillers), which could differ between genotypes, (2) the height of spikes if a mixed population was drilled, (3) the density of the crop (e.g. spikes number per unit area, SN m^−2^) due to different management practices such as the seeding rate, (4) agronomic or environmental reasons unrelated to treatment or genotype (e.g. local seedbed variations), and (5) lodging. We have established an original algorithm incorporated in the CropQuant-3D software to measure spatial differences at the canopy level. Following the previous naming convention (Jimenez-Berni et al., 2018), we called this measure 3D canopy index (3DCI). The algorithm for 3DCI consists of five key steps:

1. Using the plot-level masks (Figure 2E), we extracted all the above-ground 3D points in a given plot to generate a pseudo-color spatial map from an overhead view. We then transformed the map into a grayscale image with each pixel’s grayscale value corresponding to its height value, resulting in a 2D plot-level CHM (Figure 6A, right).
2. A 2D DFT method (Cooley and Tukey, 1965) was applied to represent the plot-level CHM in the frequency domain, producing the magnitude of the image’s Fourier transform. Because the dynamic range of the Fourier coefficients was too large to be visualized, we applied a logarithmic transform and generated a frequency spectrogram (Figure 6B), containing all frequencies of the spatial information in the plot and their magnitude. The DFT can be defined as:
  (1) $$f\left(x,y\right)=\frac{1}{MN}\sum_{u=0}^{M-1}\sum_{v=0}^{N-1}F(u,v)e^{j2\pi \left(\frac{ux}{M}+\frac{vy}{N}\right)};x=[0,M-1],y=[0,N-1]$$

where *f(x, y)* represents the M×N spatial domain matrix, and *F(u, v)* represents the DFT of *f(x, y)*. The coordinate system of *F(u, v)* is in the frequency domain. where *x* is frequencies of spatial values, *a* is the minimum frequencies of spatial values (set as −100), *b* is the maximum frequencies (set as 100), f(x) is the amplitude value after Gaussian fitting, *N* is the total number of x sampled, $\Delta x_{k}$ is the difference between $x_{k}$ and $x_{k+1}$.

1. We centralized the frequency spectrogram to remove periodic interference signals, resulting in a centralized magnitude image to represent the spatial information. For example, by applying DFT to CHM images under three N treatments, we could identify different structural features at the canopy level (Figure 6C): (1) the magnitude of the low-N magnitude image became rapidly smaller for higher grayscale values (e.g. canopy objects such as wheat spikes), suggesting its canopy was lower and the distribution of its spatial features was spread out (i.e. less dense) compared with crops under medium or high N treatments; (2) the main values of spectrogram images for both medium and high N applications lay on a vertical line, suggesting their canopy structures contained a dominating vertical orientation caused by regular patterns (e.g. lines formed by plants); and (3) in the medium-N magnitude image, another pattern could be observed which passed through the center at 75–80° angle (highlighted by a light-green dashed oval), which was caused by another spatial pattern in the plot and potentially could be a useful tool to measure the degree of lodging (Figure 6A).
2. To utilize the above DFT results in quantitative trait measurements, we sampled all the pixels’ grayscale values on the diagonal of the centralized magnitude image (red-colored lines in Figure 6C), based on which frequencies of all spatial values and their amplitude were summarized. We then used the Gaussian fitting to plot the amplitude of the sampled spatial values, producing curves to represent canopy structural features within a defined frequency region, where the *x*-axis denotes frequencies of canopy-level spatial values, and the *y*-axis represents their associated amplitude (Figure 6D). Two important features could be concluded from canopy structural curves: (1) the curvature of these curves, signifying the density of crop canopy, as a less dense canopy structure contained larger spatial variation (e.g. less dense spikes) and resulted in a higher curvature; (2) the area beneath the structural curve (e.g. with light red diagonal stripes, Figure 6D), showing the canopy uniformity—when curvatures are similar, structural curves comprise greater area indicates less uniformity due to greater accumulated spatial variances. We used integral calculus (i.e. integration) to compute the area beneath the canopy structural curve, which is defined by Equation 2:
  (2) $$\int_{a}^{b}f(x)d(x)\approx \sum_{k=1}^{N}\frac{f\left(x_{k+1}\right)+f(x_{k})}{2}\Delta x_{k};x\in [a,b],k=[1,N]$$

To compute the curvature of a structural curve, we used Equation 3 as described previously (Van Der Walt et al., 2011):

(3) $$\mathrm{Curvature}=\left(\left|\frac{d^{2}x}{dt^{2}}*\frac{dy}{dt}-\frac{dx}{dt}*\frac{d^{2}y}{dt^{2}}\right|\right)/{\left(\frac{dx}{dt}*\frac{dx}{dt}+\frac{dy}{dt}*\frac{dy}{dt}\right)}^{3/2}$$

where *x* represents the frequency array (the *x*-axis), *y* is the amplitude array (the *y*-axis). where *x* is the calculated value using Equation 2, *y* is the normalized 3D canopy uniformity index, *MinValue* is the theoretical minimum value from the value list, i.e. 59.3% of the calculated minimum value (Raybould and Quemada, 2010); and *MaxValue* is the theoretical maximum value from the value list, i.e. 129.4% of the calculated maximum value.

1. To use the above equations for measuring canopy uniformity, we normalized values generated by Equation 2, so that we could cross-validate the measure for different varieties. We called this normalized value 3DCI. The normalization is defined by Equation 4:
  (4) $$y=\frac{x-\mathrm{MinValue}}{\mathrm{MaxValue}-\mathrm{MinValue}}$$

To verify the 3DCI and curvature measures, we used the wheat variety NMzi-1019, which has been shown to respond strongly to different levels of N fertilization (Feng et al., 2008). Three canopy structural curves of NMzi-1019 under three N treatments (*n* = 9 plots) were produced (Figure 6D). The three curves’ curvatures reduced moderately when the N fertilization increased, indicating the canopy density were increasing. The high-N canopy curve (colored red; 3DCI = 0.471) contained less accumulative spatial variation than those with low (colored blue; 3DCI = 0.554) and medium-N (colored light green; 3DCI = 0.513) treatments (see cross-sections in Figure 6D) and hence possessed a smaller area beneath the curve. Trends in 3DCI scores across N treatments could also be used to differentiate varietal differences in canopy responses to N treatments. For example, increasing 3DCI indicated that the canopy became more variable in height, suggesting more structural responses to N applications. Similarly, if the index decreased sharply with the N increase, this indicated that the crop canopy became more uniform rapidly and likely much denser when the N application changed.

### Validation of the CropQuant-measured traits using ground truth data

**Figure 7:**
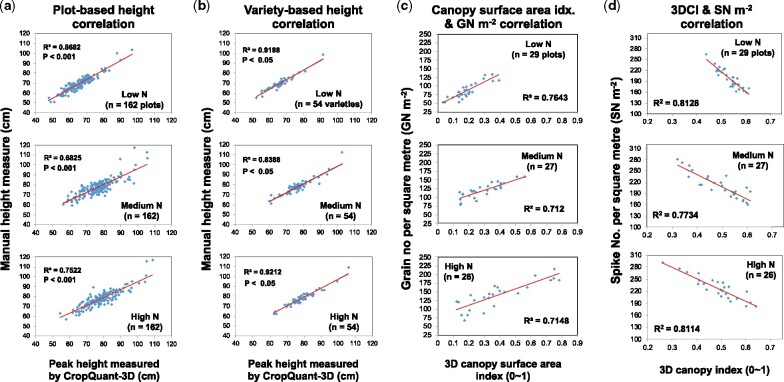
The square of the correlation coefficient (*R^2^*) calculated to evaluate correlations between height estimates, canopy surface area and 3DCI computed by CropQuant-3D and manual measurements in the 2019–2020 field trial, at three different levels of N fertilization; *P*-values computed through the linear regression analysis also reported. A, Plot-based correlation analysis of the peak height measured by CropQuant-3D and manual height measurements. B, Variety-based correlation analysis of the peak height measured by CropQuant-3D and manual height measurements. C, Correlation analysis of the 3D surface area index and the grain number per unit area (GN m^−2^) data. D, Correlation analysis between 3DCI and spike numbers per square meter (SN m^−2^). Plot means (A) and genotype means (B) are shown.

Height estimates derived from the CropQuant-3D output were validated by comparisons with manual height measurements taken at the same stage of crop development (grain filling) in the 2019/2020 trial. There was a strong correlation between the CropQuant-3D’s height scores and manual measurements for each level of N, using plot-based (the square of the correlation coefficient, *R*^2^, ranges from 0.69 and 0.87; *P*-value in linear regression analysis is less than 0.001; Figure 7A;  Supplemental Table S3) and variety-based means (*R*^2^ ranges from 0.84 and 0.92, *P *<* *0.05; Figure 7B;  Supplemental Table S4). Thus, the CropQuant-3D height scores based on the backpack LiDAR provides a viable alternative to manual height measurements, particularly for obtaining genotypic means. Interestingly, CropQuant-3D tended to underestimate the height for wheat varieties that are taller than 90 cm (some landraces were included). This is likely due to the way manual measurements were taken, which involved lifting and straightening curved or lodged plants to measure the distance from the soil surface to the tip of the ear along the vertical stem, whereas the LiDAR system measured the plants as they were naturally in the field. Furthermore, because only a limited number of plants were measured in each plot manually, compared with a whole plot scan conducted with the backpack LiDAR, there is a greater chance of plot-to-plot variability with the manual approach than with LiDAR, which integrates height measurements over a larger number of plants in a plot. Also, better variety-based correlation values might be due to height values for each variety have been averaged (three replicates per variety), reducing the height variance caused by treatments and small agronomic differences.

To verify the biological relevance of the 3D canopy surface area index, we have analyzed correlations with plot-level grain number (GN m^−2^) and grain yield (GW m^−2^) using data collected from the 11 selected varieties (*n* = 81 plots). Strong positive correlations between this LiDAR-derived trait and the yield components, with *R*^2^ ranging from 0.71 to 0.76 (*P *<* *0.001, Figure 7C;  Supplemental Table S5), suggest a mechanistic link between the canopy trait and grain formation underlying the correlation, indicating that the 3D surface area index can serve as a good predictor of dynamic varietal performance. Additionally, there was a strong negative correlation between 3DCI (designed to quantify canopy uniformity and density) and manually measured spike density (SN m^−2^) trait, with *R*^2^ ranging from 0.77 to 0.81 (*P *<* *0.001, Figure 7D;  Supplemental Table S6). Hence, it is likely that the 3DCI could also be used as a measure to quantify how SN m^−2^, a key yield component, responds to different N applications, but without the slow and laborious process of manually counting spikes in the field.

### A case study of classifying N responses for wheat

**Figure 8:**
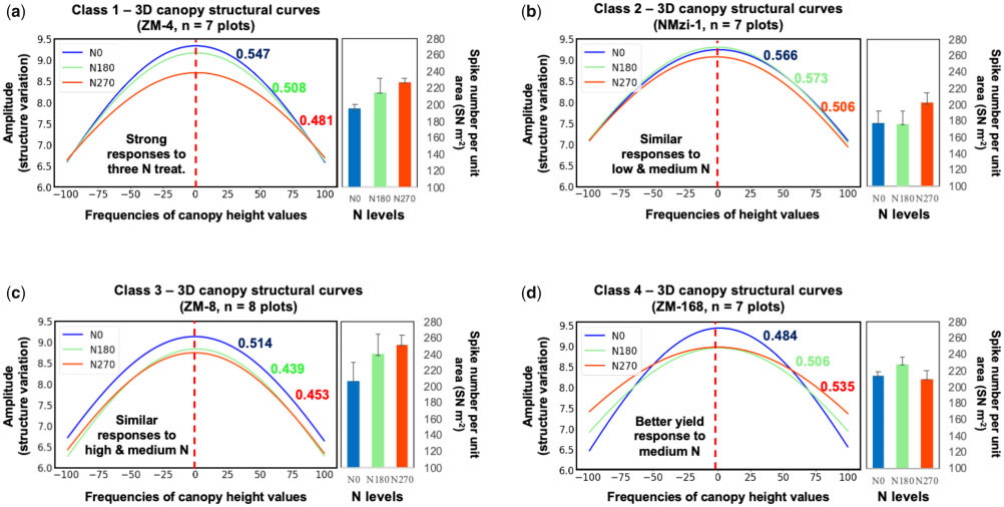
A case study of classifying wheat varieties’ N responses using the 3DCI and spike number per unit area for 11 varieties from the Zhenmai and Ningmai collections under three N application levels. Error bars used in the spike number per meter square (SN m^−2^) scores represent one standard error. A, The first N response class, showing canopy structural curves of ZM-4 and the associated spike number per meter square (SN m^−2^) scores under the three N treatments. Also in this class were varieties NMzi-1019, ZM-5, and ZM-1 (see Figure 6 for the explanation of the measure). B, The second N response class, showing canopy structural curves of NMzi-1 and the associated SN m^−2^ scores under the three N treatments. Also in this class were NMzi-1, ZM-10, and ZM-12. C, The third N response class, showing canopy structural curves of NM-26 and the associated SN m^−2^ scores under the three N treatments. Also in this class was ZM-8. D, The fourth N response class, showing canopy structural curves of ZM-168 and the associated SN m^−2^ scores under the three N treatments. Also in this class was line ZM-09196. Values shown in the corresponding color next to each curve in the plots are computed 3DCI values.

To effectively select crop varieties with an improved N response (e.g. high N use efficiency, NUE), it would be valuable to make use of proxy traits that are related to NUE under field conditions (Sylvester-Bradley and Kindred, 2009; Pask et al., 2012; Nguyen and Kant, 2018). The range of variables (e.g. 3D canopy surface area, canopy coverage, plot height and 3DCI) measured by CropQuant-3D were used jointly to describe canopy structural responses to three N treatments, which have enabled us to classify the N response of 11 selected wheat varieties (81 plots) into four classes (Figure 8). The example varieties were as follows:

1. Class 1—canopy structural curves differed across all three N levels. The patterns for ZM-4 could be clearly separated under the three N treatments (Figure 8A), indicating that this type of wheat variety had a strong structural response to varied N applications at the canopy. Both 3DCI (colored according to their associated N treatments) and the curvatures of the three canopy curves reduced steadily together with the increase of N, indicating that spike density and canopy uniformity were both rising in response to the escalation of N treatment. Also, the decrease of 3DCI corresponded with a continual increase of the SN m^−2^ reading. Other lines from the 11 varieties that can be categorized into Class 1 are NMzi-1019, ZM-5, and ZM-11 (Supplemental Figures S1).
2. Class 2—canopy structural curves were similar at low and medium N levels, but differed at high N. The patterns for NMzi-1 showed that the line had a good response to increased N, but only above the medium rate of N fertilization. Both 3DCI and SN m^−2^ suggested that low and medium N had similar effects on the variety (Figure 8B). The SN m^−2^ scores increased distinctly only under high N. Other lines that can be categorized into Class 2 are ZM-10 and ZM-12 (Supplemental Figures S2).
3. Class 3—canopy structural curves were similar at medium and high N levels. The patterns for NM-8 suggested that the variety had similar responses under medium and high N treatments, indicating the increasing N fertilization was not able to increase the line’s spike density beyond the medium rate of N fertilization (Figure 8C). The other line that can also be categorized into Class 3 is ZM-26 (Supplemental Figures S3).
4. Class 4—canopy structural curves decreased at high levels of N and showed the best response at medium N. Curvature patterns of ZM-168 indicated that the line had a similar canopy density at medium and high N treatments. The canopy uniformity was greater at the medium N level (3DCI = 0.506; Figure 8D) and the line’s spike density was the highest among the three N treatments. The other line that can be categorized into Class 4 is ZM-09196 (Supplemental Figures S4).

**Figure 9:**
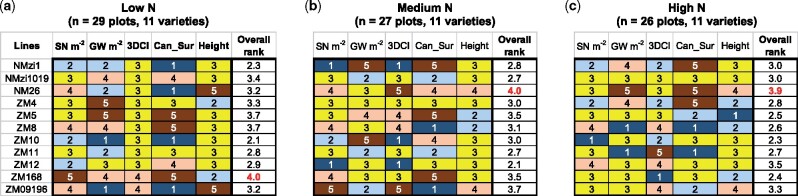
A performance matrix to evaluate NUE of wheat varieties using traits and measures for 11 wheat varieties from the Zhenmai and Ningmai collections under three N applications. A–C, A range of canopy measures (i.e. 3DCI and canopy surface area index), plot-level height, and key yield components, i.e. spike number per meter square (SN m^−2^) and grain number per meter square (GN m^−2^), combined to assess winter wheat varieties under three N treatments, with 15% over the trimmed mean colored dark orange, 7.5%–15% colored light orange, −7.5% to 7.5% colored yellow, −15% to −7.5% colored light blue, and −15% below the trimmed mean colored dark blue. Selected varieties were colored red, indicating they were ranked higher than the other varieties by the performance matrix.

After classifying N response patterns, we then combined 3DCI, crop height, canopy surface index area with the yield components, GN m^−2^ and SN m^−2^, to produce a performance matrix to understand crop responses to different N treatments in a compound manner. In the matrix, each variety was ranked based on the performance of these measures and traits. For example, by calculating the deviation of them based on the trimmed mean values (i.e. 15% over the trimmed mean colored dark orange and placed in rank order 5, the highest rank; 7.5%–15% colored light orange and placed in rank 4; −7.5% to 7.5% colored yellow and placed in rank 3; −15% to −7.5% colored light blue and placed in rank 2; and −15% below the trimmed mean colored dark blue and placed in rank 1, the lowest rank), we could select lines with a desired performance under the three N treatments using a ranking system. In particular, for crop height, both very short and very tall were ranked undesirable (i.e. placed in rank 1), whereas both GN m^−2^ and SN m^−2^ were given more weight (Langer and Liew, 1973) than other measures ($\mathrm{weights}=\left[0.25,0.25,0.2,0.1,0.2\right]$). Through the ranking system, we concluded that: (1) for the low N treatment, ZM-168 achieved a more balanced score in terms of grain production and structural variation (Figure 9A); for the medium N application, NM-26 ranked the highest (Figure 9B); and, for the high N, NM-26 was scored the highest (Figure 9C). Although this is only an initial attempt for selecting wheat varieties with desirable N responses using LiDAR-derived traits and key yield components, it is evident that the performance matrix could provide an objective approach to rank multiple wheat varieties. Further validation and field studies using the above approach are ongoing and will be reported separately.

## Discussion

Plant phenomics is an important area that helps provide valuable phenotypic information that is needed to fully exploit available genomic resources. For crop improvement programs, the focus is on multi-location and large-scale field phenotyping, yet there are a number of weaknesses with current solutions (Tardieu et al., 2017; Furbank et al., 2019; Pieruschka and Schurr, 2019), concerning: (1) mobility (a method can be straightforwardly used in multiple locations); (2) affordability (whether a purchase, operation, and maintenance of a system can be afforded by research groups with acceptable resources); (3) throughput (the number of plots, traits and fields that can be measured within a reasonable time frame, as well as the number of times to phenotype in a growing season); (4) accuracy (the information truly relates to the target attributes or biological functions of the plant); (5) resolution (if the method provides information at the level of detail required to test the biological hypothesis); and (6) scalability (the size of trials that can be phenotyped and the number of locations that can be covered).

In addition to data collection, another issue that limits the use of field phenotyping tools involve the ability to analyze big data acquired from the field (Kelly et al., 2016; Scharr et al., 2016; Cendrero-Mateo et al., 2017; Lobet, 2017). Although many open-source and proprietary software solutions have been developed (Butler et al., 2020; Roussel et al., 2020), their applications are normally limited to certain devices and for specific research questions, leading to matters such as software usability, data interoperability, and the generalisability (Carpenter et al., 2012; Roitsch et al., 2019). To address some of the above issues, we pioneered the integration of backpack LiDAR and an open-source software implementation to measure genotypic and N treatment differences in spatial features in wheat. Results from field experiments showed that structural measures (e.g. height, 3DCI, and canopy surface area) are highly correlated with key yield components such as SN m^−2^ and GN m^−2^, indicating the system could be used as a reliable research tool to classify the plant responses to different N treatments.

### The backpack LiDAR hardware

We have shown that the backpack LiDAR device introduced here is integrated and portable, enabling the collection of high-density 3D point clouds at the field and plot levels. Typically, these kinds of data would require LiDAR systems to be mounted on a gantry or vehicle platform, which are often not available, too costly, fixed in one location, or cannot reach fields with limited accessibility. To our knowledge, the backpack LiDAR system has not been used in field-based plant phenotyping previously. Hence, we developed a range of techniques to apply the device in wheat field experiments. Our field testing and development experience show that the backpack LiDAR possesses three notable features: (1) large-scale capability (up to 210 m effective scan range through our equipment), with an acceptable mapping speed (up to 1.2 ha/h); (2) portability (the ability to conduct multi-location phenotyping) with limited adjustments of hardware and software; (3) relatively small operation and maintenance costs due to its integration, ease-of-use and mobile features. Hence, backpack LiDAR appears to provide a more balanced solution to some current phenotyping challenges. Although backpack LiDAR, like most high-resolution LiDAR systems with high-end scanners, is still relatively expensive. However, costs should decrease and become more affordable as the technology matures (Su et al., 2020). Comparisons between backpack LiDAR devices and other approaches can be seen in the section below.

### CropQuant-3D software and trait analysis

Processing of 3D point cloud data collected by LiDAR systems for 3D trait analysis is still complicated and computationally demanding, indicating the necessity of reliable analytic solutions. Furthermore, for solutions that can be used by nonexperts and are widely accessible by the plant research community, the software should be user-friendly and openly available. Therefore, we developed the CropQuant-3D analysis software to routinely process large point cloud datasets. To help other researchers exploit our analysis algorithms integrated in the software, besides the GUI software, we also modularized the analysis tasks into individual procedures and then saved them with executable Python source code in Jupyter notebooks that can be executed on multiple operating systems. The algorithmic steps include pre-processing of 3D point clouds (Supplemental Methods S2), automated plot segmentation with optional experimental layout input, and plot-level crop height (see Supplemental Methods S3), 3D trait analysis of canopy structural features (3DCI, 2D canopy coverage, 3D canopy surface area), and biomass estimation such as 3DVI and 3DPI (see Supplemental Methods S4). Compared with the previous work (Ward et al., 2019; Hyyppä et al., 2020; Su et al., 2020), we have made progress in several areas for large-scale 3D trait analysis in plants:

1. Due to the huge volume of raw point cloud data collected, efficient data processing needs to be considered for both throughput and accuracy. Many existing methods require much computational time to pre-process point clouds. In our case, we have chosen to use a ground-level filter with parameters tailored for small-scale crop field, retaining only 3D points required by trait analysis. This approach noticeably reduced processing time. For example, for a 400 MB LiDAR file (over 15 million 3D points), only 100–120 s were required to normalize 3D points on an ordinary computer (intel i7 CPU and 16 MB memory; see profiling in the Material and Methods).
2. We analyzed plot-level 3D traits using 2D CHM, which retains sufficient spatial information in 2D pixels. This approach enabled us to employ computationally more efficient 2D-based algorithms such as edge detection, Hough transform, and adaptive thresholding to perform plot segmentation and trait analysis, reducing the computational complexity. Another key benefit for this 3D-to-2D transformation is that analysis regions could be controlled dynamically in any plot region. By calculating the texture entropy (Haralick et al., 1973), we could compute the weighted centroid of a plot and then define the sampling area according to experimental needs.
3. Since the density of the LiDAR-collected 3D points is likely to be imbalanced (e.g. the further away from the mapping route, the sparser the 3D points), it is necessary to interpolate the point clouds if the number of 3D points in a given plot is limited. From a range of interpolation algorithms, we have chosen the progressive TIN to build a TIN-based model and then iteratively densify 3D points in an, which helped us improve the quality of 3D trait analysis while retaining key 3D geometric features at the plot level.
4. It is technically difficult to describe 3D canopy structure quantitatively. The 2D Fourier transform method employed by CropQuant-3D opens a door to quantify spatial variances, spike density and uniformity at the canopy level by dividing frequency and amplitude of all height values across the plot. A similar idea but with a different approach can be found in measuring the canopy roughness of leafy trees in forest ecology (Antonarakis et al., 2010). Our approach was able to show that, through the canopy structural curve and 3DCI (Figure 6D), we could quantify the uniformity and density of wheat spikes in plots, which could be used to classify varieties according to different responses to N treatments and potentially other treatments. Meanwhile, the curvature of the canopy curves can also be employed to help distinguish the canopy density in relation to different N treatments and varieties.

There are many vision-based approaches developed to mine spatial and temporal features from point clouds for a range of biological questions, for example, identifying phenotypic differences at the organ level (Li et al., 2020a) and the extraction of single plants within a plot (Jin et al., 2021). Because our research aim was to enable large-scale field phenotyping for plot-level 3D trait analysis, we therefore did not consider plant-level 3D reconstruction and methods to analyze detailed features (e.g. plant-level marching cubes, leaf curvature estimation, and 3D skeletonization) in this work.

### Wheat varietal responses to different N fertilization levels

NUE in crops is generally low. Approximately 40% of the applied N can be utilized by cereal crops, with the bulk of the remainder leaching to groundwater or volatilizing to the atmosphere, causing increased agricultural costs and negative impacts on the environment (Raun and Johnson, 1999; Good et al., 2004). Breeding crop varieties with improved NUE should contribute to more sustainable cropping systems. To effectively select lines with heritable NUE-related proxy traits under different field conditions, it is technically difficult to screen many complex traits due to their dynamics and complexity (Good et al., 2004; Sylvester-Bradley and Kindred, 2009).

In the case study, we have explored a comprehensive procedure to quantify N responses of different wheat varieties based on phenotypic traits and key yield components. When the level of N changed, different varieties varied with their responses in terms of canopy structural features and key yield components. By combining key yield components and LiDAR-derived trait values, we identified four NUE types using the subset of 11 varieties: (1) grain yield responded well to increased N applications (Class 1); (2) only higher N was able to increase yield (Class 2); (3) medium and high N treatments led to similar grain production (Class 3); and (4) higher N led to a yield decrease (Class 4). We believe that the combined performance matrix demonstrated in the case study is likely to help establish an objective approach to identify wheat lines with superior N responses, which may lead to an effective selection improvement of NUE in wheat breeding programs in the future. Further work to link this selection approach with yield production and NUE at a large scale is ongoing.

### Applications of CropQuant-3D

The traits and measures here (e.g. height, coverage, canopy area, and 3DCI) do not just relate to N treatments, but they also closely connect with many aspects of genetic variation in crop performance. For example, crop height is an important factor in assessing risk to crop lodging, 3D canopy area and 2D ground coverage are good indicators for managing agricultural inputs to optimize canopy structure for radiation capture, photosynthetic output and transpirational water loss. It is also important to note that such traits are only apparent in the context of a population in plots, and most of these traits are difficult or impossible to convey by phenotyping individual plants in controlled environments. Canopy-level traits are affected by variety, soil characteristics and agronomic factors such as seed spacing and the application of plant growth regulators. The accuracy of plant models that attempt to simulate the effects of these factors and their interactions on crop performance could be improved by supplying them with traits presented here that were collected across a wide range of scenarios.

The 3D traits derived from LiDAR data such as 3DCI have many underlying component traits and spatial features. A better understanding of the bases of 3DCI would broaden its application for other crop improvement programs. For instance, height variances within a plot could be due to a variety of reasons: (1) a mixed population of plants with different genes controlling height, or that major height genes are not fixed, but still segregating in the population; (2) agronomic or environmental variability within the plot that is not related to genotypes; and (3) as 3DCI is affected by height as well as spike density, the analysis of 3D point clouds could likely pick up the differences in height of the mainstem, different tillers on each plant, and tillering response both to N treatment and genotype (Power and Alessi, 1978).

Another biological application of the CropQuant-3D system is for the discovery of robust quantitative trait loci for agronomic traits, which requires phenotypic data on large mapping populations across multiple field environments (Griffiths et al., 2012). The high-throughput capabilities of this combined system are well suited to this scale of research. A similar research approach has been reported in our recent work, SeedGerm (Colmer et al., 2020), which was applied to detect genetic differences in *Brassica napus* based on a range of seed germination traits. Although more work is needed, greater automation of phenotypic analysis and improvements in accuracy are likely to accelerate the genetic analysis of crop performance under varied treatments or environments.

Beyond existing 3D trait analysis, continuous phenotypic analysis in 3D of different crop species is likely to extend our understandings of the physiological bases of crop growth and development, for which the open-source nature of CropQuant-3D is likely to be valuable for the research community. There is an additional analytic power in examining longitudinal traits (time-series measures of traits that change as the crop develops and matures), which can describe the dynamic interactions between crop genotypes and N responses. By streamlining both the data acquisition and data analysis of field phenotyping with the backpack LiDAR and CropQuant-3D, it becomes possible to obtain measures at each key growth stage and at different test locations and environments, which was difficult to achieve with systems that are less portable and flexible in operation, with limited opportunity to expand or alter the use of the analysis software. With the approach introduced here, multi-environment 3D traits collected along a time series on large genotype collections could enable a deeper understanding of the genetic and physiological bases of efficient use of N for crop growth and development, as well as how these responses are modulated by the environment. Technically, other than some supervised machine learning algorithms, we have not embedded popular deep learning techniques into the analysis pipeline for 3D traits analysis. Continuous development will improve our work, opening 3D phenotypic analysis to nonexpert users and computational biologists who are willing to extend and jointly develop the platform. Overall, we believe that the combined backpack LiDAR and CropQuant-3D system could have a great potential to advance large-scale and multi-location field phenotyping, 3D phenotypic analysis, and genetic studies for both crop research and breeding applications.

### Issues associated with the backpack LiDAR and CropQuant-3D

Despite clear advantages, it is important to point out the limitations of the combined solution. LiDAR technology has been maturing very rapidly in recent years. The Robin backpack LiDAR used in this study is already being replaced by newer models with better accuracy, effective scan range, and a lower purchase price (the price of LiDAR devices has decreased over 30% since 2018; www.yole.fr/LiDAR_Market_Update_Livox_LiDAR.aspx). Although this type of LiDAR is more affordable than other large-scale systems, it is worth noting that, depending on the laser scanner integrated in a backpack LiDAR device, the equipment is still relatively expensive. We compared the costs of Robin with some representative backpack LiDAR systems, as well as other LiDAR-based mapping approaches (Supplemental Table S7; information regarding GPS and RTK accuracy can be found via the links in the References column). However, it is also notable that the integration and mobility features of backpack LiDAR possess a unique opportunity for the community to explore shared services or community-driven facilitates encouraged by EMPHASIS and AnaEE (Roy et al., 2017).

Additionally, our software was not designed to address many color- or spectral-related traits that are also important for crop performance. For example, senescence of the lower canopy due to differential N or water limitation. Adjustments to how the LiDAR is used and the associated analysis algorithms would be required to capture such traits in future work. However, similar issues can be applied to most of the LiDAR systems. Moreover, it was difficult to scan the lower part of the crop after the canopy closure, which could cause errors to estimate above-ground biomass with stems included. Also, due to field conditions such as wind movement of the plants, it is extremely challenging to generate a very high-resolution and high-precision 3D model to analyze an individual plant within the plot, even with high-end laser scanners or close-up 3D mapping modes. Alternative 3D point registration algorithms are therefore needed to deal with plant movement and reliable plant-level 3D modeling.

The CropQuant-3D system is capable of automating the segmentation of hundreds of plots for trait analysis, but the algorithm is likely to fail at the seedling development and tillering stages (GS10–29). This is because the early crop height map and the gaps between drilled plants are too big to ensure meaningful plot segmentation. However, as stems elongate and crop height increases (e.g. from the jointing stage onward, GS31), our system can perform reliable plot-level masking. Another technical issue that needs to be taken into consideration is the request for a user to select plot(s) to extract plot-level point clouds. Although plot-level point clouds are not required for the trait analysis reported here, a user is required to select one or multiple plots on the 2D CHM to extract associated point clouds, which can be laborious if point clouds from hundreds of plots need to be extracted. For this technical constraint, automated plot-level 3D points extraction is required and recent reports suggest they are within reach (Walter et al., 2019; Roussel et al., 2020; Jin et al., 2021).

Finally, because we have applied the 3D-to-2D analysis approach, some spatial information might be lost during the 3D-to-2D transformation, which could reduce the accuracy when the research interest is beneath the canopy region. For this loss of accuracy during the transformation, we have performed some testing using 3D point cloud files collected by other equipment such as drone and vehicle-mounted LiDAR (Figure 3, D and E) to carry out multi-scale point cloud processing. Although the preliminary is promising, further development and testing are still required to make the platform more compatible with these types of point cloud data. The next steps of the research also need to expand the application of CropQuant-3D to the analysis of different crop species so that the algorithms developed for wheat can be used for addressing similar biological problems in other crop species.

## Conclusion

The requirement of obtaining accurate and meaningful measures of the field phenotype at sufficient scale, throughput, cost, and multiple locations create a bottleneck in today’s crop research and breeding, which is preventing us from making full use of genomic resources for crop improvement programs. Backpack LiDAR has obvious advantages for large-scale field experiments and breeding trials. The device is easy to transport and use, overcoming the main limitations of fixed phenotyping platforms and can be used for multi-site data collection and at multiple time points. However, the ability to process and analyze large datasets with minimal time and standard computing power has limited the wide application of LiDAR-based phenotyping. To address this, we have developed CropQuant-3D, which processes large LiDAR-derived 3D point cloud data and consists of original algorithms packaged into user-friendly GUI software to output multiple 3D canopy traits (e.g. 3DCI) at the plot level. In a case study of 11 wheat varieties grown under three levels of N inputs, analysis results obtained by combining a backpack LiDAR and the CropQuant-3D software showed that wheat varieties could be classified into different N response groups according to a range of 3D traits that relate to spike density (SN m^−2^) and grain yield. This indicates that the combined solution could be a useful tool to make selections for NUE, and to dissect the physiological mechanisms and genetic regulation of NUE. Hence, we trust that the system presented here has a great potential to relieve some of the current bottlenecks in large-scale field phenotyping for crop research and breeding.

## Materials and methods

### Plant material and field experiments

In the first season (2018–2019), 105 Chinese winter wheat (*Triticum aestivum*) varieties were planted at the Zhenjiang Agricultural Technology Innovation Center (ZATIC, 31°57′N, 119°18′E, Jiangsu province, China), measured using CropQuant-3D and assessed for yield and N responses. A subset of 54 varieties (Supplemental Materials S9) was chosen out of the 105 lines for the 2019–2020 season. The selected 54 Chinese winter wheat varieties used in the field experiments were cultivated from the wheat plantation regions of the middle and lower reaches of the Yangtze river, which were shown previously to vary in performance and yield under different N treatments (Feng et al., 2008). A split-plot design was used, with three levels of N fertilization as main plots, containing three replicates of the 54 varieties as sub-plots (162 plots per N experiment). The overall size of the 2019–2020 field trial was 486 plots, covering ∼0.5 ha (Figure 1A). To explain the methods, data from 11 of the 54 varieties are shown.

### Crop management

Before sowing, soil samples (for 0–25 cm soil layer) were measured to ensure that available N content was suitable for N response studies (Supplemental Table S9). Following standard crop management guidelines (Godwin et al., 2003) and local practice, base fertilizer (P_2_O_5_ and K_2_O) was applied before drilling. Three levels of N fertilizer treatments were applied by hand (0, 180, and 270 kg N ha^−1^) in two splits: 50% at sowing and 50% at jointing (GS31). Crops were planted in 6 m^2^ plots (2 × 3 m), with 6 rows per plot at 15 cm spacing, with 30 cm gaps between plots (Figure 1A; trial plans in Supplemental Table S8). The planting density was 2.4 million plants per hectare. Plant growth regulator was not applied in the season so that stem elongation could respond unimpeded to different levels of N treatments.

### Manual measurement

To collect reliable ground truth data for validating and improving CropQuant-3D’s analysis algorithm, a team of five field workers performed the manual scoring. They conducted a range of manual measures at key growth stages (from heading, GS51–59, to grain filling, GS71–89), including plant height, growth stage scoring, and key yield components such as spike number density (SN m^−2^), spikes per plant, grain number per unit area (GN m^−2^), and thousand grain weight. For example, manual plant height measures of five typical plants per plot were conducted on May 11, 18, and 26, 2020, from which the scores on May 18 (2 d after the LiDAR mapping, May 16, 2020) were used for correlation studies in this work. As there were variances in height across the plot, three one meter-square regions were selected to represent height variances within a plot. Then, all plants in the region were measured and the average height value was recorded as the plot height value. When measuring the plant height, the distance from the ground to the top of the ear was measured with a steel ruler. We took steps to standardize manual measurements: (1) cross-scoring the same traits with different field workers; (2) cross-validating scores across experiments using historic data; and (3) using trimmed mean to remove outlier values before calculating the average of ground truth. At maturity, the yield was measured in a 1-m^2^ quadrat centered in the plot, from which ears were removed with a sickle. Threshing was carried out with a plot thresher; any grain that passed through the thresher was manually recovered from the sieved straw.

### The backpack LiDAR system

The backpack LiDAR (Robin Precision, 3DLasermapping; purchased by GeoSLAM, Nottingham, UK) integrates a laser scanner (RIEGL VUX-1) and three mapping settings, employing accurate GPS-tagged navigation, and was used in conjunction with a RTK base station for precise positioning. The system is lightweight (around 10 kg) and comprises a high-performance laser mapping system (360° scanning angle with an effective scan range of 3200 m; further detail in Supplemental Methods S5). Measurements focused on the key growth stages (Zadocks et al., 1974), from heading (GS51–59) to grain filling (GS71–89) when canopy structural features were largely established. Standard pre-processing software packages were bundled with the device. To capture the peak height for the selected wheat varieties, the trial was mapped from April to May 2020. In our preliminary work, similar 3D field mapping was conducted in paddy rice trials at the Tuqiao crop breeding and cultivation centre (Jiangsu China) and at the Chinese Academy of Sciences’ Songjiang crop research center (Shanghai China, Supplemental Figures S5 and S6). CropQuant-3D is not bundled with Robin and can be used to analyze point cloud files generated by other sources.

### GUI-based software development

To develop the GUI-based analysis software for CropQuant-3D, we utilized PyQt5, a comprehensive set of Python bindings for the Qt v5 library (pypi.org/project/PyQt5/), which was developed using C++ and is cross-platform for modern desktop (e.g. Windows and Mac OS) and mobile (e.g. Android and iOS) systems. The GUI software we developed follows a traditional desktop-based user interface development, which can be easily modified to operate in a web browser such as Google Chrome. Anaconda Python release (docs.continuum.io/anaconda/install/windows) was employed as our integrated development environment, through which third-party libraries required for the software implementation, testing and packaging were managed by multiple virtual environments installed into the conda directory (Virtanen et al., 2020). Algorithms (in Jupyter notebooks), GUI software (in EXE format), Python-based source code and testing files (in LAS format) are freely available.

### Software implementation

To implement *Step 1* (denoising) in the analysis pipeline introduced in the Results section, we first used the file.File function in the laspy library to read the input file, followed by the spatial.cKDTree function in the Scipy library to index the 3D coordinates of all the points in the LAS file. Then, we applied the filtering criteria (i.e. *avg.* + *k* × *std.*) to index outliers in the point clouds and saved the denoised point cloud data using the function file.File (in LAS format).

For *Step 2* (filtering) in the pipeline, we developed three approaches to process point cloud files generated through different approaches: (1) for the backpack LiDAR mapping, we used the function lidar_ground_point_filter in the WhiteboxTools library to filter the point cloud; (2) for UAV-SfM generated pint cloud files, we employed the function do_filtering in the CSF library to separate ground-level 3D points from above-ground points; (3) for the gantry-mounted LiDAR files, because the 3D points have already been filtered, we could use the files directly.

For *Step 3* (the generation of CHM) in the pipeline, we also developed three approaches to process different types of point cloud files: (1) for the backpack LiDAR generated files, we applied the function lidar_tin_gridding in the WhiteboxTools library to output CHMs with the resolution parameter set as 1 cm/pixel; (2) for UAV-SfM files, we used the lidar_tin_gridding function to output digital earth model (DEM) and DSM, followed by the clip_raster_to_polygon function to rectify the DSM and DEM’s resolution using the shapefile (the .shp file collected by RTK), resulting in an CHM imaging produced through subtracting the DEM from the DSM; (3) for the gantry LiDAR files, the lidar_nearest_neighbour_gridding function was used to produce the CHM image.

For *Step 4* (the definition of ROI) in the pipeline, we used the function read_csv in the pandas library to read the geo-coordinates of the point cloud files, followed by the open function in the rasterio library to open the CHM and convert the geo-coordinates to pixel coordinates so that 3D point clouds could be analyzed in 2D. The function getPerspectiveTransform in the OpenCV library was employed to obtain the perspective transformation matrix together with the warpPerspective function in OpenCV to define the ROI in the 2D CHM. Finally, the io.imsave in the scikit-image library was used to save the aligned 2D CHM within ROI.

For *Step 5* (plot segmentation) in the pipeline, the optional input parameters such as the number of rows and columns could be used to generate horizontal and vertical baselines to assist the plot segmentation. Using the threshold_sauvola and threshold_local functions in scikit-image, we could obtain the threshold mask of the CHM image. Then, we applied the sobel function in scikit-image to detect edges in the CHM, followed by the hough_line function to fit vertical and horizontal lines, separately. By merging the detected lines and baselines, we could generate the final mask representing the plot boundaries in the field.

For plot-based 2D/3D trait analysis, we mainly used the regionprops function in scikit-image to calculate phenotypic traits in each plot. The plot-level 3D canopy traits were based on the clip_lidar_to_polygon function in WhiteboxTools to crop plot-level point clouds. The source code produced from the above software implementation can be seen in Supplemental Methods S2–S4, as well as from our GitHub repository.

### Software profiling

We profiled the GUI software using a range of testing point cloud files (in LAS format, available on our GitHub repository), which were acquired by the backpack LiDAR (403 MB; 15,090,552 points), UAV SfM generated point clouds (596 MB; 18,372,420 points), and gantry LiDAR (FieldScan^TM^, 1.42 GB; 58,446,207 points). Three Windows laptop computers with different hardware configurations were used for the software profiling: (1) Intel Core i5 with 8GB memory (budget laptop); (2) Intel Core i7 processor and 24GB memory (middle-end laptop); and (3) Intel Core i9 with 32 GB memory (high-end laptop). As the CropQuant-3D software did not support GPU acceleration, both CPU and memory influenced the processing performance of CropQuant-3D. By averaging the computational time (using the time module in Python) used by the three computers, we provided details on the processing time using the three types of testing files at each step (Supplemental Table S10).

## Availability and requirements

Project name: 3D field phenotyping for wheat using backpack LiDAR and CropQuant-3D

Project home page: https://github.com/The-Zhou-Lab/LiDAR

Source code: https://github.com/The-Zhou-Lab/LiDAR/releases/tag/V2.0

GUI software: https://github.com/The-Zhou-Lab/LiDAR/releases/tag/V2.0

Programming language: Python 3.7

Requirements: Laspy (1.7.0), Whitebox (1.3.0), GDAL (3.1.4), Rasterio (1.1.8), Open3D (0.11.2), Mayavi (4.7.2), Scikit-Image (0.17.2), OpenCV-Python (4.4.0.46), Pandas (1.1.5), Numpy(1.19.4), Matplotlib(3.3.3), and Scipy (1.5.3).

License: The MIT License for open-source initiative (https://opensource.org/licenses/MIT).

## Availability of supporting data

The datasets supporting the results presented here are available at https://github.com/The-Zhou-Lab/LiDAR/releases/tag/V2.0. Source code and other supporting data are openly available on request.

## Supplemental Data

The following materials are available in the online version of this article.

**Supplemental Figure S1**. Canopy structural curves of four wheat varieties (*n* = 31 plots), ZM-4, NMzi-1019, ZM-5, and ZM-11, which were classified into Class One due to their similar N-response patterns.

**Supplemental Figure S2**. Canopy structural curves of three wheat varieties (*n* = 23 plots), NMzi-1, ZM-10, and ZM-12, which were classified into Class Two due to similar N-response patterns.

**Supplemental Figure S3**. Canopy structural curves of two wheat varieties (*n* = 15 plots), NM-26 and ZM-8, which were classified into Class Three due to similar N-response patterns.

**Supplemental Figure S4**. Canopy structural curves of two wheat varieties (*n* = 12 plots), ZM-168 and ZM-09196, which were classified into Class Four due to similar N-response patterns.

**Supplemental Figure S5**. The backpack LiDAR used in three experimental fields at Tuqiao field center (Jiangsu China), examining 1,458 1 m^2^ rice plots under two levels of N treatments (i.e. 180 and 270 kg N ha^−1^).

**Supplemental Figure S6**. The backpack LiDAR used in an experimental field at Songjiang crop research center (Shanghai China), examining 261 1 m^2^ rice landraces.

**Supplemental Table S1**. CropQuant-3D-measured crop height values for 486 6-meter plots (54 wheat varieties with three replicates) under three N treatments (0, 180 and 270 kg N ha^−1^).

**Supplemental Table S2**. CropQuant-3D’s traits analyses of 81 six-metre plots of ZM & NM varieties under three N treatments (0, 180, and 270 kg N ha^−1^), generated by the GUI-based software.

**Supplemental Table S3**. Plot-based correlation performance metrics evaluate CropQuant-3D-measured height values using manual height measurement under three N treatments (0, 180 and 270 kg N ha^−1^).

**Supplemental Table S4**. Variety-based correlation performance metrics evaluate CropQuant-3D-measured height values using manual height measurement under three N treatments.

**Supplemental Table S5**. Correlation performance metrics evaluate CropQuant-3D-measured canopy surface area trait using manual grain number per unit area (GN m^−2^) scores under three N treatments.

**Supplemental Table S6**. Correlation performance metrics evaluate CropQuant-3D-measured 3DCI trait using manual spike number per unit area (SN m^−2^) scores under three N treatments.

**Supplemental Table S7**. Cost comparison between backpack LiDAR devices, UAV airborne LiDAR, and the handheld laser scanning system, with brief technical specifications.

**Supplemental Table S8**. Three split fields used to study three replicates of 54 wheat varieties under three levels of N fertilizer treatments (i.e. 0, 180, and 270 kg N ha^−1^). Crops were planted in 6-squaremeter plots (2 × 3 m), 486 plots in total.

**Supplemental Table S9**. Soil nutrient (0–25 cm soil layer) content measured before drilling in the 2019–2020 season.

**Supplemental Table S10**. Processing time for three types of point cloud files, collected by backpack LiDAR, UAV SfM photogrammetry, and gantry-based LiDAR, at each analysis step.

**Supplemental Methods S1**. A step-by-step user guide of how to use the GUI-based CropQuant-3D software to perform 3D trait analysis of point cloud datasets collected by backpack LiDAR, UAV SfM photogrammetry and gantry-based LiDAR.

**Supplemental Methods S2**. Python-based code fragments for pre-processing LiDAR-collected point cloud datasets.

**Supplemental Methods S3**. Python-based code fragments for automatically segmenting plots using the pre-processed LiDAR point cloud datasets.

**Supplemental Methods S4**. Python-based code fragments for performing plot-based 3D trait analysis, including the measurement of canopy structural variation such as 3DCI.

**Supplemental Methods S5**. The introduction of the backpack LiDAR device, Robin Precision^TM^, used in this study.

**Supplemental Movie S1**. An instructional video showing how to use CropQuant-3D in action.

## Supplementary Material

kiab324_Supplementary_Data

## Notes

J.Z. and E.O. wrote the manuscript with inputs from Y.L.Z., S.C.J., Q.Z., and J.C. J.Z., Y.L.Z., and G.S. designed the 3D phenotypic analysis algorithms. Y.L.Z., G.S., and Jie Z. implemented the software under J.Z.’s supervision. G.H.D. and M.X.W. performed the field wheat experiments under J.Z. and Y.F.D.’s supervision. S.C.J. provided LiDAR expertise. J.Z., Y.L.Z., and Jie Z. optimized the algorithms and tested the software. J.Z., Q.Z., and J.C. performed the data analysis. All authors read and approved the final manuscript.

The author responsible for distribution of materials integral to the findings presented in this article in accordance with the policy described in the Instructions for Authors (https://academic.oup.com/plphys/pages/general-instructions) is: Ji Zhou (Ji.Zhou@NJAU.edu.cn or Ji.Zhou@NIAB.com).
