# Supplementary material for: Large-scale field phenotyping using backpack LiDAR and CropQuant-3D to measure structural variation in wheat
Source: Plant Physiol. 2021 Jul 16;187(2):716–38. doi: 10.1093/plphys/kiab324 (PMC8491082; doi:10.1093/plphys/kiab324)
Supplement: kiab324_Supplementary_Data [file kiab324_supplementary_data.zip › pp.00744.2021-s02.pdf]

Supplemental Data

Title: Large-scale field phenotyping using backpack LiDAR and CropQuant-3D to measure structural responses in wheat

Authors: Yulei Zhu[1], Gang Sun[1], Guohui Ding[1], Jie Zhou[1], Mingxing Wen[2], Shichao Jin[1], Qiang Zhao[3], Joshua Colmer[4], Yanfeng Ding[1], Eric S Ober[5], Ji Zhou[1,5*]

Corresponding: Ji.Zhou@njau.edu.cn, Ji.Zhou@NIAB.com

Table of Contents

SUPPLEMENTAL DATA 1

SUPPLEMENTAL FIGURES 2

SUPPLEMENTAL TABLES 5

SUPPLEMENTAL METHODS S1 18

SUPPLEMENTAL METHODS S2 26

SUPPLEMENTAL METHODS S3 31

SUPPLEMENTAL METHODS S4 40

SUPPLEMENTAL METHODS S5 45

SUPPLEMENTAL MOVIE LEGEND 47

Supplemental Figures

The following figures (**Figs. S1-S4**) present the 81 canopy structural curves (one curve per N application per variety) produced by CropQuant-3D's 3D canopy trait analysis. Based on density and uniformity at the canopy level, four types of canopy responses patterns were concluded using 11 wheat varieties (with three replicates) selected for the 2019-2020 NUE case study.

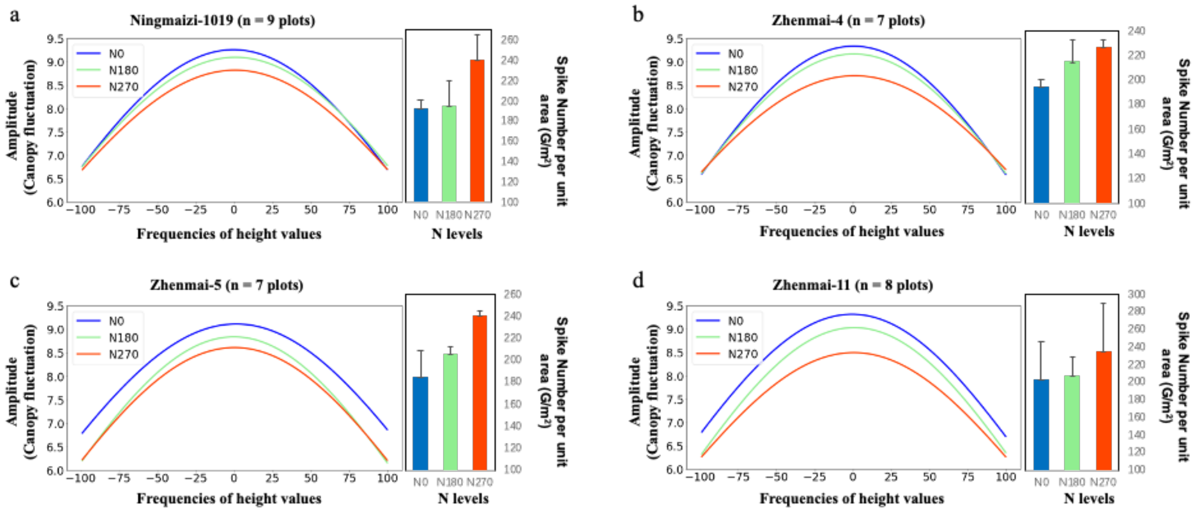

Figure S1. Canopy structural curves of four wheat varieties (n = 31 plots), ZM-4, NMzi-1019, ZM-5 and ZM-11, which were classified into Class One due to similar N-response patterns.

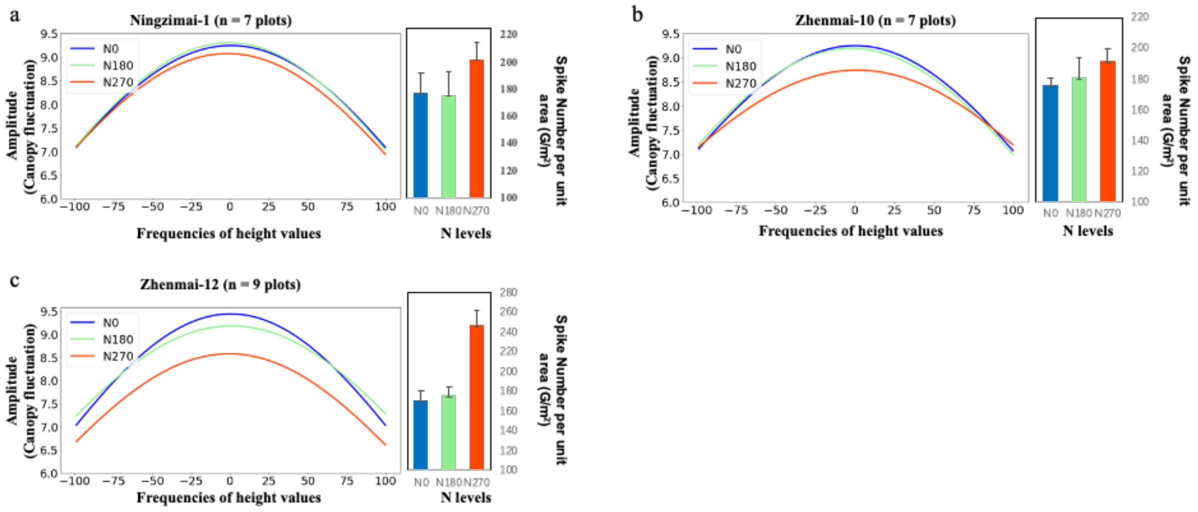

Figure S2. Canopy structural curves of three wheat varieties (n = 23 plots), NMzi-1, ZM-10 and ZM-12, which were classified into Class Two due to their similar N-response patterns.

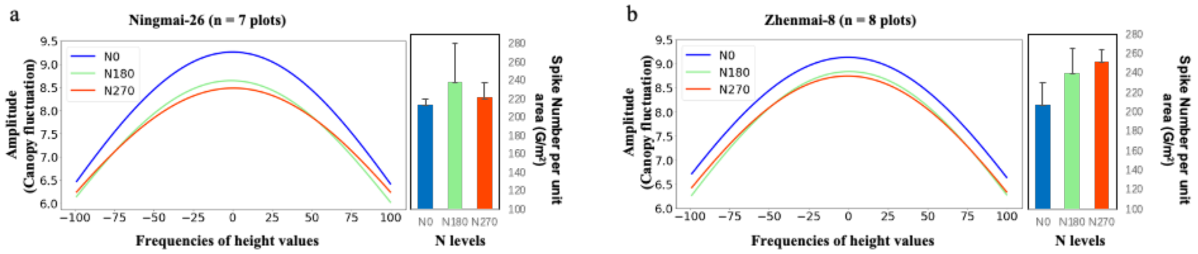

Figure S3. Canopy structural curves of two wheat varieties (n = 15 plots), NM-26 and ZM-8, which were classified into Class Three due to similar N-response patterns.

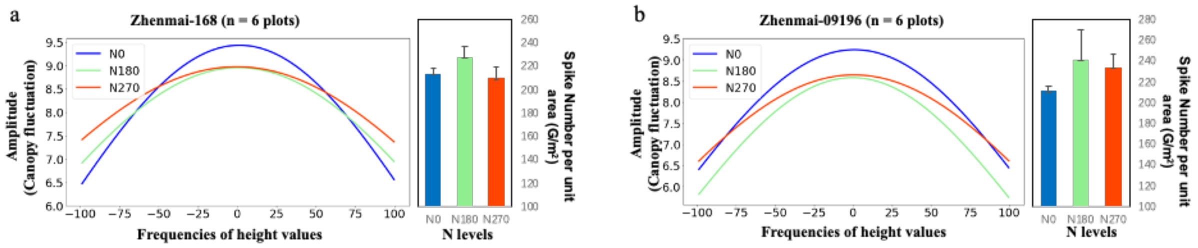

Figure S4. Canopy structural curves of two wheat varieties (n = 12 plots), ZM-168 and ZM-09196, which were classified into Class Four due to similar N-response patterns.

We have also utilised the backpack LiDAR and CropQuant-3D to collect and analyse rice crops in two trial sites. For the rice field phenotyping at Tuqiao field center (Jiangsu China), we have used the system to study rice varieties under two levels of N treatments (i.e. 180 and 270 kg N ha^{-1}, **Fig. S5)**. Crops were planted in 1 m^2 plots and 1,458 plots in total were mapped.

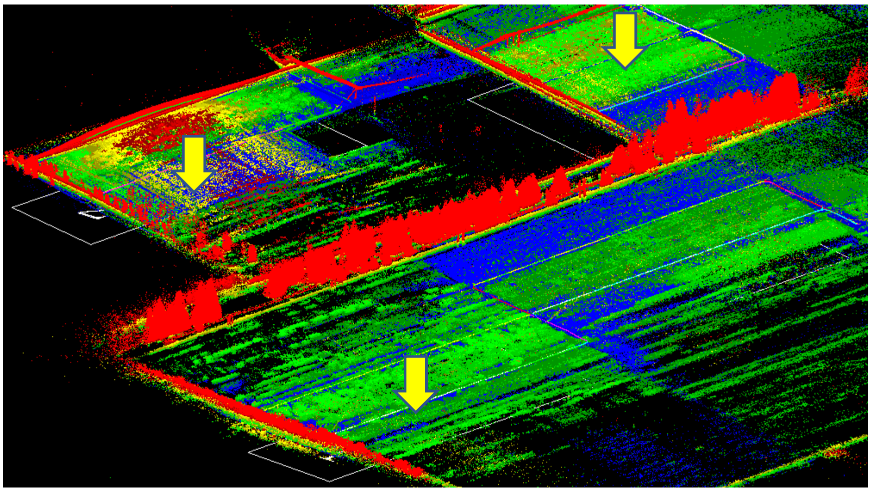

Figure S5. The backpack LiDAR used in three experimental fields at Tuqiao field center (Jiangsu China), examining 1,458 1 m^2 rice plots under two levels of N treatments (i.e. 180 and 270 kg N ha^{-1}**)**.

We used the combined solution to study paddy rice to assess genetic variation at the Chinese Academy of Sciences (CAS) Songjiang crop research center (Shanghai China, **Fig. S6)**. Crops were planted in 1 m^2 plots and 261 landraces were mapped to differentiate genetic variation.

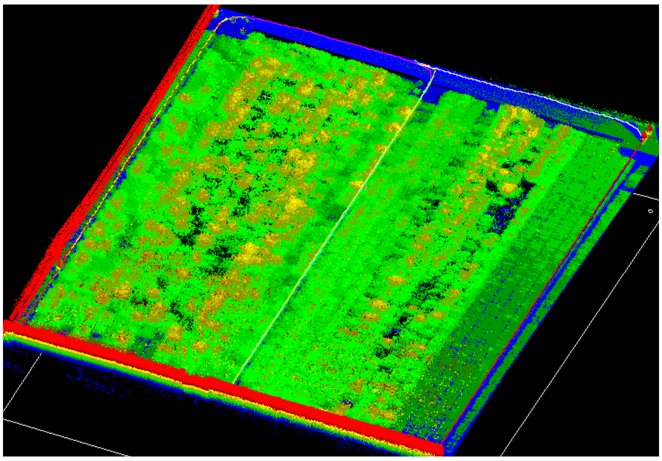

Figure S6. The backpack LiDAR used in an experimental field at Songjiang crop research center (Shanghai China), examining 261 1 m^2 paddy rice varieties.

Supplemental Tables

Table S1. CropQuant-3D-measured crop height values for 486 six-metre wheat plots (54 varieties with three replicates) under three N treatments (0, 180 and 270 kg N ha^{-1}).

Plot_id	N_level	Heigth_lidar	Height_manual	Plot_id	N_level	Heigth_lidar	Height_manual	Plot_id	N_level	Heigth_lidar	Height_manual
plot_1	N0	57.0	64.4	plot_163	N180	79.8	78.6	plot_325	N270	73.3	73.3
plot_2	N0	67.6	72.3	plot_164	N180	77.6	84.1	plot_326	N270	82.1	81.3
plot_3	N0	59.6	70.3	plot_165	N180	87.8	84.2	plot_327	N270	81.6	80.2
plot_4	N0	67.0	73.1	plot_166	N180	76.9	79.1	plot_328	N270	85.2	77.1
plot_5	N0	73.5	73.2	plot_167	N180	63.6	65.0	plot_329	N270	78.8	75.9
plot_6	N0	67.6	73.9	plot_168	N180	80.4	80.0	plot_330	N270	64.9	65.1
plot_7	N0	72.8	72.7	plot_169	N180	66.7	79.9	plot_331	N270	77.1	76.5
plot_8	N0	74.2	74.2	plot_170	N180	88.5	88.6	plot_332	N270	80.5	85.4
plot_9	N0	67.1	70.7	plot_171	N180	70.5	78.7	plot_333	N270	105.3	115.8
plot_10	N0	68.5	75.0	plot_172	N180	72.5	82.3	plot_334	N270	79.6	77.7
plot_11	N0	70.7	72.1	plot_173	N180	64.3	61.7	plot_335	N270	77.5	74.7
plot_12	N0	60.4	65.2	plot_174	N180	75.8	79.1	plot_336	N270	85.0	82.0
plot_13	N0	65.8	66.9	plot_175	N180	65.8	73.6	plot_337	N270	72.8	67.9
plot_14	N0	67.0	65.6	plot_176	N180	77.5	82.4	plot_338	N270	101.0	93.6
plot_15	N0	72.1	73.5	plot_177	N180	81.9	79.6	plot_339	N270	83.1	83.7
plot_16	N0	64.9	72.2	plot_178	N180	75.9	81.2	plot_340	N270	67.0	68.5
plot_17	N0	68.2	70.7	plot_179	N180	105.1	112.8	plot_341	N270	80.1	81.6
plot_18	N0	60.6	66.5	plot_180	N180	76.9	82.4	plot_342	N270	74.0	76.4
plot_19	N0	61.9	66.4	plot_181	N180	78.0	74.3	plot_343	N270	87.7	84.7
plot_20	N0	67.2	70.1	plot_182	N180	77.7	75.5	plot_344	N270	80.6	71.8
plot_21	N0	71.3	77.6	plot_183	N180	83.3	82.3	plot_345	N270	92.9	92.1
plot_22	N0	70.2	74.4	plot_184	N180	83.9	89.7	plot_346	N270	77.9	72.1
plot_23	N0	65.9	68.3	plot_185	N180	71.1	71.2	plot_347	N270	87.2	77.6
plot_24	N0	56.8	62.6	plot_186	N180	78.7	75.3	plot_348	N270	80.3	75.5
plot_25	N0	61.0	64.8	plot_187	N180	77.4	77.1	plot_349	N270	77.9	77.2
plot_26	N0	70.4	74.0	plot_188	N180	82.1	76.4	plot_350	N270	67.0	65.5
plot_27	N0	65.6	68.8	plot_189	N180	78.5	83.3	plot_351	N270	78.2	76.2
plot_28	N0	61.0	64.5	plot_190	N180	63.3	68.2	plot_352	N270	78.3	79.0
plot_29	N0	63.8	68.7	plot_191	N180	72.5	67.1	plot_353	N270	77.1	74.7
plot_30	N0	61.1	67.9	plot_192	N180	97.8	95.6	plot_354	N270	87.3	81.6
plot_31	N0	65.3	68.3	plot_193	N180	81.5	83.4	plot_355	N270	78.2	72.3
plot_32	N0	65.3	71.3	plot_194	N180	83.4	75.3	plot_356	N270	86.7	84.1
plot_33	N0	70.3	75.5	plot_195	N180	85.7	87.3	plot_357	N270	75.3	76.4
plot_34	N0	73.4	74.3	plot_196	N180	81.4	85.0	plot_358	N270	76.2	76.8
plot_35	N0	70.9	72.6	plot_197	N180	75.3	77.0	plot_359	N270	91.6	88.7
plot_36	N0	70.4	74.8	plot_198	N180	76.9	72.1	plot_360	N270	88.4	84.1
plot_37	N0	62.6	61.3	plot_199	N180	92.3	83.6	plot_361	N270	69.6	66.6
plot_38	N0	71.1	74.4	plot_200	N180	74.0	71.9	plot_362	N270	94.0	92.8
plot_39	N0	58.0	67.0	plot_201	N180	76.6	79.0	plot_363	N270	80.7	76.2
plot_40	N0	57.9	63.8	plot_202	N180	89.4	106.0	plot_364	N270	84.1	88.0
plot_41	N0	66.8	68.4	plot_203	N180	77.0	79.4	plot_365	N270	74.8	73.2
plot_42	N0	68.9	69.5	plot_204	N180	80.7	77.3	plot_366	N270	87.1	80.9
plot_43	N0	72.4	74.2	plot_205	N180	79.0	76.8	plot_367	N270	71.4	71.0
plot_44	N0	66.2	69.5	plot_206	N180	80.2	81.4	plot_368	N270	78.8	76.7
plot_45	N0	65.5	73.5	plot_207	N180	82.6	81.9	plot_369	N270	71.0	68.5
plot_46	N0	57.7	60.7	plot_208	N180	74.3	74.5	plot_370	N270	75.9	78.6
plot_47	N0	72.7	76.3	plot_209	N180	76.4	76.7	plot_371	N270	83.9	85.2
plot_48	N0	69.2	74.5	plot_210	N180	75.0	80.3	plot_372	N270	90.1	84.8
plot_49	N0	57.2	62.2	plot_211	N180	73.2	73.7	plot_373	N270	69.6	72.5
plot_50	N0	80.1	81.6	plot_212	N180	78.4	73.8	plot_374	N270	82.5	78.9
plot_51	N0	52.8	57.6	plot_213	N180	65.5	65.3	plot_375	N270	63.9	61.1
plot_52	N0	69.4	75.7	plot_214	N180	90.1	90.7	plot_376	N270	96.3	88.5
plot_53	N0	71.9	75.3	plot_215	N180	72.4	70.7	plot_377	N270	86.3	80.0
plot_54	N0	87.9	99.8	plot_216	N180	77.1	70.3	plot_378	N270	83.6	78.1
plot_55	N0	55.8	60.5	plot_217	N180	60.9	64.4	plot_379	N270	68.9	71.4
plot_56	N0	66.5	73.0	plot_218	N180	81.9	79.7	plot_380	N270	87.7	86.9
plot_57	N0	72.1	76.3	plot_219	N180	78.6	75.7	plot_381	N270	79.0	75.9
plot_58	N0	57.6	61.5	plot_220	N180	77.4	73.5	plot_382	N270	75.9	79.4
plot_59	N0	81.2	82.4	plot_221	N180	80.5	69.2	plot_383	N270	95.0	86.6
plot_60	N0	62.1	67.3	plot_222	N180	80.1	77.1	plot_384	N270	82.5	75.8
plot_61	N0	66.9	64.8	plot_223	N180	75.8	77.8	plot_385	N270	67.3	69.3
plot_62	N0	67.5	72.4	plot_224	N180	78.7	77.8	plot_386	N270	79.6	77.3

plot_63	N0	69.5	71.7	plot_225	N180	65.8	67.5	plot_387	N270	77.9	79.3
plot_64	N0	71.8	75.8	plot_226	N180	77.5	74.9	plot_388	N270	80.6	76.0
plot_65	N0	69.5	72.2	plot_227	N180	79.5	72.9	plot_389	N270	67.9	65.1
plot_66	N0	62.9	67.2	plot_228	N180	74.8	75.8	plot_390	N270	68.9	69.4
plot_67	N0	66.2	69.4	plot_229	N180	68.4	70.8	plot_391	N270	65.5	61.2
plot_68	N0	81.0	84.8	plot_230	N180	79.9	76.1	plot_392	N270	80.3	76.2
plot_69	N0	96.7	103.4	plot_231	N180	75.1	72.5	plot_393	N270	89.4	87.5
plot_70	N0	67.9	70.2	plot_232	N180	79.5	76.3	plot_394	N270	80.4	83.6
plot_71	N0	74.4	78.3	plot_233	N180	78.4	77.1	plot_395	N270	81.8	79.5
plot_72	N0	57.9	67.5	plot_234	N180	70.2	75.0	plot_396	N270	86.6	80.0
plot_73	N0	76.7	74.7	plot_235	N180	58.8	69.0	plot_397	N270	62.0	66.7
plot_74	N0	65.4	68.7	plot_236	N180	75.3	76.2	plot_398	N270	68.0	75.1
plot_75	N0	69.2	77.0	plot_237	N180	94.0	85.9	plot_399	N270	82.8	73.4
plot_76	N0	68.9	72.7	plot_238	N180	84.9	82.1	plot_400	N270	67.1	74.2
plot_77	N0	70.5	78.2	plot_239	N180	94.8	88.1	plot_401	N270	78.8	79.7
plot_78	N0	65.1	66.3	plot_240	N180	72.0	76.0	plot_402	N270	73.3	76.1
plot_79	N0	62.7	64.3	plot_241	N180	61.2	59.6	plot_403	N270	61.6	63.4
plot_80	N0	55.5	57.0	plot_242	N180	105.5	107.0	plot_404	N270	85.0	84.6
plot_81	N0	76.5	78.3	plot_243	N180	75.3	76.2	plot_405	N270	79.6	81.5
plot_82	N0	71.1	77.5	plot_244	N180	77.1	78.1	plot_406	N270	73.8	74.5
plot_83	N0	76.4	76.6	plot_245	N180	75.8	76.1	plot_407	N270	104.9	101.6
plot_84	N0	67.6	73.7	plot_246	N180	68.1	69.7	plot_408	N270	81.3	76.7
plot_85	N0	67.2	66.1	plot_247	N180	63.3	71.5	plot_409	N270	78.7	77.7
plot_86	N0	69.0	74.0	plot_248	N180	73.9	71.7	plot_410	N270	79.9	79.9
plot_87	N0	64.7	67.3	plot_249	N180	73.6	74.5	plot_411	N270	83.5	84.4
plot_88	N0	71.3	74.4	plot_250	N180	82.6	81.9	plot_412	N270	87.6	85.8
plot_89	N0	69.4	74.7	plot_251	N180	76.6	73.4	plot_413	N270	79.5	76.8
plot_90	N0	55.2	64.5	plot_252	N180	63.4	65.7	plot_414	N270	79.8	79.0
plot_91	N0	59.0	61.7	plot_253	N180	65.6	73.4	plot_415	N270	104.2	94.5
plot_92	N0	62.6	66.8	plot_254	N180	80.1	78.2	plot_416	N270	77.4	82.1
plot_93	N0	67.3	66.5	plot_255	N180	83.3	80.1	plot_417	N270	84.7	76.0
plot_94	N0	61.7	68.6	plot_256	N180	89.9	92.1	plot_418	N270	79.1	79.5
plot_95	N0	70.3	72.9	plot_257	N180	98.6	100.2	plot_419	N270	81.3	79.1
plot_96	N0	62.5	68.2	plot_258	N180	70.9	73.7	plot_420	N270	82.0	83.7
plot_97	N0	54.8	57.5	plot_259	N180	82.5	85.6	plot_421	N270	74.0	89.5
plot_98	N0	70.4	72.9	plot_260	N180	73.2	77.6	plot_422	N270	77.0	72.9
plot_99	N0	67.0	66.8	plot_261	N180	60.9	72.9	plot_423	N270	78.1	81.0
plot_100	N0	68.5	74.1	plot_262	N180	66.9	73.9	plot_424	N270	73.9	75.1
plot_101	N0	59.2	56.9	plot_263	N180	79.6	77.1	plot_425	N270	75.2	80.5
plot_102	N0	79.7	82.7	plot_264	N180	68.6	77.2	plot_426	N270	73.2	69.3
plot_103	N0	59.0	63.6	plot_265	N180	70.0	76.1	plot_427	N270	95.8	84.7
plot_104	N0	65.4	68.7	plot_266	N180	79.2	81.7	plot_428	N270	63.7	64.9
plot_105	N0	69.4	71.2	plot_267	N180	65.8	70.1	plot_429	N270	73.4	76.0
plot_106	N0	64.6	66.5	plot_268	N180	75.8	69.9	plot_430	N270	79.5	79.4
plot_107	N0	59.2	64.7	plot_269	N180	80.3	79.2	plot_431	N270	88.8	81.9
plot_108	N0	62.8	66.5	plot_270	N180	68.6	74.2	plot_432	N270	94.7	85.7
plot_109	N0	61.3	61.7	plot_271	N180	66.7	74.2	plot_433	N270	72.1	74.8
plot_110	N0	55.5	58.0	plot_272	N180	58.8	65.5	plot_434	N270	91.3	85.1
plot_111	N0	67.9	69.8	plot_273	N180	86.9	82.7	plot_435	N270	86.3	86.1
plot_112	N0	71.3	76.1	plot_274	N180	58.1	62.7	plot_436	N270	83.3	83.4
plot_113	N0	78.9	84.4	plot_275	N180	81.0	84.7	plot_437	N270	77.0	69.8
plot_114	N0	71.0	76.4	plot_276	N180	72.5	76.4	plot_438	N270	84.9	76.3
plot_115	N0	59.4	69.0	plot_277	N180	67.1	78.8	plot_439	N270	70.1	72.4
plot_116	N0	63.0	67.6	plot_278	N180	75.0	79.4	plot_440	N270	81.7	81.3
plot_117	N0	65.0	66.9	plot_279	N180	60.9	62.8	plot_441	N270	85.9	87.2
plot_118	N0	70.2	74.1	plot_280	N180	84.5	83.0	plot_442	N270	78.6	72.7
plot_119	N0	66.7	71.3	plot_281	N180	97.8	117.7	plot_443	N270	65.9	72.0
plot_120	N0	62.4	70.3	plot_282	N180	73.0	76.2	plot_444	N270	70.9	73.6
plot_121	N0	56.2	58.3	plot_283	N180	74.4	82.2	plot_445	N270	65.6	65.0
plot_122	N0	62.4	63.4	plot_284	N180	74.3	80.8	plot_446	N270	81.5	79.3
plot_123	N0	53.4	58.6	plot_285	N180	75.3	81.8	plot_447	N270	84.8	85.5
plot_124	N0	69.2	72.9	plot_286	N180	89.8	87.7	plot_448	N270	78.4	80.7
plot_125	N0	73.0	75.5	plot_287	N180	78.1	73.9	plot_449	N270	79.8	83.2
plot_126	N0	62.0	65.5	plot_288	N180	62.4	67.4	plot_450	N270	76.4	79.4
plot_127	N0	56.2	57.1	plot_289	N180	67.9	75.9	plot_451	N270	68.0	74.8
plot_128	N0	62.9	67.7	plot_290	N180	97.6	81.7	plot_452	N270	79.7	78.7
plot_129	N0	69.4	75.9	plot_291	N180	73.1	73.2	plot_453	N270	75.3	78.4
plot_130	N0	67.8	76.8	plot_292	N180	70.1	76.4	plot_454	N270	108.7	116.8
plot_131	N0	61.7	67.5	plot_293	N180	73.2	69.5	plot_455	N270	75.1	71.8
plot_132	N0	60.4	69.3	plot_294	N180	75.0	75.1	plot_456	N270	65.7	71.0
plot_133	N0	48.6	50.8	plot_295	N180	84.1	83.8	plot_457	N270	71.0	78.4

plot_134	N0	56.0	60.8	plot_296	N180	78.4	79.9	plot_458	N270	80.2	86.1
plot_135	N0	72.0	70.8	plot_297	N180	80.9	73.6	plot_459	N270	84.8	72.0
plot_136	N0	70.8	75.4	plot_298	N180	76.2	80.8	plot_460	N270	76.0	74.5
plot_137	N0	62.9	64.1	plot_299	N180	77.9	76.3	plot_461	N270	81.2	82.4
plot_138	N0	56.5	64.2	plot_300	N180	73.6	70.2	plot_462	N270	54.3	63.2
plot_139	N0	63.8	63.9	plot_301	N180	76.3	79.7	plot_463	N270	90.1	86.4
plot_140	N0	65.1	64.8	plot_302	N180	81.6	78.5	plot_464	N270	71.5	71.1
plot_141	N0	61.3	65.6	plot_303	N180	77.8	81.7	plot_465	N270	65.7	62.3
plot_142	N0	64.8	71.0	plot_304	N180	73.2	70.1	plot_466	N270	75.6	78.5
plot_143	N0	83.2	82.4	plot_305	N180	67.6	70.0	plot_467	N270	64.5	68.9
plot_144	N0	60.4	63.3	plot_306	N180	68.6	68.6	plot_468	N270	67.0	73.8
plot_145	N0	61.5	68.4	plot_307	N180	72.6	79.0	plot_469	N270	72.4	79.8
plot_146	N0	64.3	62.5	plot_308	N180	73.2	79.6	plot_470	N270	70.3	76.6
plot_147	N0	61.9	67.6	plot_309	N180	67.2	75.0	plot_471	N270	69.9	71.5
plot_148	N0	67.2	69.7	plot_310	N180	81.6	73.3	plot_472	N270	77.3	74.9
plot_149	N0	60.4	64.7	plot_311	N180	67.6	66.7	plot_473	N270	72.1	66.1
plot_150	N0	53.7	58.0	plot_312	N180	67.6	73.7	plot_474	N270	96.5	95.4
plot_151	N0	47.7	55.1	plot_313	N180	84.0	84.7	plot_475	N270	67.9	68.5
plot_152	N0	58.2	63.3	plot_314	N180	76.3	78.2	plot_476	N270	72.2	82.0
plot_153	N0	50.8	51.1	plot_315	N180	56.1	60.6	plot_477	N270	57.8	56.8
plot_154	N0	60.4	64.1	plot_316	N180	79.5	84.0	plot_478	N270	77.5	74.9
plot_155	N0	60.2	63.3	plot_317	N180	73.3	76.5	plot_479	N270	74.1	74.6
plot_156	N0	62.4	65.6	plot_318	N180	81.0	85.6	plot_480	N270	70.0	71.1
plot_157	N0	90.0	93.7	plot_319	N180	86.3	85.4	plot_481	N270	70.8	76.7
plot_158	N0	57.6	57.5	plot_320	N180	74.5	82.3	plot_482	N270	85.3	79.3
plot_159	N0	61.1	64.5	plot_321	N180	73.8	75.0	plot_483	N270	72.6	72.8
plot_160	N0	71.1	70.1	plot_322	N180	75.9	75.2	plot_484	N270	77.2	76.6
plot_161	N0	62.9	61.0	plot_323	N180	95.0	85.3	plot_485	N270	77.5	78.1
plot_162	N0	65.8	65.7	plot_324	N180	64.1	62.9	plot_486	N270	72.8	74.7

Table S2. CropQuant-3D's traits analyses of 81 six-metre plots of ZM & NM varieties under three N treatments (0, 180 and 270 kg N ha^{-1}), generated by the GUI-based software.

Plot_ID	Date	Variety	Row_ no	Col_ no	N_Treat	3DCI	3D Surf.	3DVI	3DPI	Canopy Cov.	Height
Plt_1	16/05/20	NMzi1	6	5	N0	0.524	0.136	0.058	0.460	0.579	70.9
Plt_2	16/05/20	NMzi1	17	2	N0	0.571	0.246	0.372	0.466	0.573	70.4
Plt_3	16/05/20	NMzi1	22	6	N0	0.603	0.331	0.386	0.469	0.571	60.4
Plt_4	16/05/20	NMzi1019	2	4	N0	0.549	0.223	0.149	0.457	0.493	68.5
Plt_5	16/05/20	NMzi1019	18	1	N0	0.566	0.278	0.198	0.468	0.488	59.0
Plt_6	16/05/20	NMzi1019	25	1	N0	0.548	0.285	0.659	0.477	0.495	61.5
Plt_7	16/05/20	NM26	1	3	N0	0.511	0.206	0.101	0.456	0.514	59.6
Plt_8	16/05/20	NM26	16	1	N0	0.522	0.217	0.146	0.468	0.496	59.0
Plt_9	16/05/20	NM26	26	1	N0	0.502	0.359	0.472	0.478	0.452	47.7
Plt_10	16/05/20	ZM4	6	3	N0	0.546	0.294	0.240	0.484	0.617	70.3
Plt_11	16/05/20	ZM4	11	4	N0	0.548	0.360	0.580	0.488	0.598	71.8
Plt_12	16/05/20	ZM5	6	4	N0	0.491	0.154	0.257	0.466	0.646	73.4
Plt_13	16/05/20	ZM5	14	5	N0	0.561	0.525	0.291	0.468	0.604	76.4
Plt_14	16/05/20	ZM5	27	2	N0	0.538	0.529	0.601	0.481	0.555	57.6
Plt_15	16/05/20	ZM8	6	1	N0	0.535	0.412	0.355	0.477	0.496	65.3
Plt_16	16/05/20	ZM8	14	4	N0	0.493	0.500	0.537	0.485	0.655	71.1
Plt_17	16/05/20	ZM8	25	4	N0	0.514	0.285	0.191	0.465	0.578	67.2
Plt_18	16/05/20	ZM10	4	1	N0	0.59	0.297	0.208	0.479	0.480	61.9
Plt_19	16/05/20	ZM10	17	4	N0	0.554	0.170	0.196	0.462	0.580	68.5
Plt_20	16/05/20	ZM11	4	2	N0	0.547	0.369	0.372	0.480	0.553	67.2
Plt_21	16/05/20	ZM11	11	1	N0	0.612	0.299	0.088	0.458	0.506	66.9
Plt_22	16/05/20	ZM11	26	4	N0	0.54	0.253	0.268	0.467	0.546	60.4
Plt_23	16/05/20	ZM12	1	5	N0	0.593	0.144	0.303	0.457	0.632	73.5
Plt_24	16/05/20	ZM12	12	1	N0	0.556	0.421	0.432	0.466	0.528	66.2
Plt_25	16/05/20	ZM12	20	1	N0	0.573	0.544	0.618	0.481	0.488	59.4
Plt_26	16/05/20	ZM168	3	3	N0	0.494	0.405	0.227	0.472	0.613	72.1
Plt_27	16/05/20	ZM168	16	5	N0	0.474	0.353	0.543	0.475	0.588	70.3
Plt_28	16/05/20	ZM09196	7	4	N0	0.488	0.259	0.247	0.479	0.590	57.9
Plt_29	16/05/20	ZM09196	21	3	N0	0.481	0.084	0.162	0.455	0.549	53.4
Plt_30	16/05/20	NMzi1	11	1	N180	0.582	0.417	0.529	0.502	0.523	75.8
Plt_31	16/05/20	NMzi1	20	6	N180	0.563	0.618	0.665	0.515	0.534	73.0
Plt_32	16/05/20	NMzi1019	4	6	N180	0.509	0.362	0.374	0.469	0.552	78.7
Plt_33	16/05/20	NMzi1019	14	6	N180	0.524	0.356	0.399	0.456	0.534	68.1
Plt_34	16/05/20	NMzi1019	25	3	N180	0.507	0.388	0.138	0.459	0.579	67.2
Plt_35	16/05/20	NM26	9	3	N180	0.421	0.461	0.412	0.494	0.618	65.5
Plt_36	16/05/20	NM26	24	5	N180	0.511	0.405	0.153	0.476	0.601	67.6
Plt_37	16/05/20	ZM4	8	5	N180	0.524	0.325	0.216	0.459	0.623	76.4
Plt_38	16/05/20	ZM4	10	2	N180	0.489	0.358	0.488	0.464	0.628	81.9
Plt_39	16/05/20	ZM4	21	1	N180	0.512	0.441	0.715	0.477	0.547	74.4
Plt_40	16/05/20	ZM5	4	4	N180	0.418	0.467	0.437	0.489	0.629	83.9
Plt_41	16/05/20	ZM5	18	2	N180	0.457	0.449	0.333	0.475	0.594	79.2
Plt_42	16/05/20	ZM8	3	3	N180	0.451	0.320	0.541	0.491	0.607	81.9

Plt_43	16/05/20	ZM8	13	4	N180	0.438	0.242	0.115	0.465	0.647	84.9
Plt_44	16/05/20	ZM8	27	1	N180	0.429	0.262	0.122	0.485	0.582	86.3
Plt_45	16/05/20	ZM10	6	1	N180	0.584	0.393	0.506	0.483	0.549	81.5
Plt_46	16/05/20	ZM10	17	6	N180	0.554	0.475	0.530	0.489	0.522	68.6
Plt_47	16/05/20	ZM11	1	2	N180	0.466	0.234	0.274	0.454	0.606	77.6
Plt_48	16/05/20	ZM11	18	6	N180	0.434	0.198	0.280	0.472	0.537	68.6
Plt_49	16/05/20	ZM11	23	5	N180	0.5	0.605	0.481	0.489	0.577	77.9
Plt_50	16/05/20	ZM12	1	1	N180	0.548	0.319	0.318	0.457	0.597	79.8
Plt_51	16/05/20	ZM12	12	1	N180	0.599	0.333	0.541	0.493	0.513	68.4
Plt_52	16/05/20	ZM12	19	6	N180	0.559	0.481	0.622	0.486	0.556	72.5
Plt_53	16/05/20	ZM168	15	5	N180	0.496	0.473	0.239	0.464	0.602	76.6
Plt_54	16/05/20	ZM168	20	1	N180	0.516	0.479	0.312	0.470	0.523	67.1
Plt_55	16/05/20	ZM09196	11	3	N180	0.37	0.235	0.157	0.469	0.572	65.8
Plt_56	16/05/20	ZM09196	20	3	N180	0.409	0.356	0.215	0.478	0.591	60.9
Plt_57	16/05/20	NMzi1	18	3	N270	0.493	0.733	0.678	0.526	0.628	73.4
Plt_58	16/05/20	NMzi1	23	2	N270	0.519	0.566	0.689	0.497	0.608	80.2
Plt_59	16/05/20	NMzi1019	2	1	N270	0.45	0.548	0.765	0.524	0.624	77.1
Plt_60	16/05/20	NMzi1019	14	4	N270	0.472	0.493	0.540	0.485	0.572	73.8
Plt_61	16/05/20	NMzi1019	19	5	N270	0.492	0.554	0.662	0.503	0.650	77.0
Plt_62	16/05/20	NM26	5	5	N270	0.404	0.683	0.714	0.516	0.598	77.1
Plt_63	16/05/20	NM26	12	1	N270	0.501	0.565	0.559	0.503	0.518	65.5
Plt_64	16/05/20	ZM4	5	1	N270	0.492	0.591	0.576	0.488	0.569	77.9
Plt_65	16/05/20	ZM4	18	5	N270	0.471	0.749	0.643	0.515	0.674	88.8
Plt_66	16/05/20	ZM5	3	2	N270	0.43	0.375	0.457	0.506	0.579	101.0
Plt_67	16/05/20	ZM5	12	3	N270	0.41	0.541	0.527	0.482	0.588	89.4
Plt_68	16/05/20	ZM8	13	3	N270	0.449	0.253	0.279	0.470	0.632	82.8
Plt_69	16/05/20	ZM8	20	3	N270	0.457	0.167	0.222	0.464	0.607	85.9
Plt_70	16/05/20	ZM10	7	6	N270	0.59	0.475	0.270	0.465	0.603	87.1
Plt_71	16/05/20	ZM10	17	5	N270	0.523	0.523	0.311	0.499	0.658	75.2
Plt_72	16/05/20	ZM10	27	3	N270	0.541	0.582	0.539	0.510	0.588	72.6
Plt_73	16/05/20	ZM11	7	3	N270	0.362	0.361	0.223	0.468	0.579	80.7
Plt_74	16/05/20	ZM11	26	2	N270	0.572	0.370	0.481	0.488	0.591	72.2
Plt_75	16/05/20	ZM12	7	1	N270	0.531	0.525	0.265	0.482	0.537	69.6
Plt_76	16/05/20	ZM12	15	3	N270	0.361	0.341	0.170	0.481	0.620	83.5
Plt_77	16/05/20	ZM12	21	4	N270	0.486	0.656	0.632	0.528	0.583	78.4
Plt_78	16/05/20	ZM168	6	2	N270	0.545	0.515	0.298	0.490	0.613	86.7
Plt_79	16/05/20	ZM168	23	5	N270	0.525	0.540	0.630	0.485	0.621	81.2
Plt_80	16/05/20	ZM09196	5	2	N270	0.464	0.429	0.290	0.480	0.650	67.0
Plt_81	16/05/20	ZM09196	23	6	N270	0.497	0.457	0.673	0.506	0.602	67.9

Table S3. Plot-based correlation performance metrics evaluate CropQuant-3D-measured height values using manual height measurement under three N treatments (0, 180 and 270 kg N ha^{-1}).

**N0 (plot heights, n = 162)**									
**Regression statistics**			**ANOVA**						
Multiple R	0.931766			df	SS	MS	F	Significance F	
R Square	0.868189		Regression statistics	1	7947.602	7947.602	1053.856	2.67E-72	
Adjusted R Square	0.867365		Residual value	160	1206.632	7.54145			
Standard error	2.74617		total	161	9154.234				
Number	162								
	Coefficients	Standard error	t Stat	P-value	Lower 95%	Upper 95%	Lower 95%	Upper 95%	
Intercept	6.728844	1.945207	3.459191861	0.000695	2.887252	10.57044	2.887252	10.57044	
X Variable 1	0.953058	0.029358	32.46314717	2.67E-72	0.895079	1.011038	0.895079	1.011038	
**N180 (plot heights, n = 162)**									
**Regression statistics**			**ANOVA**						
Multiple R	0.826141			df	SS	MS	F	Significance F	
R Square	0.682509		Regression statistics	1	7971.013	7971.013	343.9516	1.05E-41	
Adjusted R Square	0.680525		Residual value	160	3707.97	23.17481			
Standard error	4.814023		total	161	11678.98				
Number	162								
	Coefficients	Standard error	t Stat	P-value	Lower 95%	Upper 95%	Lower 95%	Upper 95%	
Intercept	17.0768	3.286374	5.196244871	6.12E-07	10.58654	23.56707	10.58654	23.56707	
X Variable 1	0.791776	0.042693	18.54593138	1.05E-41	0.707462	0.87609	0.707462	0.87609	
**N270 (plot heights, n = 162)**									
**Regression statistics**			**ANOVA**						
Multiple R	0.86731			df	SS	MS	F	Significance F	
R Square	0.752226		Regression statistics	1	8525.568	8525.568	485.7495	2.42E-50	
Adjusted R Square	0.750677		Residual value	160	2808.219	17.55137			
Standard error	4.189435		total	161	11333.79				
Number	162								
	Coefficients	Standard error	t Stat	P-value	Lower 95%	Upper 95%	Lower 95%	Upper 95%	
Intercept	15.73741	2.839551	5.542219129	1.21E-07	10.12958	21.34525	10.12958	21.34525	
X Variable 1	0.790245	0.035855	22.03972509	2.42E-50	0.719434	0.861056	0.719434	0.861056	

Table S4. Variety-based correlation performance metrics evaluate CropQuant-3D-measured height values using manual height measurement under three N treatments.

**N0 (Variety Heights, n = 54)**								
**Regression statistics**			**ANOVA**					
Multiple R	0.95908			df	SS	MS	F	Significance F
R Square	0.919841		Regression statistics	1	2159.876231	2159.876231	596.708917	3.64827E-30
Adjusted R Square	0.918299		Residual value	52	188.2216954	3.619647988		
Standard error	1.902537		total	53	2348.097926			
Number	54							
	Coefficients	Standard error	t Stat	P-value	Lower 95%	Upper 95%	Lower 95%	Upper 95%
Intercept	5.267929	2.641661	1.994173	0.047389	-0.032951	10.568808	-0.032951	10.568808
X Variable 1	0.975244	0.039923	24.427626	3.64827E-30	0.895131	1.055357	0.895131	1.055357
**N180 (Variety Heights, n = 54)**								
**Regression statistics**			**ANOVA**					
Multiple R	0.91583			df	SS	MS	F	Significance F
R Square	0.838751		Regression statistics	1	2734.5725	2734.5725	270.4827	2.97455E-22
Adjusted R Square	0.8356501		Residual value	52	525.7184	10.109972		
Standard error	3.179618		total	53	3260.291			
Number	54							
	Coefficients	Standard error	t Stat	P-value	Lower 95%	Upper 95%	Lower 95%	Upper 95%
Intercept	9.741162	4.1499473	2.3472978	0.0227522	1.4136837	18.068641	1.4136837	18.068641
X Variable 1	0.887709	0.053976	16.446358	2.97455E-22	0.7793982	0.9960198	0.7793982	0.9960198
**N270 (Variety Heights, n = 54)**								
**Regression statistics**			**ANOVA**					
Multiple R	0.95979			df	SS	MS	F	Significance F
R Square	0.9211995		Regression statistics	1	2665.8185	2665.8185	607.89394	2.33748E-30
Adjusted R Square	0.9196841		Residual value	52	228.03741	4.3853349		
Standard error	2.094119		total	53	2893.8559			
Number	54							
	Coefficients	Standard error	t Stat	P-value	Lower 95%	Upper 95%	Lower 95%	Upper 95%
Intercept	6.636609	2.9043144	2.2850864	0.0264165	0.808676	12.464542	0.808676	12.464542
X Variable 1	0.9059413	0.036744	24.655505	2.33748E-30	0.8322091	0.9796735	0.8322091	0.9796735

Table S5. Correlation performance metrics evaluate CropQuant-3D-measured canopy surface area trait using manual grain number per unit area (GN m^{-2}) scores under three N treatments.

**N0 (3D canopy surface area, n = 29)**								
**Regression statistics**		**ANOVA**						
Multiple R	0.874228			df	SS	MS	F	Significance F
R Square	0.764274		Regression statistics	1	10805.54	10805.54	87.53996	5.8E-10
Adjusted R Square	0.755544		Residual value	27	3332.759	123.4355		
Standard error	11.11015		total	28	14138.3			
Number	29							
	Coefficients	Standard error	t Stat	P-value	Lower 95%	Upper 95%	Lower 95%	Upper 95%
Intercept	44.56689	4.896092	9.102543	1.03E-09	34.52094	54.61284	34.52094	54.61284
X Variable 1	224.662	24.01189	9.356279	5.8E-10	175.3936	273.9303	175.3936	273.9303
**N180 (3D canopy surface area, n =27)**								
**Regression statistics**		**ANOVA**						
Multiple R	0.843796			df	SS	MS	F	Significance F
R Square	0.711992		Regression statistics	1	8080.035	8080.035	61.80329	3.23E-08
Adjusted R Square	0.700472		Residual value	25	3268.448	130.7379		
Standard error	11.43407		total	26	11348.48			
Number	27							
	Coefficients	Standard error	t Stat	P-value	Lower 95%	Upper 95%	Lower 95%	Upper 95%
Intercept	75.35456	6.061915	12.43082	3.36E-12	62.86981	87.8393	62.86981	87.8393
X Variable 1	150.5636	19.152	7.861507	3.23E-08	111.1193	190.0079	111.1193	190.0079
**N270 (3D canopy surface area, n = 26)**								
**Regression statistics**		**ANOVA**						
Multiple R	0.845486			df	SS	MS	F	Significance F
R Square	0.714847		Regression statistics	1	25529.69	25529.69	57.65835	1.04E-07
Adjusted R Square	0.702449		Residual value	23	10183.83	442.7752		
Standard error	21.04222		total	24	35713.51			
Number	26							
	Coefficients	Standard error	t Stat	P-value	Lower 95%	Upper 95%	Lower 95%	Upper 95%
Intercept	79.44645	9.040059	8.788267	8.24E-09	60.74566	98.14723	60.74566	98.14723
X Variable 1	157.1462	20.69535	7.59331	1.04E-07	114.3346	199.9578	114.3346	199.9578

Table S6. Correlation performance metrics evaluate CropQuant-3D-measured 3DCI trait using manual spike number per unit area (SN m^{-2}) scores under three N treatments.

**N0 (3DCI, n = 29)**								
**Regression statistics**		**ANOVA**						
Multiple R	0.901538			df	SS	MS	F	Significance F
R Square	0.812772		Regression statistics	1	15611.61	15611.61	117.2089	2.52E-11
Adjusted R Square	0.805837		Residual value	27	3596.258	133.1947		
Standard error	11.541		total	28	19207.87			
Number	29							
	Coefficients	Standard error	t Stat	P-value	Lower 95%	Upper 95%	Lower 95%	Upper 95%
Intercept	497.3495	27.89337	17.83038	1.83E-16	440.117	554.582	440.117	554.582
X Variable 1	-562.5	51.95679	-10.8263	2.52E-11	-669.107	-455.894	-669.107	-455.894
**N180 (3DCI, n =27)**								
**Regression statistics**		**ANOVA**						
Multiple R	0.879459			df	SS	MS	F	Significance F
R Square	0.773448		Regression statistics	1	21507.12	21507.12	85.35007	1.55E-09
Adjusted R Square	0.764386		Residual value	25	6299.678	251.9871		
Standard error	15.8741		total	26	27806.79			
Number	27							
	Coefficients	Standard error	t Stat	P-value	Lower 95%	Upper 95%	Lower 95%	Upper 95%
Intercept	370.1517	17.68056	20.93551	2.3E-17	333.7379	406.5655	333.7379	406.5655
X Variable 1	-326.489	35.33999	-9.23851	1.55E-09	-399.273	-253.705	-399.273	-253.705
**N270 (3DCI, n = 26)**								
**Regression statistics**		**ANOVA**						
Multiple R	0.901716			df	SS	MS	F	Significance F
R Square	0.811426		Regression statistics	1	17043.03	17043.03	113.2768	2.33E-10
Adjusted R Square	0.823888		Residual value	23	3460.458	150.4547		
Standard error	12.266		total	24	20503.48			
Number	25							
	Coefficients	Standard error	t Stat	P-value	Lower 95%	Upper 95%	Lower 95%	Upper 95%
Intercept	366.4447	13.43573	27.2739	5.11E-19	338.6508	394.2386	338.6508	394.2386
X Variable 1	-275.968	25.92914	-10.6432	2.33E-10	-329.606	-222.329	-329.606	-222.329

Table S7. Cost comparison between backpack LiDAR, UAV airborne LiDAR, and the handheld laser scanning system, with brief technical specifications.

LiDAR system	System costs (academic price)	Brief technical spec.	References
Robin (backpack)	US$350,000-375,000 with TerraSoild software (2018)	VUX-1 HA scanner, 1.5-200 m eff. range, 5-10 mm accuracy (outdoor)	www.3dlasermapping.com (discontinued)
BMS3D-HD (backpack)	US$310,000-330,000 with BMS3D software (2021)	HDL-32 & VLP-16 scanners, 0.5-100 m eff. range, 20 mm accuracy (outdoor)	www.viametrisbusiness.com (France)
Pegasus (backpack)	US$330,000-360,000 with local software (2021)	Dual VLP-16 scanner, 80 m eff. range, 10-30 mm accuracy (outdoor)	www.leica-geosystems.com (Switzerland)
GeoSLAM (backpack)	US$300,000-320,000 with ORBIT software (2020)	Dual ZEB Discovery scanner, 100 m eff. range, 10-30 mm accuracy (outdoor)	geoslam.com (UK)
SLAM-based (backpack)	A low-cost solution with Forest3D software (2020)	Dual Velodyne Puck VLP-16 sensors, 100 m eff. range, 30 mm accuracy	(Su et al., 2020) velodynelidar.com (US)
Airborne LiDAR	US$125,000-150,000 with SpatialExplorer (2021)	SCOUT and RANGER series, 100 m range, 50-55 mm accuracy	www.phoenixlidar.com (US)
Handheld LiDAR	US$25,000-50,000 with GeoSLAM Hub & Draw software (2021)	ZEB-Horizon scanner, 100 m eff. range, 10-30 mm accuracy	geoslam.com (UK)

Reference:

Su Y, Guo Q, Jin S, Guan H, Sun X, Ma Q, Hu T, Wang R, Li Y (2020) The Development and Evaluation of a Backpack LiDAR System for Accurate and Efficient Forest Inventory. IEEE Geosci Remote Sens Lett 1–5

Table S8. Three split fields used to study three replicates of 54 wheat varieties under three levels of N fertiliser treatments (i.e. 0, 180, and 270 kg N ha^{-1}). Crops were planted in six m^2 (2×3 m) plots, 486 plots in total.

Low N treatments (0 kg N ha^{-1}, n = 162 plots)

Var_ID	Variety	Var_ID	Variety	Var_ID	Variety	Var_ID	Variety	Var_ID	Variety	Var_ID	Variety
A3009	Huamai-6	A3018	Xumai-32	A3027	Ningmai-26	A3036	Dongmai-1301	A3045	Zhenmai-12	A3054	Yangfumai-5
A3008	P14	A3017	CP20-39-11-2	A3026	Kenong-9204	A3035	Ningmaizi-1019	A3044	CP20-39-11-1	A3053	CP02-63-13-1
A3007	Nannong-06Y86	A3016	Guohong-9	A3025	Zhenmai-168	A3034	Shengxuan-6	A3043	Sukemai-1	A3052	Tianmin-108
A3006	Zhenmai-10	A3015	Zhenmai-11	A3024	Yangmai-16	A3033	Su-553	A3042	Wanyu-2	A3051	CP02-9-4-8-2
A3005	Huaimai-30	A3014	Yangmai-158	A3023	CP02-62-1-2-2-3F10	A3032	CP01-39-3-2-4	A3041	Sumai-8	A3050	Wanmai-108
A3004	Zhenmai-8	A3013	Yangmai-25	A3022	Zhenmai-4	A3031	Zhenmai-5	A3040	Ningzimai-1	A3049	Zhen-9523
A3003	Yangmai-20	A3012	CP01-39-17-1-3	A3021	Ningmai-13	A3030	Zhenmai-09196	A3039	Lvyu-098	A3048	Sumai-3
A3002	P124	A3011	Wanmai-52	A3020	CP02-8-5-6-1	A3029	Aikang-58	A3038	Baoji-0601	A3047	Yangfumai-8
A3001	Yangfumai-6	A3010	CP02-8-5-5-21	A3019	Weilai-1216	A3028	CP01-39-3-204	A3037	Pingmai-108	A3046	CP03-28-1-1
A2009	CP01-39-3-2-4	A2018	Baoji-0601	A2027	CP20-39-11-2	A2036	Ningmai-13	A2045	Zhen-9523	A2054	Sukemai-1
A2008	Zhenmai-11	A2017	Su-553	A2026	Yangmai-25	A2035	Zhenmai-4	A2044	Huaimai-30	A2053	Huamai-6
A2007	Zhenmai-12	A2016	P14	A2025	CP03-28-1-1	A2034	Kenong-9204	A2043	Wanmai-108	A2052	Yangfumai-6
A2006	Yangmai-16	A2015	Tianmin-108	A2024	CP01-39-3-204	A2033	Guohong-9	A2042	Wanmai-52	A2051	CP01-39-17-1-3
A2005	CP02-62-1-2-2-3F10	A2014	Weilai-1216	A2023	P124	A2032	Zhenmai-8	A2041	Zhenmai-5	A2050	Yangfumai-8
A2004	Yangmai-20	A2013	Yangmai-158	A2022	CP02-63-13-1	A2031	Yangfumai-5	A2040	Dongmai-1301	A2049	Lvyu-098
A2003	Ningmai-26	A2012	Sumai-8	A2021	Xumai-32	A2030	Shengxuan-6	A2039	Zhenmai-168	A2048	Sumai-3
A2002	Zhenmai-09196	A2011	Ningzimai-1	A2020	Wanyu-2	A2029	Zhenmai-10	A2038	Aikang-58	A2047	CP02-8-5-5-21
A2001	Ningmaizi-1019	A2010	Nannong-06Y86	A2019	CP02-8-5-6-1	A2028	Pingmai-108	A2037	CP02-9-4-8-2	A2046	CP20-39-11-1
A1009	Kenong-9204	A1018	Ningmai-13	A1027	Zhenmai-168	A1036	Wanmai-108	A1045	P14	A1054	Zhen-9523
A1008	Zhenmai-12	A1017	Su-553	A1026	Yangfumai-6	A1035	Zhenmai-10	A1044	CP02-8-5-6-1	A1053	CP01-39-3-204
A1007	Hiamai-6	A1016	Guohong-9	A1025	Zhenmai-09196	A1034	Wanyu-2	A1043	Yangmai-16	A1052	Dongmai-1301
A1006	Lvyu-098	A1015	Sumai-3	A1024	Yangfumai-8	A1033	CP20-39-11-1	A1042	CP02-9-4-8-2	A1051	Ningzimai-1
A1005	Weilai-1216	A1014	CP01-39-3-2-4	A1023	CP20-39-11-2	A1032	Zhenmai-4	A1041	Pingmai-108	A1050	CP02-62-1-2-2-3F10
A1004	Yangmai-158	A1013	CP01-39-17-1-3	A1022	Shengxuan-6	A1031	CP02-63-13-1	A1040	CP02-8-5-5-21	A1049	Tianmin-108
A1003	Ningmaizi-1019	A1012	Nannong-06Y86	A1021	Huaimai-30	A1030	Zhenmai-8	A1039	Sumai-8	A1048	Sukemai-1
A1002	Ningmai-26	A1011	Baoji-0601	A1020	Aikang-58	A1029	Zhenmai-11	A1038	Yangmai-25	A1047	Xumai-32
A1001	CP03-28-1-1	A1010	Zhenmai-5	A1019	Yangfumai-5	A1028	P124	A1037	Yangmai-20	A1046	Wanmai-52

Medium N treatments (180 kg N ha^{-1}, n = 162 plots)

Var_ID	Variety	Var_ID	Variety	Var_ID	Variety	Var_ID	Variety	Var_ID	Variety	Var_ID	Variety
B3009	Zhenmai-12	B3018	Zhenmai-11	B3027	Yangmai-20	B3036	Xumai-32	B3045	Weilai-1216	B3054	Yangmai-25
B3008	Pingmai-108	B3017	P14	B3026	Shengxuan-6	B3035	CP02-9-4-8-2	B3044	Aikang-58	B3053	CP02-8-5-6-1
B3007	Ningmai-13	B3016	Sumai-3	B3025	Zhenmai-8	B3034	Sukemai-1	B3043	CP03-28-1-1	B3052	Yangfumai-8
B3006	Dongmai-1301	B3015	CP02-63-13-1	B3024	Baoji-0601	B3033	Zhenmai-5	B3042	Kenong-9204	B3051	Ningmaizi-1019
B3005	Tianmin-108	B3014	Nannong-06Y86	B3023	Ningzimai-1	B3032	Zhenmai-09196	B3041	CP01-39-3-2-4	B3050	P124
B3004	Zhenmai-10	B3013	Zhenmai-168	B3022	CP01-39-17-1-3	B3031	CP01-39-3-204	B3040	Wanmai-108	B3049	CP20-39-11-1
B3003	Zhen-9523	B3012	CP02-62-1-2-2-3F10	B3021	Wanmai-52	B3030	CP02-8-5-5-21	B3039	Yangfumai-5	B3048	Su-553
B3002	Huamai-6	B3011	Yangmai-16	B3020	Yangmai-158	B3029	Lvyu-098	B3038	Zhenmai-4	B3047	Sumai-8
B3001	Wanyu-2	B3010	Guohong-9	B3019	Ningmai-26	B3028	CP20-39-11-2	B3037	Huaimai-30	B3046	Yangfumai-6
B2009	Weilai-1216	B2018	Zhenmai-4	B2027	Tianmin-108	B2036	Lvyu-098	B2045	CP02-63-13-1	B2054	Yangmai-25
B2008	Ningzimai-1	B2017	Nannong-06Y86	B2026	Zhenmai-09196	B2035	Huaimai-30	B2044	Yangfumai-6	B2053	Dongmai-1301
B2007	Zhenmai-12	B2016	Yangfumai-5	B2025	CP02-62-1-2-2-3F10	B2034	Guohong-9	B2043	Yangmai-20	B2052	Kenong-9204
B2006	Ningmai-13	B2015	Yangfumai-8	B2024	Zhen-9523	B2033	Zhenmai-8	B2042	P124	B2051	Wanmai-52
B2005	Aikang-58	B2014	CP03-28-1-1	B2023	CP02-9-4-8-2	B2032	Xumai-32	B2041	Su-553	B2050	Ningmaizi-1019
B2004	Huamai-6	B2013	CP20-39-11-1	B2022	Shengxuan-6	B2031	Sumai-3	B2040	Zhenmai-168	B2049	CP01-39-3-2-4
B2003	Sukemai-1	B2012	CP01-39-17-1-3	B2021	Yangmai-158	B2030	P14	B2039	CP02-8-5-5-21	B2048	CP01-39-3-204
B2002	CP20-39-11-2	B2011	Baoji-0601	B2020	Wanyu-2	B2029	Sumai-8	B2038	Yangmai-16	B2047	Zhenmai-10
B2001	Wanmai-108	B2010	Zhenmai-5	B2019	Ningmai-26	B2028	Pingmai-108	B2037	CP02-8-5-6-1	B2046	Zhenmai-11
B1009	Wanmai-52	B1018	Ningmai-13	B1027	CP01-39-17-1-3	B1036	Aikang-58	B1045	CP02-8-5-6-1	B1054	Zhenmai-12
B1008	Zhenmai-168	B1017	Yangfumai-6	B1026	Zhenmai-09196	B1035	Yangmai-16	B1044	CP03-28-1-1	B1053	Ningzimai-1
B1007	Zhenmai-4	B1016	Sukemai-1	B1025	Baoji-0601	B1034	P124	B1043	CP20-39-11-1	B1052	CP02-62-1-2-2-3F10
B1006	CP01-39-3-2-4	B1015	CP02-8-5-5-21	B1024	CP02-9-4-8-2	B1033	Su-553	B1042	Huamai-6	B1051	Yangfumai-5
B1005	Wanmai-108	B1014	Tianmin-108	B1023	Guohong-9	B1032	Zhenmai-5	B1041	Zhenmai-11	B1050	Kenong-9204
B1004	Shengxuan-6	B1013	Nannong-06Y86	B1022	Yangfumai-8	B1031	Xumai-32	B1040	Ningmai-26	B1049	Yangmai-20
B1003	Lvyu-098	B1012	CP02-63-13-1	B1021	Ningmaizi-1019	B1030	Zhenmai-9523	B1039	Huaimai-30	B1048	CP01-39-3-204
B1002	Sumai-3	B1011	Dongmai-1301	B1020	Weilai-1216	B1029	Yangmai-25	B1038	Sumai-8	B1047	P14
B1001	Zhenmai-8	B1010	Zhenmai-10	B1019	Yangmai-158	B1028	Pingmai-108	B1037	CP20-39-11-2	B1046	Wanyu-2

High N treatments (270 kg N ha^{-1}, n = 162 plots)

Var_ID	Variety	Var_ID	Variety	Var_ID	Variety	Var_ID	Variety	Var_ID	Variety	Var_ID	Variety
C3009	CP01-39-17-1-3	C3018	Yangmai-16	C3027	Wanyu-2	C3036	Yangmai-20	C3045	CP02-63-13-1	C3054	Weilai-1216
C3008	Ningmaizi-1019	C3017	Yangfumai-8	C3026	CP03-28-1-1	C3035	Huamai-6	C3044	Kenong-9204	C3053	Sukemai-1
C3007	Shengxuan-6	C3016	Zhenmai-5	C3025	Yangfumai-5	C3034	Ningmai-13	C3043	Wanmai-108	C3052	CP20-39-11-1
C3006	Wanmai-52	C3015	Lvyu-098	C3024	P14	C3033	Sumai-8	C3042	CP01-39-3-204	C3051	CP02-9-4-8-2
C3005	Zhenmai-4	C3014	Zhenmai-09196	C3023	Dongmai-1301	C3032	Xumai-32	C3041	Ningmai-26	C3050	Baoji-0601
C3004	P124	C3013	Zhenmai-168	C3022	Tianmin-108	C3031	CP02-62-1-2-2-3F10	C3040	Sumai-3	C3049	Su-553
C3003	Zhenmai-12	C3012	CP20-39-11-2	C3021	Zhenmai-11	C3030	Zhen-9523	C3039	Huaimai-30	C3048	Zhenmai-10
C3002	Yangfumai-6	C3011	Pingmai-108	C3020	CP01-39-3-2-4	C3029	Guohong-9	C3038	Nannong-06Y86	C3047	Ningzimai-1
C3001	Yangmai-25	C3010	Yangmai-158	C3019	Aikang-58	C3028	CP02-8-5-5-21	C3037	Zhenmai-8	C3046	CP02-8-5-6-1
C2009	Pingmai-108	C2018	Yangmai-158	C2027	Yangmai-25	C2036	Nannong-06Y86	C2045	CP20-39-11-2	C2054	Sumai-8
C2008	Tianmin-108	C2017	Yangfumai-6	C2026	Yangfumai-8	C2035	Yangfumai-5	C2044	Zhenmai-09196	C2053	Huaimai-30
C2007	Ningmai-26	C2016	CP02-63-13-1	C2025	Zhenmai-5	C2034	CP01-39-17-1-3	C2043	Su-553	C2052	Zhenmai-168
C2006	Shengxuan-6	C2015	Lvyu-098	C2024	Zhenmai-8	C2033	Ningmai-13	C2042	Wanmai-52	C2051	Huamai-6
C2005	Aikang-58	C2014	CP01-39-3-204	C2023	Baoji-0601	C2032	Ningmaizi-1019	C2041	CP02-8-5-5-21	C2050	CP02-62-1-2-2-3F10
C2004	Wanyu-2	C2013	Sukemai-1	C2022	Zhenmai-12	C2031	P14	C2040	Guohong-9	C2049	Sumai-3
C2003	CP03-28-1-1	C2012	Yangmai-20	C2021	Wanmai-108	C2030	Xumai-32	C2039	Dongmai-1301	C2048	Zhenmai-11
C2002	CP01-39-3-2-4	C2011	CP20-39-11-1	C2020	Yangmai-16	C2029	CP02-9-4-8-2	C2038	Zhenmai-10	C2047	Kenong-9204
C2001	P124	C2010	Weilai-1216	C2019	Ningzimai-1	C2028	CP02-8-5-6-1	C2037	Zhenmai-4	C2046	Zhen-9523
C1009	Wanyu-2	C1018	Zhen-9523	C1027	P14	C1036	CP01-39-3-204	C1045	Ningmaizi-1019	C1054	Yangmai-158
C1008	Sukemai-1	C1017	Su-553	C1026	Zhenmai-8	C1035	Xumai-32	C1044	Ningmai-26	C1053	Yangmai-20
C1007	CP02-62-1-2-2-3F10	C1016	Yangmai-25	C1025	Yangmai-16	C1034	Zhenmai-12	C1043	Nannong-06Y86	C1052	CP01-39-17-1-3
C1006	P124	C1015	Baoji-0601	C1024	Shengxuan-6	C1033	CP03-28-1-1	C1042	CP01-39-3-2-4	C1051	Kenong-9204
C1005	Zhenmai-5	C1014	Ningzimai-1	C1023	Pingmai-108	C1032	CP02-9-4-8-2	C1041	Zhenmai-168	C1050	Zhenmai-09196
C1004	Wanmai-108	C1013	Ningmai-13	C1022	Aikang-58	C1031	Yangfumai-6	C1040	Lvyu-098	C1049	Zhenmai-4
C1003	Sumai-3	C1012	CP20-39-11-1	C1021	CP02-63-13-1	C1030	Dongmai-1301	C1039	Huaimai-30	C1048	CP02-8-5-5-21
C1002	Guohong-9	C1011	Zhenmai-11	C1020	Weilai-1216	C1029	CP02-8-5-6-1	C1038	Yangfumai-8	C1047	Sumai-8
C1001	Huamai-6	C1010	CP20-39-11-2	C1019	Zhenmai-10	C1028	Tianmin-108	C1037	Yangfumai-5	C1046	Wanmai-52

Table S9. Soil nutrient (0-25 cm soil layer) content before drilling in the 2019/2020 season.

pH	Organic matter (g/kg)	NO_3-N (mg/kg)	Phosphate (g/kg)	Potassium (g/kg)	Total organic N (mg/kg)	Plant available Phosphate (mg/kg)	Plant available Potassium (mg/kg)
6	24.2	1.35	0.61	13.2	10.4	3.04	160

Table S10. Processing time for three types of point cloud files at each analysis step.

Analysis steps	Processing time (backpack)	Display time (backpack)	Proc. time (UAV*)	Disp. time (UAV)	Proc. time (gantry)	Dis. time (gantry)
Step 1	2.5-3 minutes	35-45 seconds	1.5-2 mins	25-30 seconds	N/A	N/A
Step 2	1.5-2 mins	50-60 sec.	30-40 sec.	40-50 sec.	N/A	N/A
Step 3	10-15 sec.	0.2-0.5 sec.	30-40 sec.	0.5-1 sec.	10-15 sec.	0.3-0.5 sec.
Step 4	0.5-1.5 sec.	0.3-1 sec.	0.5-1 sec.	0.5-1 sec.	0.5-1.5 sec.	0.5-1 sec.
Step 5	30-40 sec.	0.5-1.5 sec.	20-25 sec.	0.5-1 sec.	5-10 sec.	0.5-1 sec.
Step 6	5-6 sec.	N/A	3-4 sec.	N/A	1-2 sec.	N/A
Step 7	5-10 sec. per plot	N/A	4-6 sec./plot	N/A	N/A	N/A

* Unmanned aerial vehicle

Supplemental Methods S1

The CropQuant-3D Software was developed using the Python GUI package, PyQt5, which allows the software application to be executed on different operating systems such as Windows and Mac OS. The step-by-step user guide detailed below is based on the Windows executable file.

1.1 Access the software

The software is running on Windows 10. Installation requires a user to download the zip file from the GitHub repository (https://github.com/The-Zhou-Lab/LiDAR/releases), unzip the file, and then run the software by double clicking the .exe executable (**Fig. S1.1**).

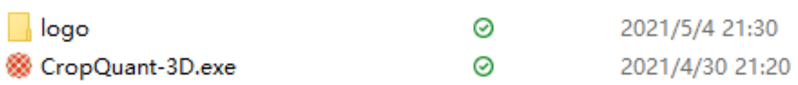

Figure S1.1 The icon of CropQuant-3D.exe

1.2 Software installation and execution

The initial interface of the software can be seen in **Figure S1.2** (left), where a user can enter input parameters to initiate point clouds processing, 3D trait analysis and results output. On the initial window, default input parameters have been prepopulated for the test LAS files downloadable from the GitHub repository. Users can change the parameters according to their analysis needs. Also, a brief introduction of these analysis steps and associated algorithms can be retrieved by clicking the information icons.

1.3 Pre-processing point clouds generated by the backpack LiDAR

In the Data Input section, users can click the 'Select file' button to select a LiDAR file collected by the backpack LiDAR for 3D trait analysis. This section automatically creates a result folder named with the date and time when the LiDAR file is analysed, where all intermediate results of the analysis workflow will be saved. The analysis workflow of the CropQuant-3D software is divided into 5 parts: data input, data pre-processing, plot segmentation, traits analysis, and data output (**Fig. S1.2**).

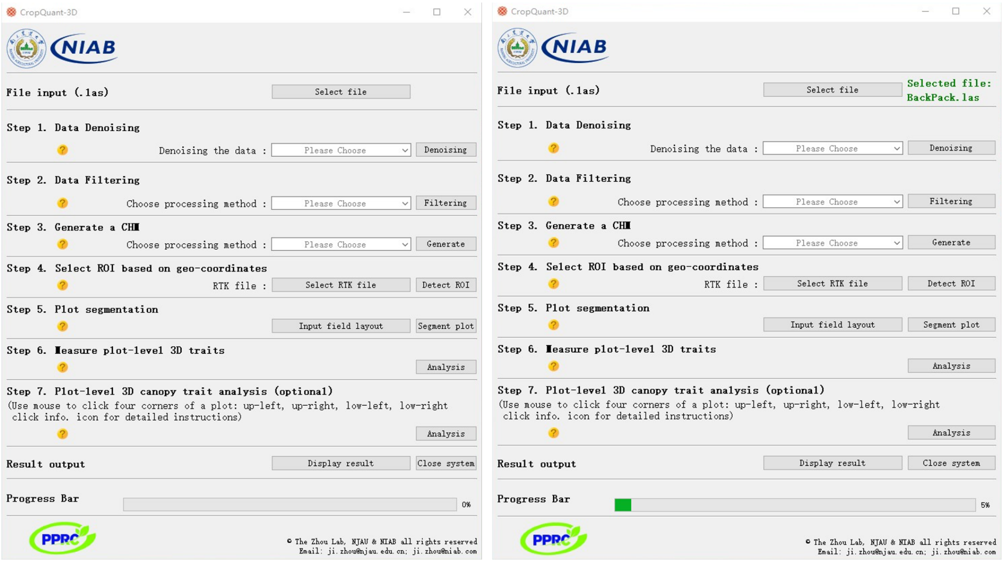

Figure S1.2 An initial window of the software and data input

1.3.1 Data pre-processing – denoising

In the data pre-processing section, users can perform denoising and filtering on the selected point cloud file (**Fig. S1.3**). To perform denoising, the user needs to input the required parameters N and K (default setting is 50 and 10), after that press the 'Denoising" button to initiate the denoising algorithm.

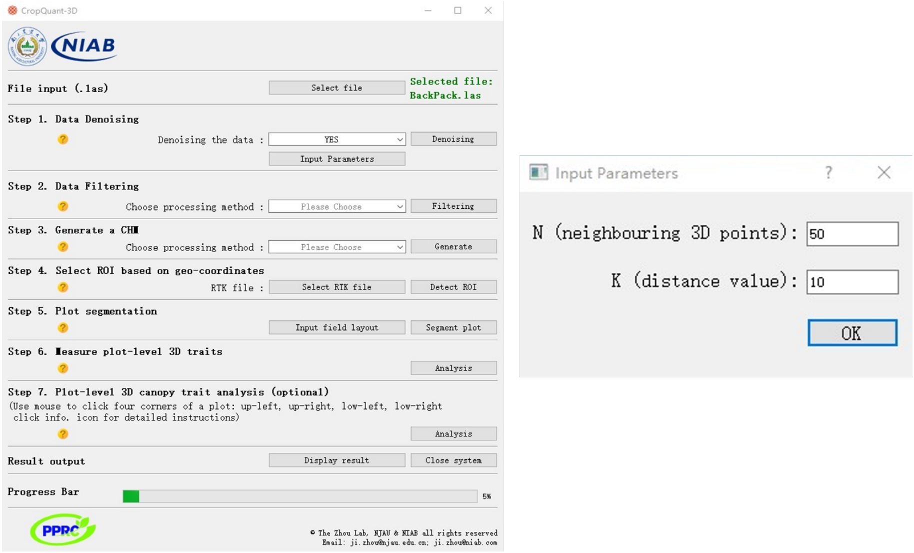

Figure S1.3 Point clouds denoising in *Step 1*

1.3.2 Data pre-processing – filtering

The filtering section is similar to the denoising process (**Fig. S1.4**). The users need to select "Slope based filtering" item from the dropdown list and then define the 'Radius' value (the default value is 2). the output of the filtering processing is a new LiDAR point cloud file.

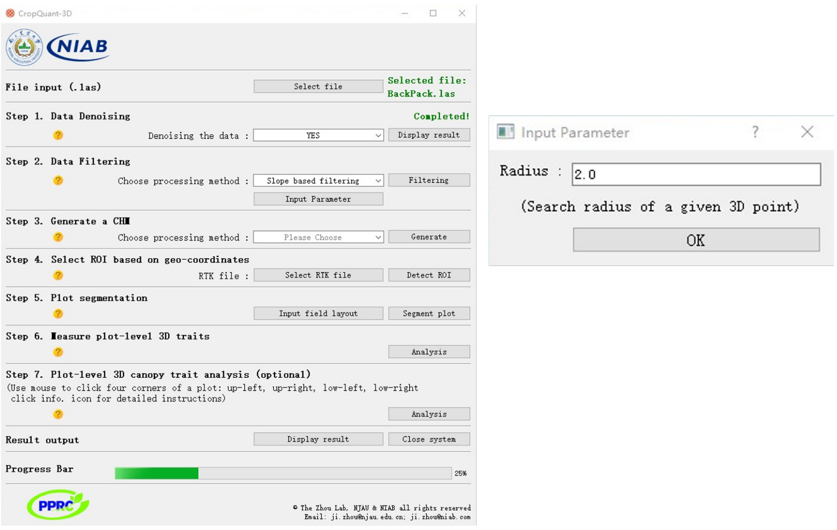

Figure S1.4 Point clouds filtering in *Step 2*

When a section is completed, we can click the 'Display result' button to visualise the newly generated point cloud file in 3D (**Fig. S1.5**).

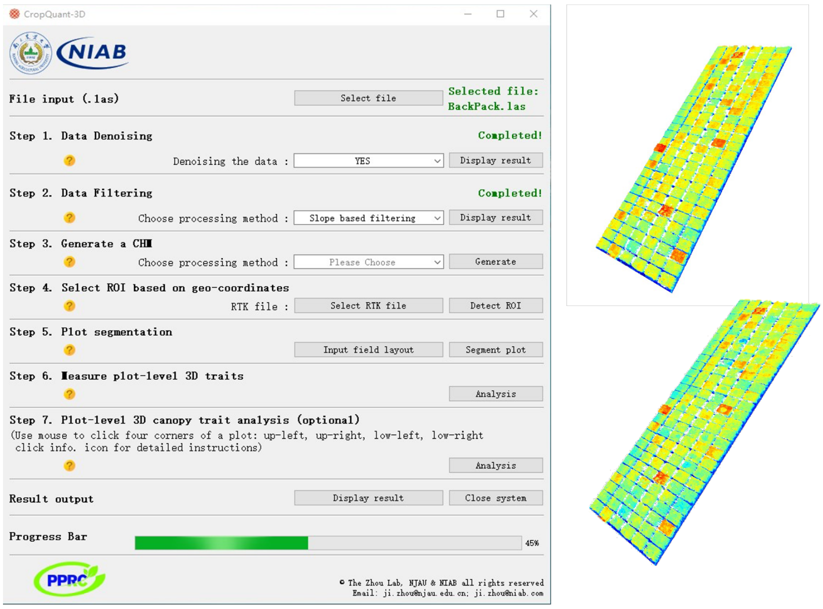

Figure S1.5 Intermediate results visualisation after denoising and filtering

1.3.3 Data pre-processing – generating CHM

To create a 2D CHM file from the processed point clouds in the third step (**Fig. S1.6**, left), users need to select the 'Tin gridding' algorithm from the processing method and then enter the input parameter 'Resolution' (defaulted to 1, where one stands for the exchange rate is 1 cm per pixel). Users need to press the 'Generate' button to create a 2D Canopy Height Model (CHM) image (**Fig. S1.6**, right).

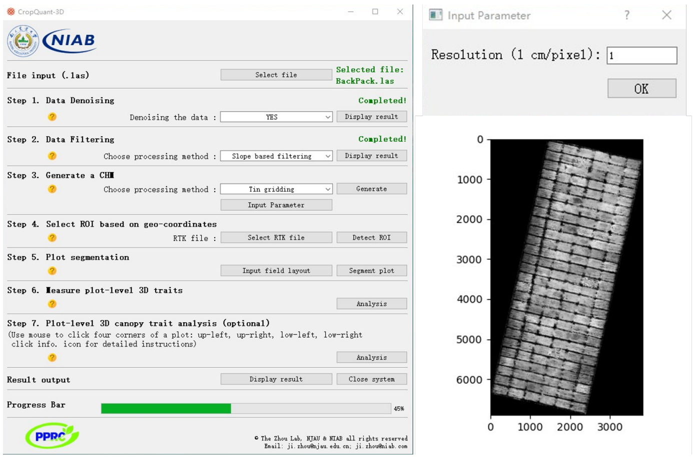

Figure S1.6 Generating a 2D CHM in *Step 3*

1.4 Pre-processing point clouds generated by the UAV-collected point clouds

In the data input section, users can click the 'Select file' button to select a UAV-SfM photogrammetry point cloud file for 3D trait analysis. Similar to the backpack LiDAR, this section automatically creates a result folder named with the date and time for this analysis.

1.4.1 Data pre-processing – denoising and filtering

To denoise UAV-generated point clouds, users can perform denoising by inputting the required parameters, after which pressing the 'Denoising' button to initiate the denoising processing. In the filtering section, the users need to select the "Cloth simulation filtering" algorithm. When the filtering is completed, we can click the 'Display result' button to display the generated point cloud file (**Fig. S1.7**, left), which will visualise results in 3D (**Fig. S1.7**, right).

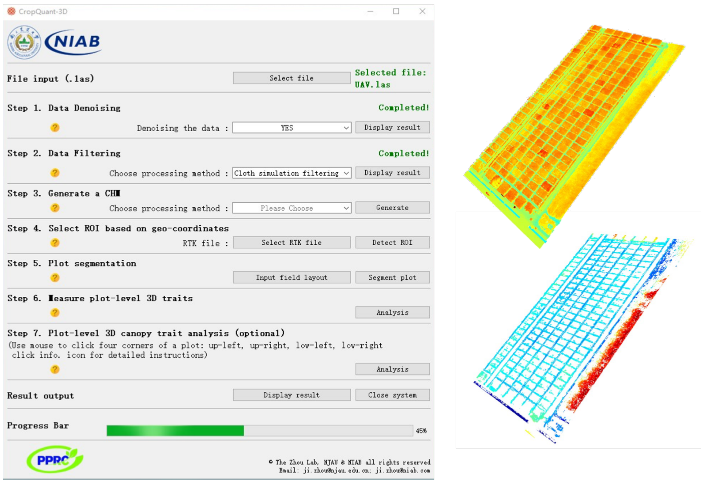

Figure S1.7 The visualisation of UAV-collected point clouds after denoising and filtering

1.4.2 Data pre-processing – generating CHM

To create a 2D CHM file from the point clouds in the third step (**Fig. S1.8**, left), the users need to select 'DSM-DTM' from the processing method and the click the input parameter to select a SHP field (shapefile) to define the geo-coordinates of the experimental field. Users need to press the 'Generate' button to create a 2D Canopy Height Model (CHM) image (**Fig. S1.8**, right).

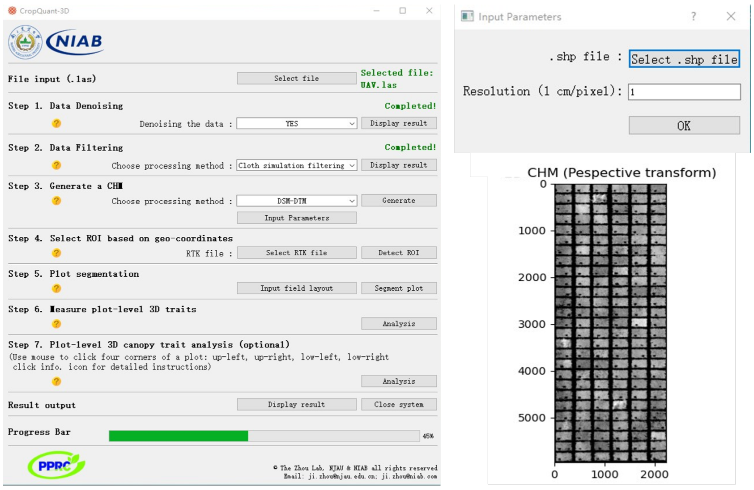

Figure S1.8 The generation of a 2D CHM using the UAV-collected point clouds

1.5 Pre-processing point clouds generated by the gantry LiDAR

Users can click the 'Select file' button to select a gantry LiDAR generated point cloud file for 3D trait analysis. In our work, we used FieldScan-acquired 3D point cloud data to demonstrate the 3D point processing procedure in CropQuant-3D.

1.5.1 Data pre-processing – denoising & filtering

Because most of the gantry system has integrated denoising and filtering methods, the generated point clouds normally do not need to go through denoising and filtering steps. Users can just select 'No' and 'Ground points removed' for both *Step 1* and *Step 2* (**Fig. S1.9**).

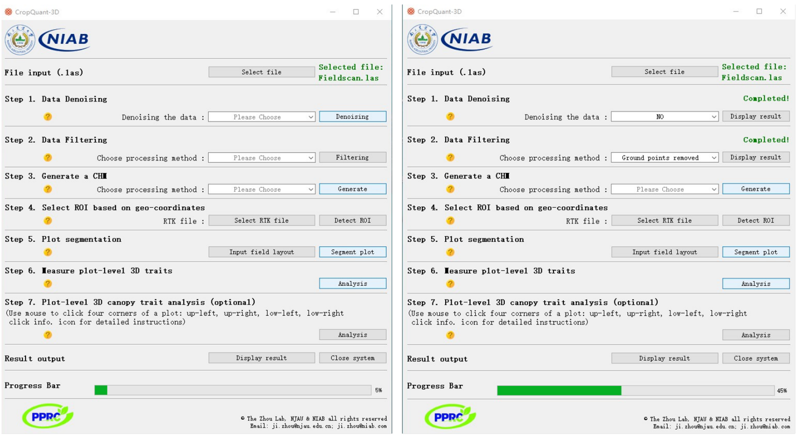

Figure S1.9 Denoising and filtering for point clouds generated by gantry LiDAR

1.5.2 Data pre-processing – display results

Users can click the ‘Display result’ button to display the newly processed point cloud file collected by the gantry LiDAR device in 3D (**Fig. S1.10**).

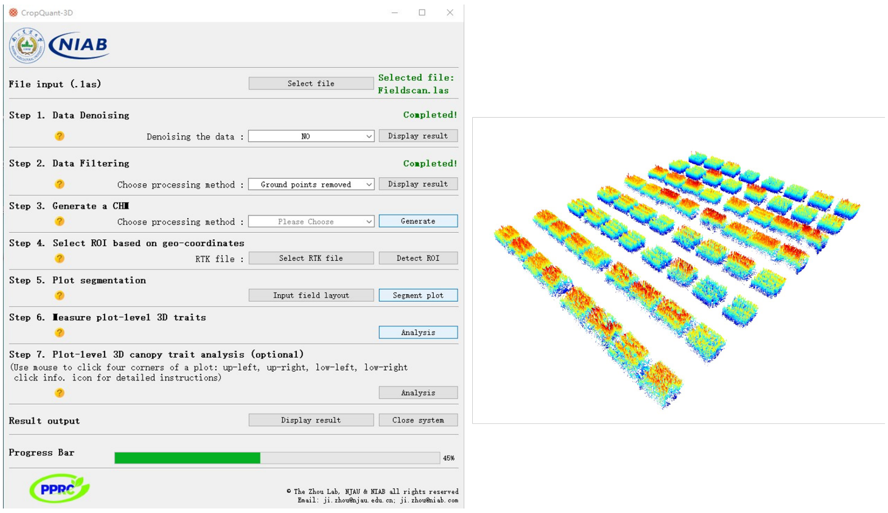

Figure S1.10 The visualisation of Gantry LiDAR collected point clouds

1.5.3 Data pre-processing – generating CHM

To create a 2D CHM file from the point clouds, the users need to select ‘Nearest neighbour gridding’ from the processing (**Fig. S1.11**, left). After pressing the ‘Generate’ button, a 2D Canopy Height Model (CHM) image will be created (**Fig. S1.11**, right).

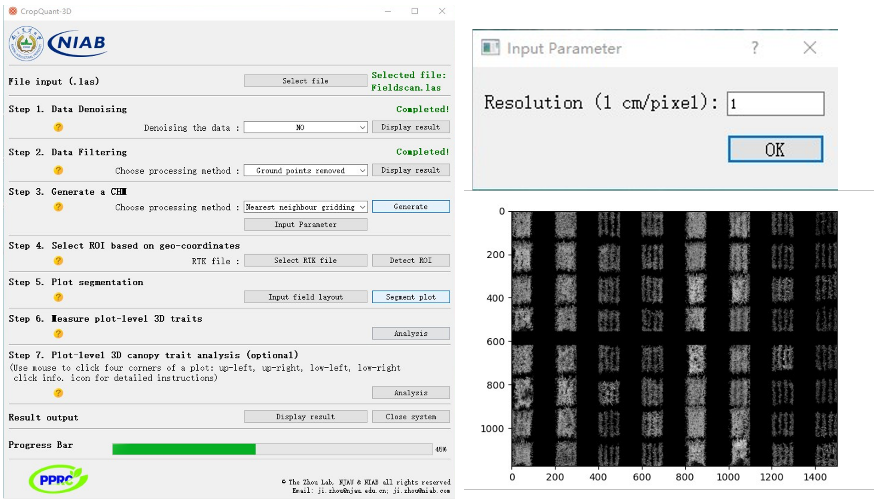

Figure S1.11 Generate CHM from the gantry LiDAR collected point clouds

1.6 Plot segmentation

For all types of input 3D point clouds, the 3D trait analysis steps are identical. Firstly, users need to create a 2D CHM file in the third step (**Fig. S1.12**). The default setting of the input parameter ‘Resolution’ is 1 (i.e. the exchange rate is 1 cm per pixel). Secondly, users need to press the ‘Generate’ button to create a 2D Canopy Height Model (CHM) image.

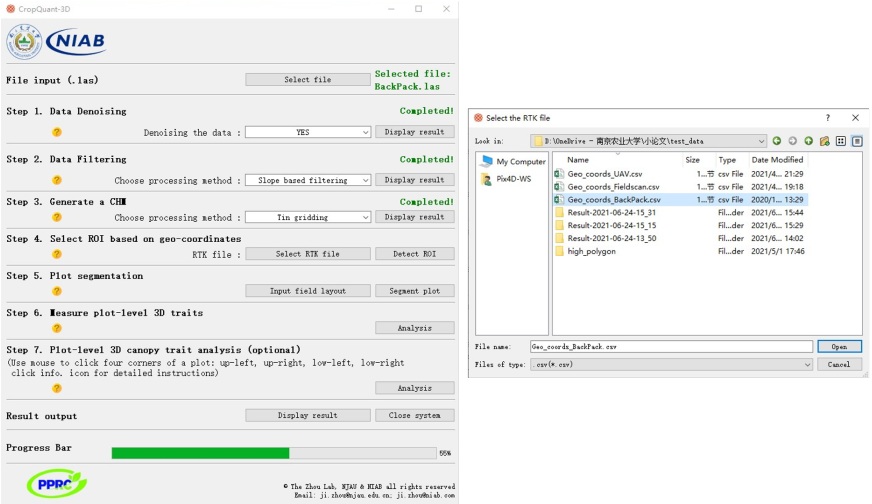

Figure S1.12 Plot segmentation procedure and its results

Then, users need to select regions of interest (ROI) to define the region for plot segmentation. Users are required to provide geo-coordinates, i.e. a RTK file. By clicking the 'Define ROI' button, the ROI of the experimental region can be defined. Finally, within the defined ROI, vertical and horizontal lines of the field can be detected by the CropQuant-3D. The default input parameters (i.e. Angle 1, Angle 2) are 360 and 30, which can be modified according to users' own CHM images. If the segmentation result misses some crop plots, users can add Input Paraments to provide optional inputs such as number of rows and columns in the field experiments (**Fig. S1.13**).

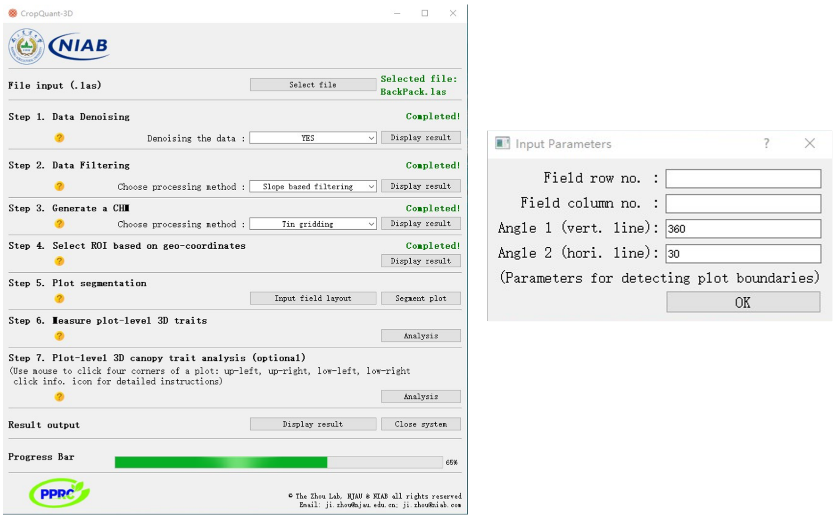

Figure S1.13 Plot segmentation procedure and its results

1.7 CHM and plot segmentation results of different types of point clouds

After the above steps, the results can be visualised by clicking the "Display result" button. The CHM images, ROIs, and plot segmentation results for point clouds collected through different data collection approaches can be displayed (**Figs. S1.14-16**).

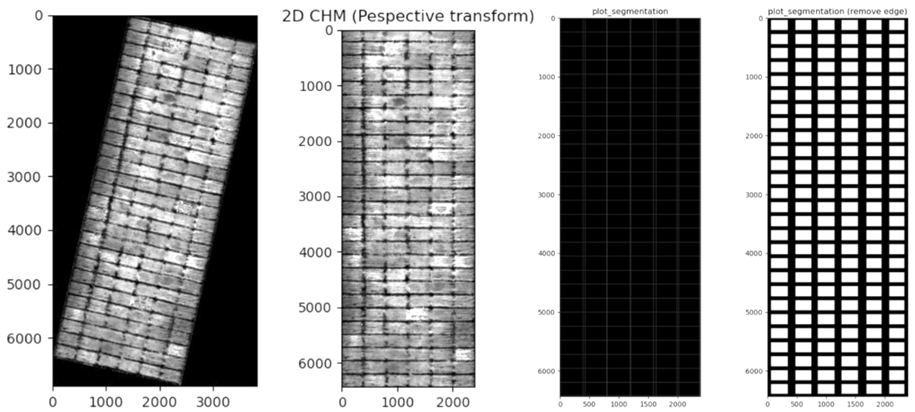

Figure S1.14 CHM, ROI and plot segmentation results for the backpack LiDAR

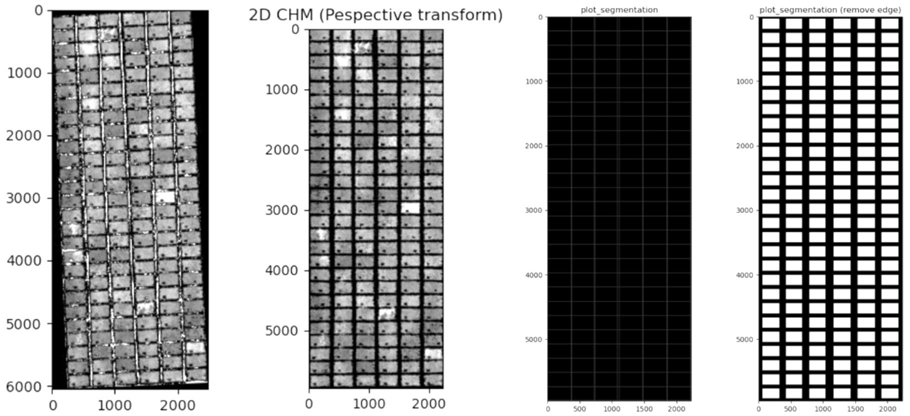

Figure S1.15 CHM, ROI and plot segmentation results for the UAV-SfM photogrammetry

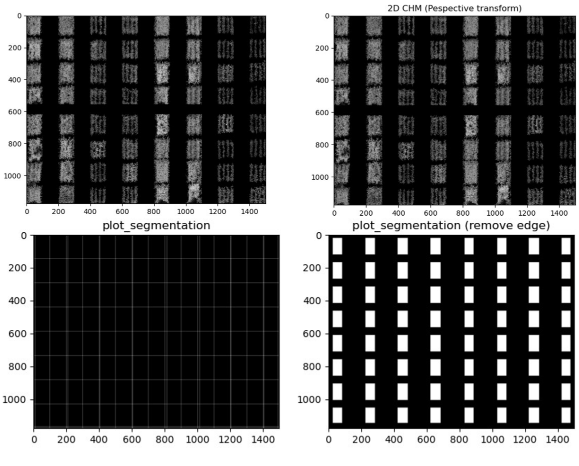

Figure S1.16 CHM, ROI and plot segmentation results for the gantry LiDAR

1.8 Trait Analysis

To analyse traits for all the plots, users just need to press the ‘Analysis’ button in the sixth step, which creates a new folder that contains images for all segmented plots as well as their plot-level traits such as height, canopy coverage, and canopy fluctuation in a .csv file. If users want to measure the plot-level

3D points, they shall click the ‘Analysis’ button and select plots in the pop-up window, following the order upper-left, upper-right, lower-left, to lower-right corners

1.9 Output and download results

After the analysis, users can click the ‘Download’ button to compress all output results in a zip file (**Fig. S1.17**). Also, users can open the result folder when the analysis is accomplished (all sections display green coloured “Completed1” working). The Close button will close the software.

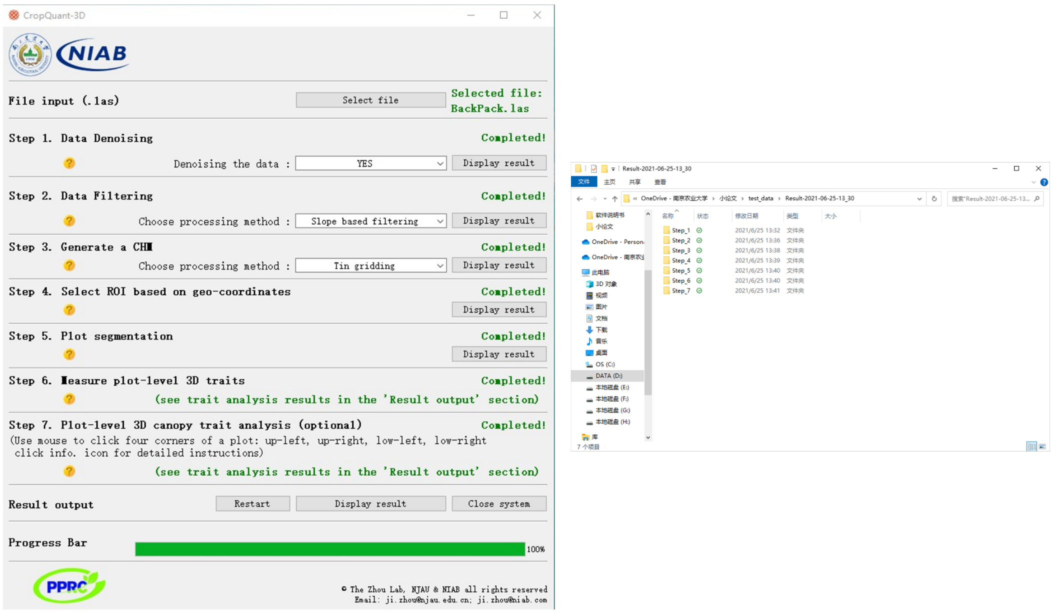

Figure S1.17 Results output and download

Supplemental Methods S2

Code fragments for pre-processing LiDAR data can be seen as follows:

```
# Import libraries
from laspy.file import File
from scipy import spatial
import whitebox
import osgeo.ogr as ogr
import osgeo.osr as osr
import rasterio as rio
import open3d as o3d

from skimage import io
import numpy as np
from matplotlib import pyplot as plt
import os
```

In [2]:

```
# Point cloud denosing, below are coded for demonstration purposes only
def denoising(input_dir,n,k):
```

```
    working_dir, filename = os.path.split(input_dir)
    # Read the point cloud
    inFile = File(input_dir, mode='r')
    points = inFile.points
    x,y,z = inFile.x,inFile.y,inFile.z

    # Packaging x,y,z
    lasdata = zip(x,y,z)
    tree = spatial.cKDTree(list(lasdata))
    # Conditional parameter
    sigma=n # Recommend:10
    K=k # Recommend:50
    k_dist=np.zeros_like(x)
    for i in range(len(x)):
        dist,index =tree.query(np.array([x[i],y[i],z[i]]), K)
        k_dist[i] = np.sum(dist)
    # The max distance of noise points
    max_distance = np.mean(k_dist) + sigma*np.std(k_dist)
    # The index
    outer_index=np.where(k_dist>max_distance)
    inner_index=np.where(k_dist<max_distance)
    print('outer points index array is:', outer_index)

    # Save points
    denoised_dir = os.path.join(working_dir, "denoising.las")
    outFile = File(denoised_dir,mode='w', header=inFile.header)
    outFile.points = points[inner_index]
    outFile.close()

    return(denoised_dir)
```

In [3]:

```
# Point cloud filtering
# radius: with a bigger radius value, the resolution will be better
# it will take longer time to process
def filtering(denoised_dir, radius):
    working_dir, filename = os.path.split(denoised_dir)
    filtered_dir = os.path.join(working_dir, 'filter.las')

    wbt = whitebox.WhiteboxTools()
    wbt.set_verbose_mode(False)
    # Sets verbose mode. If verbose mode is False, tools will not output messages

    inFile = File(denoised_dir, mode = "r")
    cls = inFile.classification
    if (cls[np.where(cls==2)].shape[0] > 0):
        wbt.height_above_ground(
        i=denoised_dir,
        output=filtered_dir)
    else:
        wbt.lidar_ground_point_filter(
            i=denoised_dir,
            output=filtered_dir,
            radius=radius,
            min_neighbours=0,
            slope_threshold=45.0,
            height_threshold=0.01,
            classify=True,
```

```
            slope_norm=True,
            height_above_ground=True)

    return(filtered_dir)
```

In [4]:

```
# 2D CHM generation
def CHM(filtered_dir,resolution):
    working_dir, filename = os.path.split(filtered_dir)
    CHM_dir = os.path.join(working_dir, 'CHM.tif')
    wbt = whitebox.WhiteboxTools()
    wbt.set_verbose_mode(False)

    #Generating a 2D CHM and save it.
    wbt.lidar_tin_gridding(
        i=filtered_dir,
        output=CHM_dir,
        parameter="elevation",
        returns="all",
        resolution=resolution,
        exclude_cls=None,
        minz=None,
        maxz=None,
        max_triangle_edge_length=None)

    return(CHM_dir)
```

In [5]:

```
# Export to a new point cloud file
def Write_to_pcd(path):
    inFile = File(path, mode='r')
    x,y,z = inFile.x,inFile.y,inFile.z
    # Remove offset.
    x_offset = x - np.min(x)
    y_offset = y - np.min(y)
    z_offset = z - np.min(z)
    points = zip(x_offset,y_offset,z_offset)
    # Write to pcd.
    pcd = o3d.geometry.PointCloud()
    pcd.points = o3d.utility.Vector3dVector(points)

    return(pcd)
```

In [6]:

```
# Point cloud visualization using Open3D
def custom_draw_geometry_with_key_callback(pcd):

    # Create a path to save a screen capture.
    root_dir = os.path.join(os.path.expanduser("~"), 'Desktop')
    save_path = os.path.join(root_dir, 'Screenshot.png')

    # Change the background.
    def change_background_to_black(vis):
        opt = vis.get_render_option()
        opt.background_color = np.asarray([0, 0, 0])
        return False

    def capture_depth(vis):
        depth = vis.capture_depth_float_buffer()
        plt.imshow(np.asarray(depth))
```

```
        plt.show()
        return False

    # Save image.
    def capture_image(vis):
        image = vis.capture_screen_float_buffer()
        fig,ax = plt.subplots(figsize=(20,20))
        ax.imshow(image)
        plt.savefig(save_path)
        return False

    key_to_callback = {}
    key_to_callback[ord("K")] = change_background_to_black
    key_to_callback[ord(",")] = capture_depth
    key_to_callback[ord(".")] = capture_image
    o3d.visualization.draw_geometries_with_key_callbacks([pcd], key_to_callback)
```

In [7]:

```
# Display 2D CHM after 3D points processing
def CHM_Visualization(path):
    img = io.imread(path)
    img = np.array(img*255,dtype='uint8')

    fig, axes = plt.subplots(1, 2, figsize=(10, 10))

    axes[0].imshow(img, cmap=plt.cm.gray)
    axes[0].set_title('img')
    axes[1].imshow(img, cmap=plt.cm.jet)
    axes[1].set_title('img(jet)')

#   fig,ax = plt.subplots(figsize=(10,10))
#   ax.imshow(img, cmap=plt.cm.gray)
```

In [8]:

```
# The following is the main function for point cloud processing
# Step 1: Point Cloud Denosing
input_dir = r"C:\Users\Pix4D-WS\Desktop\test\test.las"
denoised_dir = denoising(input_dir,10,50)
print(denoised_dir)
outer points index array is: (array([13599, 18383, 30593, ..., 15090550, 15090551],
      dtype=int64),)
C:\Users\Pix4D-WS\Desktop\test\denoising.las
```

In [9]:

```
# Step 2: Point Cloud Filtering.
filtered_dir = filtering(denoised_dir, 2.0)
print(filtered_dir)
C:\Users\Pix4D-WS\Desktop\test\filter.las
```

In [10]:

```
# Step 3: 2D Canopy Height Model, CHM.
CHM_dir = CHM(filtered_dir,0.01)
print(CHM_dir)
C:\Users\Pix4D-WS\Desktop\test\CHM.tif
```

In [11]:

```
# 3D visualization for Step 1.
Denoised_pcd = Write_to_pcd(denoised_dir)
custom_draw_geometry_with_key_callback(Denoised_pcd)
```

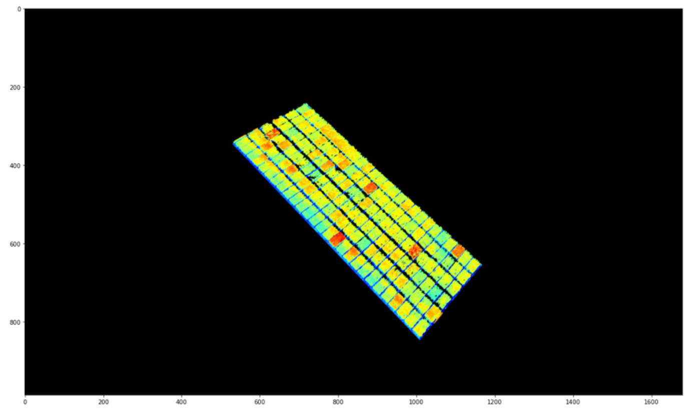

In [12]:

```
# 3D visualization for Step 2.
Filtered_pcd = Write_to_pcd(filtered_dir)

custom_draw_geometry_with_key_callback(Filtered_pcd)
```

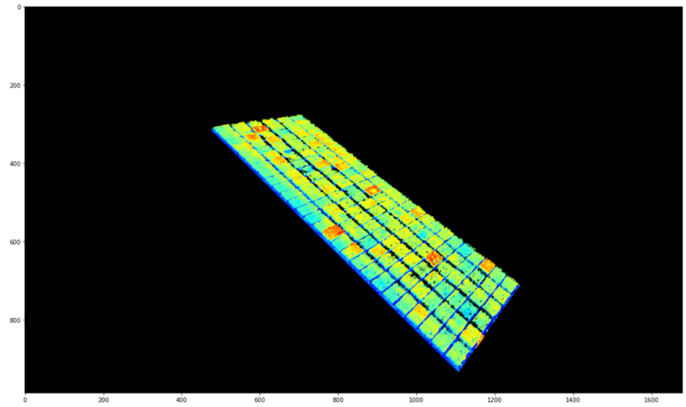

In [13]:

```
# Visualization for Step 3.
CHM_Visualization(CHM_dir)
```

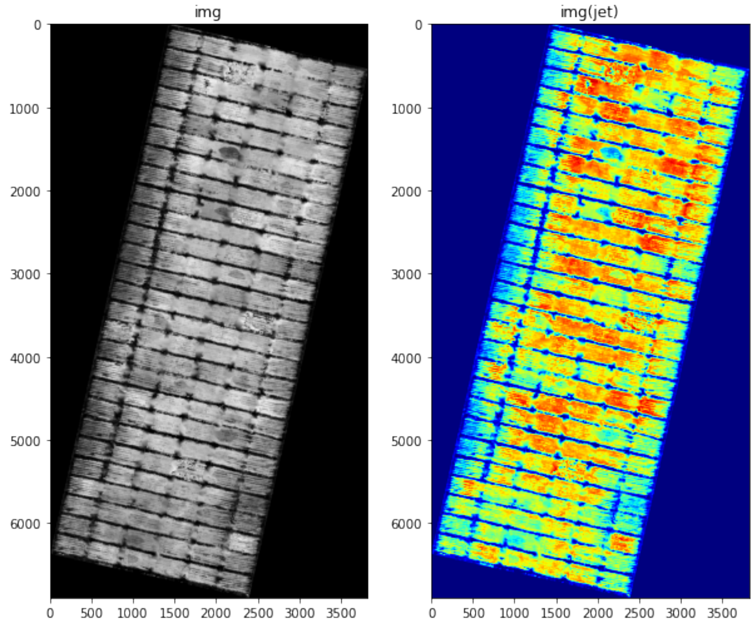

In [1]:

```
# End of Data pre-processing
```

Supplemental Methods S3

Code fragments for segmenting plots based on LiDAR data can be seen as follows:

```
# Import libraries
import whitebox
import osgeo.ogr as ogr
import osgeo.osr as osr
import rasterio as rio

from skimage import io
from skimage.filters import gaussian, threshold_sauvola, threshold_local
from skimage.morphology import
remove_small_holes,erosion,disk,dilation,remove_small_objects
from skimage.filters import sobel_v, sobel_h
from skimage.transform import hough_line, hough_line_peaks, resize
from skimage.draw import line_aa
from skimage.measure import label, regionprops

from matplotlib import pyplot as plt
import pandas as pd
import numpy as np
import cv2
import math
import os
```

In [2]:

```
# Geo coordinates to Pixel coordinates.
# (The order of coordinates:up_left,up_right,low_left,low_right.)
# Perspective Transform.(Note：scikit-image(r,c),opencv(c,r))
def Extract_roi(path,RTK_filepath):
    # Read Geo coords
    Geo_data = pd.read_csv(RTK_filepath,header=None)
    up_left = (Geo_data.iloc[0,2], Geo_data.iloc[0,3])
    up_right = (Geo_data.iloc[1,2], Geo_data.iloc[1,3])
    low_left = (Geo_data.iloc[2,2], Geo_data.iloc[2,3])
    low_right = (Geo_data.iloc[3,2], Geo_data.iloc[3,3])
    # Coordinates Transform.
    # Read GeoTiff
    CHM = rio.open(path,driver="GTiff")
    # Geo coordinates array.
    Geo_coordinates = []
    Geo_coordinates.append(up_left)
    Geo_coordinates.append(up_right)
    Geo_coordinates.append(low_left)
    Geo_coordinates.append(low_right)
    # Pixel coordinates array.
    pixel_coordinates = []
    for n in np.arange(4):
        x, y = Geo_coordinates[n]
        row, col = CHM.index(x, y)
        pixel_coordinates.append((row, col))
    print('pixel_coordinates are:',pixel_coordinates)

    # Perspective Transform.
    working_dir,filename = os.path.split(path)
```

```
    # Read file
    img = io.imread(path)
    # Calculate the values of row and column.
    columns_1 = np.int(math.sqrt((pixel_coordinates[0][0]-
pixel_coordinates[1][0])**2 + (pixel_coordinates[0][1]-
pixel_coordinates[1][1])**2))
    columns_2 = np.int(math.sqrt((pixel_coordinates[2][0]-
pixel_coordinates[3][0])**2 + (pixel_coordinates[2][1]-
pixel_coordinates[3][1])**2))
    columns = np.max((columns_1,columns_2))

    rows_1 = np.int(math.sqrt((pixel_coordinates[0][0]-
    pixel_coordinates[2][0])**2 + (pixel_coordinates[0][1]-
    pixel_coordinates[2][1])**2))
    rows_2 = np.int(math.sqrt((pixel_coordinates[1][0]-
    pixel_coordinates[3][0])**2 + (pixel_coordinates[1][1]-
    pixel_coordinates[3][1])**2))
    rows = np.max((rows_1,rows_2))
    # Start Transformation.
    pts1 = np.float32([[pixel_coordinates[0][1],pixel_coordinates[0][0]],
                       [pixel_coordinates[1][1],pixel_coordinates[1][0]],
                       [pixel_coordinates[2][1],pixel_coordinates[2][0]],
                       [pixel_coordinates[3][1],pixel_coordinates[3][0]]])
    pts2 = np.float32([[0,0], [columns,0], [0,rows], [columns, rows]])
    # Transfer matrix.
    Transform_Array = cv2.getPerspectiveTransform(pts1, pts2)
    # Transfer the image.
    Transformed_img = cv2.warpPerspective(img, Transform_Array,(columns, rows))

    return(Transformed_img)
```

In [3]:

```
# Horizontal & vertical edges detection.
def Edges_detection(Transformed_img):
    Transformed_img1 = np.array(Transformed_img*255)
    Transformed_img = np.array(Transformed_img*255,dtype='uint8')

    # Gaussian filtering to remove interference.
    Transformed_img_gau = gaussian(Transformed_img, sigma=5)
    # Horizontal edge mask.
    threshold_mask = Transformed_img_gau > threshold_sauvola(Transformed_img_gau,
window_size=175, k=0.2)
    edges_h = dilation(sobel_h(threshold_mask),disk(6))
    # Vertical edge mask.
    Transformed_img_gau1 = gaussian(Transformed_img1, sigma=5)
    threshold_mask1 = Transformed_img_gau1 > threshold_local(Transformed_img_gau1,
195, 'gaussian')
    mask_v = erosion(remove_small_holes(threshold_mask1,2750),disk(5))
    edges_v = dilation(sobel_v(mask_v),disk(6))

    return(edges_h,edges_v)
```

In [4]:

```
def Plot_Mask(edges_h, edges_v, p1, p2):
    # Vertical direction.
    tested_angles = np.linspace(-np.pi/2, np.pi/2, p1)
    h, theta, d = hough_line(edges_v, theta=tested_angles)
    # Horizontal  direction.
    tested_angles1 = np.linspace(-np.pi/2, np.pi/2, p2)
```

```
h1, theta1, d1 = hough_line(edges_h, theta=tested_angles1)

# Creat grid image.
blank_img = np.zeros((Transformed_img.shape[0],Transformed_img.shape[1]))
Grid_image = blank_img.copy()
tmp_line_img = blank_img.copy()

height = Transformed_img.shape[0]
width = Transformed_img.shape[1]

# Vertical direction.
hspace, angles, distances = hough_line_peaks(h, theta, d)
distances_v = []
for angle, dist in zip(angles, distances):
    # Filter out vertical lines.
    if (np.cos(angle)>0.99999):
        distance_v = np.abs(dist)
        distances_v.append(distance_v)
# Filter out lines that are too close.
distances_v = np.hstack((distances_v,Transformed_img.shape[1]))
distances_v = np.sort(np.hstack((distances_v,10)))
D_value = np.diff(distances_v)
distances_v_refined = []
for i in range(distances_v.shape[0]-1):
    if distances_v[i+1]-distances_v[i] < np.max(D_value)*0.75:
        distances_v[i+1] = distances_v[i]
    elif distances_v[i+1]-distances_v[i] >= np.max(D_value)*0.75:
        distances_v_refined.append(distances_v[i])
distances_v_refined.append(Transformed_img.shape[1]-10)
num = np.arange(len(distances_v_refined))
for n, dist_v in zip(num, distances_v_refined):
    rr, cc, val = line_aa(0, np.int(dist_v)-1, height-1, np.int(dist_v)-1)
    tmp_line_img[rr, cc] = val * 1
    Grid_image = np.logical_or(Grid_image,tmp_line_img)

# Horizontal  direction.
hspace1, angles1, distances1 = hough_line_peaks(h1, theta1, d1)
distances_h = []
for angle1, dist1 in zip(angles1, distances1):
    # Filter out horizontal lines.
    if (np.cos(angle1)<0.1):
        distance_h = np.abs(dist1)
        distances_h.append(distance_h)
# Filter out lines that are too close.
distances_h = np.hstack((distances_h,Transformed_img.shape[0]))
distances_h = np.sort(np.hstack((distances_h,10)))
D_value1 = np.diff(distances_h)
distances_h_refined = []
for i in range(distances_h.shape[0]-1):
    if distances_h[i+1]-distances_h[i] < np.max(D_value1)*0.8:
        distances_h[i+1] = distances_h[i]
    elif distances_h[i+1]-distances_h[i] >= np.max(D_value1)*0.8:
        distances_h_refined.append(distances_h[i])
distances_h_refined.append(Transformed_img.shape[0]-10)
num1 = np.arange(len(distances_h_refined))
for n1, dist_h in zip(num1, distances_h_refined):
    rr, cc, val = line_aa(np.int(dist_h)-1, 0, np.int(dist_h)-1, width-1)
```

```
        tmp_line_img[rr, cc] = val * 1
        Grid_image = np.logical_or(Grid_image,tmp_line_img)

    Grid_Image = dilation(Grid_image, disk(7))
    Grid_Segment = np.logical_not(Grid_Image)
    Grid_Segment_Refine = erosion(Grid_Segment, disk(3))
    # remove minimal area.
    minimal_area = int(np.max(D_value)*np.max(D_value1)*0.2)
    Grid_Segment_Refine = remove_small_objects(Grid_Segment_Refine, minimal_area)

    label_img = label(Grid_Segment_Refine)
    mask = np.zeros((Grid_Segment_Refine.shape[0],Grid_Segment_Refine.shape[1]))

    for region in regionprops(label_img):
        minr, minc, maxr, maxc = region.bbox

        roi=Grid_Segment_Refine[int(region.centroid[0])-
         80:int(region.centroid[0])+80, int(region.centroid[1])-
         130:int(region.centroid[1])+130]

        cv2.rectangle(mask, (int(region.centroid[1])-130,
         int(region.centroid[0])-80),
         (int(region.centroid[1])+130,int(region.centroid[0])+80), 255, -1)
    return(Grid_Segment_Refine,mask)
```

In [5]:

```
def Segment_ROI(Grid_Segment_Refine,Transformed_img):
    # Creat a folder for save images.
    folder_path = os.path.join(results_folder, 'plotimages')
    i = 0
    try:
        os.mkdir(folder_path)
    except FileExistsError:
        # Judge folder existence.
        while(os.path.exists(folder_path)):
            i=i+1
            folder_path = os.path.join(results_folder, 'plotimages'+str(i))
        # Creat a folder
        os.mkdir(folder_path)

    # Calculate Heights.
    Labelled_Grid_Img = label(Grid_Segment_Refine)
    Centroid_coordinates = []
    for region in regionprops(Labelled_Grid_Img):
        # Getting centroid coordinates.
        Centroid_coordinate = region.centroid
        Centroid_coordinates.append(Centroid_coordinate)
    # Calculate the rows and columns of planting area.
    rows = np.unique(np.array(Centroid_coordinates)[:,0]).shape[0]
    columns = int(len(Centroid_coordinates)/rows)
    # Each wheat plot:(h,w).
    region_h = int((Transformed_img.shape[0]/rows)*0.8)
    region_w = int((Transformed_img.shape[1]/columns)*0.75)
    # Heights.
    weighted_centroid_coords = []
    wheat_mean_heights = []
    for n,region in enumerate(regionprops(Labelled_Grid_Img,Transformed_img)):
        # Getting weighted centroid coordinates.
```

```python
        weighted_centroid_coord = region.weighted_centroid
        weighted_centroid_coords.append(weighted_centroid_coord)
        # Region area.
        minr = int(weighted_centroid_coord[0] - region_h/2)
        maxr = int(weighted_centroid_coord[0] + region_h/2)
        minc = int(weighted_centroid_coord[1] - region_w/2)
        maxc = int(weighted_centroid_coord[1] + region_w/2)
        roi = Transformed_img[minr:maxr,minc:maxc]
        # This is the region image.
        plot_image = np.array(roi*255,dtype='uint8')
        save_path = os.path.join(folder_path,'plot_'+str(n+1)+'.png')
        io.imsave(save_path,plot_image)
        # Remove outliers.
        roi = roi[roi < 2]
        # Calculate the appropriate height.
        roi_wheat_height = roi[roi > np.percentile(roi,90)]
        # mean
        wheat_mean_height = round(np.mean(roi_wheat_height)*100, 2)
        wheat_mean_heights.append(wheat_mean_height)

    # rows&columns
    row_index=[]
    column_index=[]
    for i in range(int(rows)):
        for j in range(int(columns)):
            row_index.append(i+1)
            column_index.append(j+1)

    # Write to csv files.
    # Plot traits.
    dt = pd.DataFrame({'row_id':row_index,'column_id':column_index,'Heights':
    wheat_mean_heights})
    save_path = os.path.join(results_folder,'Heights.csv')
    dt.to_csv(save_path,encoding="gbk")

    return(folder_path)
```

In [6]:

```python
# Choose interested varieties of wheat.
# The order of choosing:up_left,up_right,low_left,low_right.
# event-Operational response event；(x,y)-pixel coordinates；
# flags-The event of the Mouse；param-A function pointer.
def Segment_point_cloud(img_path, las_path):
    ### Choose regions of interest plots!
    ### Each plot:up_left,up_right,low_left,low_right.
    img = io.imread(img_path)
    img = np.array(img*255,dtype='uint8')
    img = cv2.resize(img,None, fx=0.15, fy=0.15)
    pixel_coords = []
    def OnMouseAction(event, x, y, flags, param):
        # Mouse Action
        if event == cv2.EVENT_LBUTTONDOWN:
            xy = "%d,%d" % (x, y)
            print(xy)
            pixel_coords.append(np.array((y,x)))
            cv2.circle(img, (x, y), 3, (0, 0, 255), thickness = -1)
            cv2.imshow('image', img)
```

```
        return(pixel_coords)
    # 'Enter' to finish the event!!!
    cv2.namedWindow('image')
    cv2.setMouseCallback('image', OnMouseAction)
    cv2.imshow('image', img)
    cv2.waitKey(0)
    cv2.destroyAllWindows()
    ### Creat a folder to save wheat plot point cloud.
    folder_path = os.path.join(results_folder, 'plot_point_cloud')
    i = 0
    try:
        os.mkdir(folder_path)
    except FileExistsError:
        # Judge folder existence.
        while(os.path.exists(folder_path)):
            i=i+1
            folder_path = os.path.join(results_folder, 'plot_point_cloud_'+str(i))
        # Creat a folder
        os.mkdir(folder_path)

    ### This step is to begin to segment point cloud.
    CHM = rio.open(img_path,driver="GTiff")
    # Transform Matrix
    transform = CHM.transform
    wbt = whitebox.WhiteboxTools()
    working_dir, filename = os.path.split(las_path)
    wbt.set_working_dir(working_dir)
    wbt.set_verbose_mode(False)
    for i in range(0,int(len(pixel_coords)/4)):
        pixel_coords1 = np.array(pixel_coords[4*i:4*(i+1)])/0.15
        # Geo coords
        up_left = transform * np.array((pixel_coords1[0][1],pixel_coords1[0][0]))
        up_right = transform * np.array((pixel_coords1[1][1],pixel_coords1[1][0]))
        low_left = transform * np.array((pixel_coords1[2][1],pixel_coords1[2][0]))
        low_right = transform * np.array((pixel_coords1[3][1],pixel_coords1[3][0]))
        geo_coords = np.array((up_left,up_right,low_left,low_right))
        # ESRI( Shapefile),and save it.
        driver = ogr.GetDriverByName("ESRI Shapefile")
        data_source = driver.CreateDataSource(os.path.join(working_dir,
'polygon.shp'))
        # Creat spatial reference,EPSG Encode(https://epsg.io/)
        srs = osr.SpatialReference()
        srs.ImportFromEPSG(28356)  #Robin LiDAR:GDA94/56,that is 28356.
        #Create a layer with the same name as the registered driver SHP
above,Feature Type:MultiPolygon.
        layer = data_source.CreateLayer("polygon.shp", srs, ogr.wkbMultiPolygon)
        feature = ogr.Feature(layer.GetLayerDefn())
        wkt = 'polygon((%f %f,%f %f,%f %f,%f %f,%f %f))' % (

geo_coords[0][0],geo_coords[0][1],geo_coords[2][0],geo_coords[2][1],geo_coords[3][0
],geo_coords[3][1],geo_coords[1][0],
            geo_coords[1][1],geo_coords[0][0],geo_coords[0][1])
        print(wkt)
        polygon = ogr.CreateGeometryFromWkt(wkt)
        feature.SetGeometry(polygon)
        #Creat feature(shapefile).
```

```
        layer.CreateFeature(feature)
        feature = None
        data_source = None
        wbt.clip_lidar_to_polygon(
            i=filename,
            polygons='polygon.shp',
            output=os.path.join(folder_path,'plot_'+str(i+1)+'.las')
        )
    return(folder_path)
```

In [7]:

```
# Related file path
CHM_dir = r"C:\Users\Pix4D-WS\Desktop\test\CHM.tif"
Geo_coords = r"C:\Users\Pix4D-WS\Desktop\test\Geo_coords.csv"
filtered_dir = r"C:\Users\Pix4D-WS\Desktop\test\filter.las"

# Step 1: Create a folder to save results.
root, filename = os.path.split(filtered_dir)
results_folder = os.path.join(root, 'results')
i = 0
try:
    os.mkdir(results_folder)
except FileExistsError:
    # Judge folder existence.
    while(os.path.exists(results_folder)):
        i=i+1
        results_folder = os.path.join(root, 'results'+str(i))
    # Creat a folder
    os.mkdir(results_folder)
```

In [8]:

```
# Step 2: Get the ROI
Transformed_img = Extract_roi(CHM_dir,Geo_coords)

# Save(tif)
Tif_path = os.path.join(results_folder,'Transformed_img.tif')
cv2.imwrite(Tif_path,Transformed_img)
# Save(jpg)
Jpg_path = os.path.join(results_folder,'Transformed_img.jpg')
cv2.imwrite(Jpg_path,Transformed_img*255)
pixel_coordinates are: [(57, 1451), (571, 3785), (6330, 35), (6846, 2366)]
```

Out[8]:

```
True
```

In [9]:

```
# Step 3: Detect Horizontal&Vertical edges
edges_h,edges_v = Edges_detection(Transformed_img)

# Step 4: Create a mask for segmentation.
# It will generate a segmented mask which is saved in the results folder.
Plot_Segment_Refine,mask = Plot_Mask(edges_h, edges_v, 360, 30)
# Save the mask.
cv2.imwrite(os.path.join(results_folder,
'Plot_Segment.jpg'),Plot_Segment_Refine*255)
```

Out[9]:

```
True
```

In [10]:

```
# Step 5: Segment the ROI and save the data of wheat heights.
images_folder = Segment_ROI(Plot_Segment_Refine,Transformed_img)
len(os.listdir(images_folder))
```

Out[10]:

```
162
```

In [11]:

```
# Step 6: Segment plot point cloud.
plot_pointcloud_folder = Segment_point_cloud(CHM_dir, filtered_dir)


len(os.listdir(plot_pointcloud_folder))
141,363
189,376
135,387
178,399
127,429
175,446
121,457
163,469
106,537
147,549
98,564
144,573
polygon((-2161363.780000 13894348.580000,-2161364.180000 13894346.980000,-2161361.3
13333 13894346.180000,-2161360.580000 13894347.713333,-2161363.780000 13894348.5800
00))
polygon((-2161364.713333 13894344.180000,-2161365.113333 13894342.313333,-2161362.3
13333 13894341.513333,-2161361.513333 13894343.046667,-2161364.713333 13894344.1800
00))
polygon((-2161366.113333 13894336.980000,-2161366.646667 13894335.180000,-2161363.5
80000 13894334.580000,-2161363.380000 13894336.180000,-2161366.113333 13894336.9800
00))
```

Out[11]:

```
3
```

In [12]:

```
# Visualization of Step 1.
img = np.array(Transformed_img*255,dtype='uint8')
fig, axes = plt.subplots(1, 2, figsize=(10, 10))
axes[0].imshow(img, cmap=plt.cm.gray)
axes[0].set_title('img(gray)')
axes[1].imshow(img, cmap=plt.cm.jet)
axes[1].set_title('img(jet)')
```

Out[12]:

```
Text(0.5, 1.0, 'img(jet)')
```

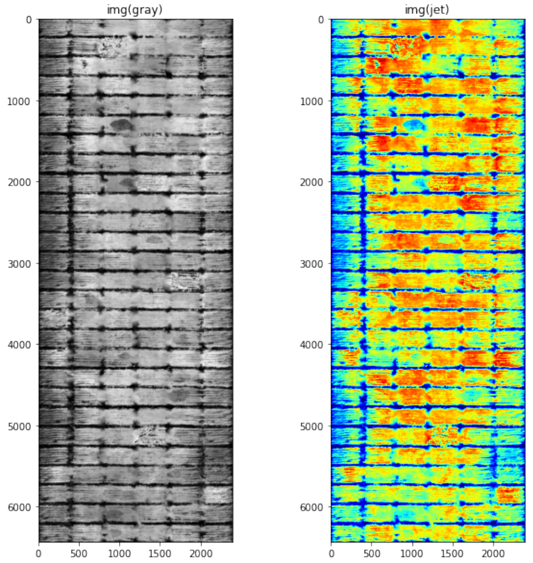

In [13]:

```
# Visualization of Step 2.
fig, axes = plt.subplots(1, 2, figsize=(10, 10))
```

```
axes[0].imshow(edges_h, cmap=plt.cm.gray)
axes[0].set_title('edges_h')
axes[1].imshow(edges_v, cmap=plt.cm.gray)
axes[1].set_title('edges_v')
```

Out[13]:

```
Text(0.5, 1.0, 'edges_v')
```

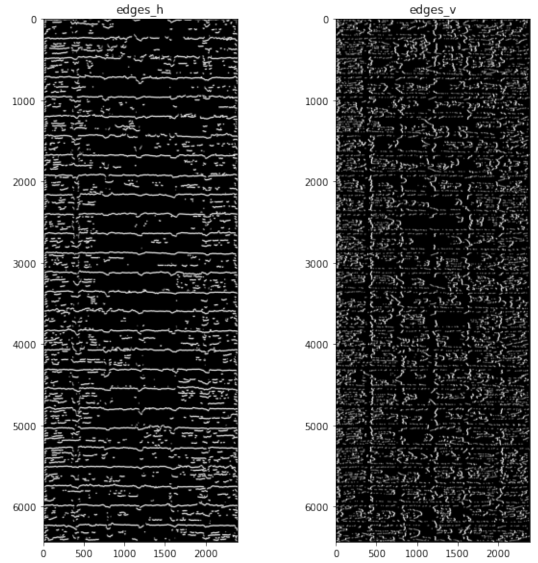

In [15]:

```
# Visualization of Step 3.
fig, axes = plt.subplots(1, 2, figsize=(10, 10))

axes[0].imshow(Transformed_img, cmap=plt.cm.gray)
axes[0].set_title('Transformed_img')
axes[1].imshow(mask, cmap=plt.cm.gray)
axes[1].set_title('Segment_mask(remove_edges)')
```

Out[15]:

```
Text(0.5, 1.0, 'Segment_mask(remove_edges)')
```

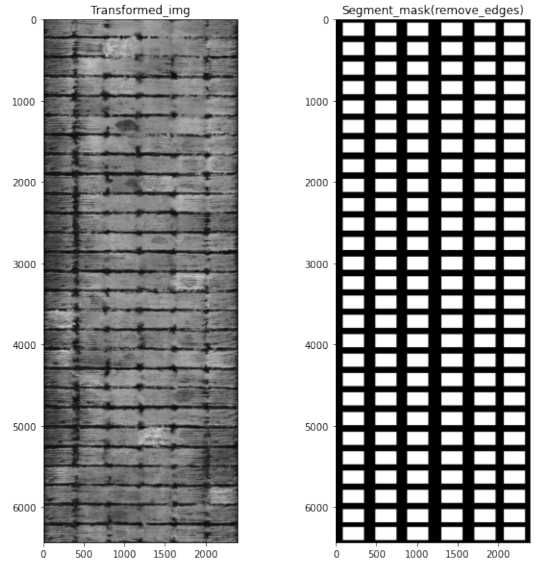

In [ ]:

```
# End of the plot segmentation #
```

Supplemental Methods S4

Code fragments of performing 3D trait analysis at the plot level can be seen as follows:

```
# Import libraries
from laspy.file import File
import open3d as o3d

from skimage import io
from skimage.filters import threshold_local
from skimage.measure import label, regionprops

from matplotlib import pyplot as plt
from scipy import optimize
import pandas as pd
import numpy as np
import cv2
import math
import os
```

In [2]:

```
# Extract traits: Canopy structure indexs of each plot.
def Extract_traits(folder_path):
    # Calculate Canopy structure indexs
    FT_area_index = []
    curvature_index = []
    canopy_cover_index = []
    file_names = []
    for i in range(len(os.listdir(folder_path))):
        file_name = 'plot_'+str(i+1) + '.png'
        file_names.append(file_name)
        # Read img.
        img = io.imread(os.path.join(folder_path,file_name))
        # Fourier Transform.
        # Spectrogram
        fft2 = np.fft.fft2(img)
        shift2center = np.fft.fftshift(fft2)
        # Log Transform.
        log_fft2 = np.log(1 + np.abs(fft2))
        log_shift2center = np.log(1 + np.abs(shift2center))
        X = []
        Y = []
        for j in range(log_shift2center.shape[1]):
            i = int(log_shift2center.shape[0]/log_shift2center.shape[1] * j)
            x = np.sqrt(i**2+j**2) -
np.sqrt((log_shift2center.shape[0]/2)**2+(log_shift2center.shape[1]/2)**2)
            y = log_shift2center[i,j]
            X.append(x)
            Y.append(y)
        X = np.array(X)
        Y = np.array(Y)
        # Gaussian Fitting
        def gaussian(x, amplitude, mean, stddev):
            return amplitude * np.exp(-((x - mean)/4/stddev)**2)
        popt, pcov = optimize.curve_fit(gaussian, X, Y)
        Y = gaussian(X, *popt)
```

```
        # Canopy Structure index
        FT_area = np.trapz(Y,X)
        FT_area_index.append(FT_area)
        # Curvature
        dx_dt = np.gradient(X) # V(x)
        dy_dt = np.gradient(Y) # V(y)
        velocity = np.array([ [dx_dt[i], dy_dt[i]] for i in range(dx_dt.size)])
         # Speed vector.
        ds_dt = np.sqrt(dx_dt * dx_dt + dy_dt * dy_dt) # Speed
        tangent = np.array([1/ds_dt] * 2).transpose() * velocity
         # The unit tangent vector.
        # Take the derivative of the tangent vector and divide by its length to get
the unit normal vector.
        tangent_x = tangent[:, 0]
        tangent_y = tangent[:, 1]
        deriv_tangent_x = np.gradient(tangent_x)
        deriv_tangent_y = np.gradient(tangent_y)
        dT_dt = np.array([ [deriv_tangent_x[i], deriv_tangent_y[i]] for i in
range(deriv_tangent_x.size)])
        length_dT_dt = np.sqrt(deriv_tangent_x * deriv_tangent_x + deriv_tangent_y
* deriv_tangent_y)
        normal = np.array([1/length_dT_dt] * 2).transpose() * dT_dt
        d2s_dt2 = np.gradient(ds_dt) # Acceleration
        d2x_dt2 = np.gradient(dx_dt)
        d2y_dt2 = np.gradient(dy_dt)
        curvature = np.abs(d2x_dt2 * dy_dt - dx_dt * d2y_dt2) / (dx_dt * dx_dt +
dy_dt * dy_dt)**1.5
        curvature_max = np.max(curvature)
        curvature_index.append(curvature_max)
        # Canopy cover
        thresh = threshold_local(img,block_size=175)
        mask = img > thresh
        canopy_area = 0
        label_img = label(mask)
        for region in regionprops(label_img):
            area = region.area
            canopy_area = canopy_area+area
        canopy_cover = canopy_area/(img.shape[0]*img.shape[1])
        canopy_cover_index.append(canopy_cover)
    # rows&columns
    row_index=[]
    column_index=[]
    # The number can be calculated in GUI.
    for i in range(27):
        for j in range(6):
            row_index.append(i+1)
            column_index.append(j+1)

    # Write to csv files.
    # Plot traits.
    dt = pd.DataFrame({'file_names':file_names,'row_id':row_index,
         'column_id':column_index, 'Canopy_index':FT_area_index,'curvature_index':
          curvature_index, 'canopy_cover_index':canopy_cover_index})
    save_path = os.path.join(folder_path,'Canopy_traits.csv')
    dt.to_csv(save_path,encoding="gbk")
    return(save_path)
```

In [3]:

```python
def Biomass_Calculate_Voxel(x,y,z):
    #Voxel Size.
    dist = 0.05 #5cm
    sizeofvoxel = [dist, dist, dist]
    # Length,Width and Height
    pixelWidth = sizeofvoxel[0]
    pixelLong = sizeofvoxel[1]
    pixelHeight = sizeofvoxel[2]
    # Calculate the number.
    rows = np.ceil((np.max(y)-np.min(y))/pixelWidth)
    cols = np.ceil((np.max(x)-np.min(x))/pixelLong)
    heis = np.ceil((np.max(z)-np.min(z))/pixelHeight)
    # Offset removal
    xOffset = x-np.min(x)
    yOffset = y-np.min(y)
    zOffset = z-np.min(z)
    points = np.column_stack([xOffset,yOffset,zOffset])
    points = np.array(points)
    # Calculate the number of voxel which has more than 10 points.
    number = 0
    for i in range(0,int(cols)):
        for j in range(0,int(rows)):
            for n in range(2,int(heis)):
                sub1 =
points[np.where((points[:,0]>=i*dist)&(points[:,0]<(i+1)*dist))]
                sub2 = sub1[np.where((sub1[:,1]>=j*dist)&(sub1[:,1]<(j+1)*dist))]
                sub3 = sub2[np.where((sub2[:,2]>=n*dist)&(sub2[:,2]<(n+1)*dist))]
                if (sub3.shape[0] > 1):
                    number = number + 1
    ThreeD_voxel_index = number/(rows*cols*heis)
    return(ThreeD_voxel_index)

def Biomass_Calculate_Profile(x,y,z):
    # Offset removal
    xOffset = x-np.min(x)
    yOffset = y-np.min(y)
    zOffset = z-np.min(z)
    points = np.column_stack([xOffset,yOffset,zOffset])
    points = np.array(points)
    # New coords:(x,y,z).
    x = points[:,0]
    y = points[:,1]
    z = points[:,2]
    pixelWidth = 0.01 #1cm
    rows = np.ceil((np.max(z)-np.min(z))/pixelWidth)
    # k∈(-3.25,2.25),step = 0.05
    k = 1
    Pt = points.shape[0]
    result = []
    Pcs = 0
    for i in range(0,int(rows)):
        one_layer =
points[np.where((points[:,2]>=i*pixelWidth)&(points[:,2]<(i+1)*pixelWidth))]
        one_layer_number = one_layer.shape[0]
        Pcs = Pcs + one_layer_number
        sub = math.exp(k*Pcs/Pt) * (one_layer_number/Pt)
        result.append(sub)
```

```
    ThreeD_profile_index = np.sum(np.array(result))
    return(ThreeD_profile_index)

def mesh_calculate(x,y,z):
    points_refined = zip(x,y,z)
    # Write to pcd.
    pcd = o3d.geometry.PointCloud()
    pcd.points = o3d.utility.Vector3dVector(points_refined)
    # Downsampling and Alpha shapes
    downpcd = pcd.voxel_down_sample(voxel_size=0.1)
    downpcd.estimate_normals()
    alpha = 0.05
    mesh = o3d.geometry.TriangleMesh.create_from_point_cloud_alpha_shape(downpcd,
alpha)
    mesh.compute_vertex_normals()
    mesh_area = mesh.get_surface_area()
    return(mesh_area)
```

In [4]:

```
def plot_level_traits(folder_path):
    filenames = []
    voxel_index = []
    profile_index = []
    mesh_index = []
    for i in range(len(os.listdir(folder_path))):
        # File name.
        filename = 'plot_'+str(i+1)+'.las'
        filenames.append(filename)
        # Read single plot.
        inFile = File(os.path.join(folder_path,filename), mode='r')
        points = inFile.points
        x,y,z = inFile.x,inFile.y,inFile.z
        # Remove offset
        x = x - np.min(x)
        y = y - np.min(y)
        z = z - np.min(z)
        # Canopy point cloud.
        Canopy_x = x[np.where(z>np.max(z)*0.5)]
        Canopy_y = y[np.where(z>np.max(z)*0.5)]
        Canopy_z = z[np.where(z>np.max(z)*0.5)]
        # calculate
        voxel = Biomass_Calculate_Voxel(Canopy_x,Canopy_y,Canopy_z)
        voxel_index.append(voxel)
        profile = Biomass_Calculate_Profile(Canopy_x,Canopy_y,Canopy_z)
        profile_index.append(profile)
        try:
            mesh_area = mesh_calculate(Canopy_x,Canopy_y,Canopy_z)
            mesh_index.append(mesh_area)
        except RuntimeError:
            mesh_area = 'null'
            mesh_index.append(mesh_area)
    #Write to csv files.
    dt = pd.DataFrame({'filenames':filenames,'voxel_index':voxel_index,

'profile_index':profile_index,'Canopy_surface_index':mesh_index})
    save_path = os.path.join(folder_path,'plot_indexs.csv')
    dt.to_csv(save_path,encoding="gbk")
```

```
    return(save_path)
```
In [5]:
```
def fitting_data(path1,path2,path3):
    def FT_for_XY(img):
        fft2 = np.fft.fft2(img)
        shift2center = np.fft.fftshift(fft2)
        # Log Transform.
        log_fft2 = np.log(1 + np.abs(fft2))
        log_shift2center = np.log(1 + np.abs(shift2center))
        X = []
        Y = []
        for j in range(log_shift2center.shape[1]):
            i = int(log_shift2center.shape[0]/log_shift2center.shape[1] * j)
            x = np.sqrt(i**2+j**2) -
np.sqrt((log_shift2center.shape[0]/2)**2+(log_shift2center.shape[1]/2)**2)
            y = log_shift2center[i,j]
            X.append(x)
            Y.append(y)
        # Diagonal data
        X = np.array(X)
        Y = np.array(Y)
        # Gaussian Fitting
        def gaussian(x, amplitude, mean, stddev):
            return amplitude * np.exp(-((x - mean)/4/stddev)**2)
        popt, pcov = optimize.curve_fit(gaussian, X, Y)
        Y = gaussian(X, *popt)
        # std = popt[2]
        return(X,Y)
    # Repeat 1.
    img1 = io.imread(path1)
    x1,y1 = FT_for_XY(img1)
    # Repeat 2.
    img2 = io.imread(path2)
    x2,y2 = FT_for_XY(img2)
    # Repeat 3.
    img3 = io.imread(path3)
    x3,y3 = FT_for_XY(img3)

    x = (x1+x2+x3)/3
    y = (y1+y2+y3)/3
    return(x,y)
```
In [6]:
```
# Step 1: Extract canopy traits.
images_folder = r"C:\Users\Pix4D-WS\Desktop\test\results\plotimages"
# Calculate and save.
Extract_traits(images_folder)
```
Out[6]:
```
'C:\\Users\\Pix4D-WS\\Desktop\\test\\results\\plotimages\\Canopy_traits.csv'
```
In [7]:
```
# Step 2: Extract plot-level traits: 3DVI,3DPI,Canopy surface index.
point_cloud_folder = r"C:\Users\Pix4D-WS\Desktop\test\results\plot_point_cloud"
# Calculate and save.
plot_level_traits(point_cloud_folder)
```
Out[7]:
```
'C:\\Users\\Pix4D-WS\\Desktop\\test\\results\\plot_point_cloud\\plot_indexs.csv'
```
In [8]:
```
# NO
```

```
path1 = r"C:\Users\Pix4D-WS\Desktop\test\N0_1.png"
path2 = r"C:\Users\Pix4D-WS\Desktop\test\N0_2.png"
path3 = r"C:\Users\Pix4D-WS\Desktop\test\N0_3.png"
x_1,y_1 = fitting_data(path1,path2,path3)
```

In [9]:

```
# N180
path1 = r"C:\Users\Pix4D-WS\Desktop\test\N180_1.png"
path2 = r"C:\Users\Pix4D-WS\Desktop\test\N180_2.png"
path3 = r"C:\Users\Pix4D-WS\Desktop\test\N180_3.png"
x_2,y_2 = fitting_data(path1,path2,path3)
```

In [10]:

```
# N270
path1 = r"C:\Users\Pix4D-WS\Desktop\test\N270_1.png"
path2 = r"C:\Users\Pix4D-WS\Desktop\test\N270_2.png"
path3 = r"C:\Users\Pix4D-WS\Desktop\test\N270_3.png"
x_3,y_3 = fitting_data(path1,path2,path3)
```

In [11]:

```
fig = plt.figure(figsize=(16,8))
ax = plt.axes()
# N0
plt.plot(x_1[np.where(np.abs(x_1)<100)],y_1[np.where(np.abs(x_1)<100)],'b',label='N
0',lw=2.5)
# N180
plt.plot(x_2[np.where(np.abs(x_2)<100)],y_2[np.where(np.abs(x_2)<100)],'g',label='N
180',lw=2.5)
# N270
plt.plot(x_3[np.where(np.abs(x_3)<100)],y_3[np.where(np.abs(x_3)<100)],'r',label='N
270',lw=2.5)

plt.xticks(fontsize=30)
plt.yticks(np.linspace(6.0,9.5,8),fontsize=30)
plt.legend(loc='upper left',fontsize=30)
plt.show()
```

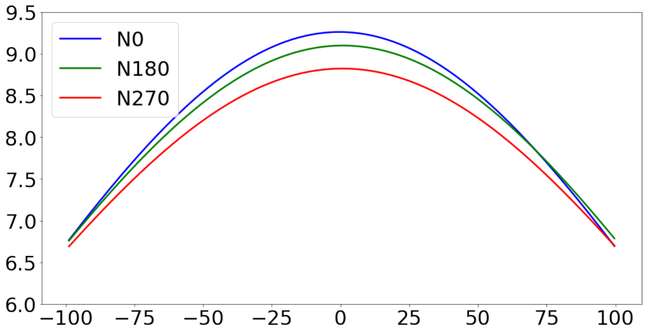


```
In [ ]:
# End of 3D trait analysis #
```

Supplemental Methods S5

Industrial backpack LiDAR Robin hardware

The ROBIN system is an integrated LiDAR Scanner and Inertial Navigation System combined into a single system which is housed within the POD unit. External to the POD is a camera is mounted on a

360° rotating bearing, two GNSS antennae and a touch screen tablet control unit. Its hardware components include protective enclosure (POD), RIEGL VUX-1 Laser Scanner (LiDAR), VUX-1UAV, IGI Compact Inertial Navigation System (INS), CM – Micro-electro-mechanical (MEMS) IMU, Septentrio Dual GNSS Receiver, Camera: FLIR Grasshopper 3 12MP Camera, and Backpack mount. The power of the ROBIN is based on Li-Ion 96 Wh Battery, controlled by rugged tablet computer (GETAC), with input voltage 12-15V DC / Typ 100W. Its operating temperature is 0°C to + 40°C and storage temperature is -40°C to +60°C, with relative humidity：95% non-condensing. ROBIN utilises PAGLink V-Mount batteries. The unique PAGLink system allows up to 8 batteries to be linked for charge or discharge, regardless of their rated capacity or their state of-charge. The hardware components of ROBIN are shown in **Figure S1**. The measurement principle and scanner metrics of its laser scanners are listed in **Table S1**.

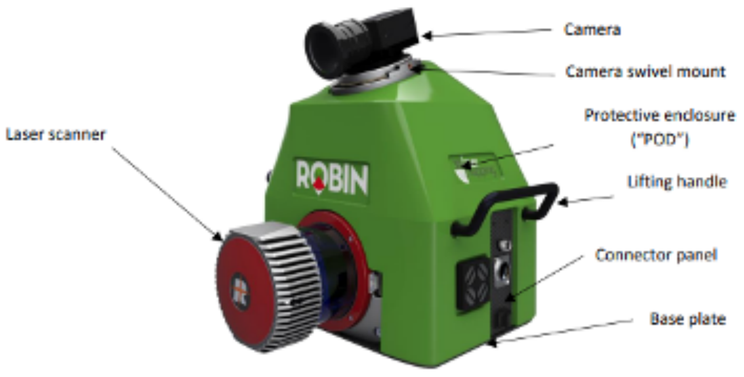

Figure S5.1 The backpack LiDAR hardware device (ROBIN Precision™, 3D Laser Mapping)

Table S5.1 Laser scanner's measurement principle and scanner metrics.

**Measurement types**	Time-of-flight-measurement, echo signal digitization, online waveform processing, and multiple-time-around processing						
**Max. measurement range**<br>For natural targets, ρ ≥ 20%	550 m	400 m	280 m	230 m	200 m	170 m	85 m
For natural targets, ρ ≥ 60%	920 m	660 m	480 m	400 m	350 m	300 m	150 m
**Max. number of targets**	up to 15	up to 15	up to 13	up to 9	up to 7	up to 4	up to 4
**Minimum range**	3 m						
**Accuracy/precision**	10 mm/5 mm						
**Field of View**	330°						
**Max effect measure rate**	Up to 500,000 measurements/sec (@550 kHz PRR & 330° FOV)						

ROBIN systems include TERRAControl Compact, which is IGI's Inertial Navigation System (INS) for the precise determination of position and attitude of the system. The TERRAcontrol Compact system consists of an Inertial Measurement Unit (IMU), integrated Sensor Management Unit (SMU) and GNSS receiver. The TERRAcontrol Compact stores the raw data from the IMU and the GNSS

receiver. It also provides the laser scanner and digital camera with accurate GNSS time stamps for synchronisation of all data streams in post processing. The ROBIN system includes a high-resolution colour camera, the FLIR Grasshopper3. The Grasshopper3 has a capture rate of up to 4 frames per second. Each image is accurately time-stamped by the GNSS/INS and can be accurately geo-referenced.

Supplemental Movie Legend

Supplemental Movie S1. Movie showing how to use CropQuant-3D in action, which includes loading the data, pre-processing the point clouds, filtering ground-level and above-ground 3D points, generating 2D CHM, conducting automated plot segmentation, and measure 3D traits for the segmented plots.
